# Deep Learning Analysis of Routine Preoperative Knee Radiographs for Five-Year Prediction of Contralateral Arthroplasty

**DOI:** 10.3390/jimaging12100474

**Published:** 2026-09-30

**Authors:** Samer G. Salman, Rohan A. Phadke, Zane G. Salman, Ryan T. Zeitouny, Sai M. Yedupati, Joshua Ong, Alireza Tavakkoli, Sainyam Galhotra, Ajay Tripuraneni, James Rizkalla

**Affiliations:** 1 School of Medicine, Baylor College of Medicine, 1 Baylor Plaza, Houston, TX 77030, USA; 2 College of Natural Sciences, The University of Texas at Austin, Austin, TX 78712, USA; 3 Department of Biomedical Sciences, Texas A&M University, College Station, TX 77843, USA; ryanzeitouny@tamu.edu; 4 College of Engineering and Applied Sciences, University of Cincinnati, Cincinnati, OH 45221, USA; yedupasv@mail.uc.edu; 5 Massachusetts Eye and Ear, Department of Ophthalmology, Harvard Medical School, Boston, MA 02114, USA; 6 Department of Computer Science, University of Nevada, Reno, NV 89557, USA; 7 Department of Computer Science, Cornell University, Ithaca, NY 14853, USA; 8 Department of Orthopaedic Surgery, Baylor University Medical Center, Dallas, TX 75246, USA

**Keywords:** total knee arthroplasty, contralateral knee, deep learning, radiography, image interpretability, risk prediction, osteoarthritis

## Abstract

**Background**: Before unilateral total knee arthroplasty (TKA), the contralateral radiograph is reduced to a Kellgren–Lawrence grade. We asked what the image adds, what the model reads, and how many views are needed. **Methods**: A multi-view DenseNet121 survival network predicted 5-year contralateral arthroplasty from routine radiographs, against Cox models over 11 clinical variables with and without the dataset-inferred grade, on a once-read test split. **Results**: In 741 test patients (106 events), the primary contrast put the combined model’s 5-year censoring-weighted AUROC at 0.844 against the clinical model’s 0.680 among the 740 patients that received both scores (difference 0.164, 0.087 to 0.240). Post hoc, a frontal radiograph alone read 0.837, matching the multi-view arms because 56.6% had only a frontal film; withholding lateral and sunrise views cost 0.078 (0.012 to 0.142) in the 315 patients with both. Grad-CAM put 37.8% of attribution in a joint region covering 25.0%; occluding it left 0.800. **Conclusions**: The preoperative radiograph adds prognostic information beyond the clinical record. Exploratory analyses place almost all of it in one frontal film and beyond an automated grade, not a radiologist’s read. These internal analyses require external validation.

## 1. Introduction

Knee osteoarthritis is bilateral in most patients who have surgery. Between 37 and 45% of patients undergoing unilateral total knee arthroplasty (TKA) ultimately require contralateral replacement, with cumulative incidence reaching 40% at 8 years in the Osteoarthritis Initiative [1,2,3]. Whether a second operation is needed, and when, influences surgical timing, rehabilitation and staging [4,5]. A radiograph of the contralateral knee is almost always acquired before the first operation, so the question is not whether an image exists but how much of the answer is in it.

In practice, that image is reduced to structured descriptors. Kellgren–Lawrence grade, malalignment, body mass index, age and symptoms have all been associated with subsequent contralateral arthroplasty, and radiographic severity is usually the strongest [1,3,6,7,8]. The grade is a five-level ordinal summary of a 512-pixel field. It compresses joint-space width, sclerosis, osteophytes, alignment and peri-articular bone into one number, is applied inconsistently between readers, and is increasingly assigned by an automated classifier rather than a radiologist. Whether the part of the radiograph the grade discards carries prognostic information is untested for this outcome.

Machine-learning studies of knee osteoarthritis prognosis usually predict a radiographic endpoint: osteoarthritis development where the contralateral knee is the target [9], or progression elsewhere [10,11]. Deep-learning models have predicted TKA from radiographs and magnetic resonance imaging with high discrimination, for the imaged knee in research cohorts with standardized acquisition [12,13]. A meta-analysis pooled C-indices of 0.71 to 0.81 while noting heterogeneous outcomes and almost no external validation [14], and orthopedic and spine reviews describe the same translational gap [15,16,17]. What that work lacks is not new architecture but evidence. No study asks whether the film that has already been taken of the other knee predicts a second arthroplasty in a health-system population or isolates what the image adds inside a fixed grade. Discrimination is reported without reporting what the network reads: attribution maps are common, quantitative attribution and occlusion experiments are rare, and leakage controls on burned-in text and borders are rarer still. The imaging requirement is seldom examined, so how many radiographs a deployed model needs is open.

This study asks three imaging questions. Can a routine frontal radiograph predict contralateral arthroplasty beyond conventional grading, within a fixed grade rather than across grades? What does the model read, and can any of the predictions be attributed to laterality markers, technique text or the border rather than anatomy? Are multiple views necessary, and if one suffices, is that a property of the image or of the population imaged?

We developed and internally validated a discrete-time survival model predicting contralateral arthroplasty from the preoperative radiograph in a health-system population, with a test split locked before fitting and read once. We benchmarked the image against routine clinical variables with and without a structured severity grade, withheld individual views from the frozen network and read its per-view attention, quantified attribution inside a predefined tibiofemoral region, occluded anatomic regions, and ran border-widening, marker-blanking and parameter-randomization controls.

## 2. Methods

Details are in the Supplementary Materials Methods Section.

### 2.1. Radiographic Dataset and Image Selection

We used the Emory Knee Radiograph (MRKR) dataset [18], a de-identified single-institution archive linked to procedure, diagnosis and pain records. Eligible patients had a unilateral index arthroplasty (CPT 27447) at age 40 or older, no prior contralateral arthroplasty, and an interpretable contralateral radiograph 1 to 730 days beforehand. Follow-up began at a day-90 landmark; the outcome was contralateral arthroplasty within 5 years and censored at day 1826. Frontal, lateral and sunrise views entered under a fixed preference order.

The 3709 qualifying patients (Supplementary Materials Figure S2) were partitioned once into locked, stratified 70% training, 10% validation and 20% test sets. The test split took no part in fitting or checkpoint selection, was scored exactly once, and is the source of every estimate here.

We specified every imaging, interpretability, view-ablation, within-grade and robustness analysis reported below after that single read, so all are exploratory. Supplementary Materials Table S6 maps each claim here to the deviation register entry governing it.

### 2.2. Image-Quality and Laterality Verification

The index operative side was coded on the record for 1881 patients and recovered by a deterministic pre-index rule for 1828 patients, 49.3% of the cohort. The dataset’s model-inferred annotations also decide eligibility, view label and, through a horizontal-flip flag, which half of a bilateral film is cropped; no DICOM tag records that flag.

Three manual reviews are required before the locked split is opened. The 200-patient laterality audit and the 400-image two-reviewer review (its sample was fixed before scoring: the pre-specified 200 records and a 60-record test-split extension made the sample size 260, with 101 of them being test patients). Two reviewers scored it while blind to the derived label, with no disagreement requiring adjudication, under a 2% error rule and a scorer fixed in advance (Supplementary Materials Methods Section S14).

### 2.3. Image Preprocessing

On a bilateral film the contralateral half was taken with a 2% inset from the midline; a square region centred on the collimated field was resampled to 512 by 512 pixels and mirrored to read as a left knee. The outer 31 pixels were zeroed, blanking 22.75% of the crop, and a marker detector blanked saturated text-like blobs in the retained region, running after border zeroing, so the band can end up non-zero.

### 2.4. Network Architecture and Multi-View Aggregation

The image arms share one ImageNet-initialized DenseNet121 [19] encoder. Each crop is encoded with a learned view embedding, and a masked attention pool returns one patient-level representation; a linear head emits ten six-month interval hazards that combine into a 5-year risk under a censoring-aware discrete-time likelihood. The fusion arm concatenates that pooled vector with the frozen clinical vector before the head. The complete radiographic preprocessing and modelling workflow is summarized in Figure 1.

ConvNeXt-Tiny [20] was the pre-specified encoder and did not fit these training data under the one configuration tried before the split was read: in the ladder behind every number here it reached a mean best training negative log-likelihood of 0.607 over five seeds on frontal crops, against DenseNet121’s 0.371. DenseNet121 became the primary encoder and ConvNeXt-Tiny the single robustness architecture, so every encoder comparison is exploratory.

Because one configuration is thin evidence for abandoning an architecture, we ran a staged ConvNeXt-Tiny hyperparameter search at revision, ranked on training negative log-likelihood alone and reading no split: 12 configurations of encoder learning rate and layer-wise decay at one seed and then the single best configuration over five seeds, moving warmup, stochastic depth and input resolution one factor at a time (Supplementary Table S8). A ResNet-50 frontal-view arm on the DenseNet121 schedule was trained as a second architecture, reading no test data.

### 2.5. Training and Internal Validation

Every arm used early stopping on validation loss, averaging five pre-specified seeds on the hazards. A horizon-specific complementary log-log recalibration was fitted on the validation split alone, frozen, and applied to the image arms only; the Cox comparators are published as fitted. Every risk here is on that recalibrated scale, a strictly monotone transform. The sealed-read artefact records no interpreter, device or precision; the published environment file describes the revision workstation.

### 2.6. Image Interpretability and Leakage Controls

Attribution used gradient-weighted class activation mapping [21] on 858 frontal test crops and integrated gradients [22] on 230 crops, samples that are not interchangeable. Occlusion set occluded pixels to zero. Masking sensitivity blanked every residual marker-like blob under the looser criterion, so it bounds above, and widened the band to 62 and 93 pixels. Attribution was re-run after cascading parameter randomization; the label-randomization control requires retraining and was not performed. Views were withheld at input on the 315 test patients with a frontal and a non-frontal crop.

### 2.7. Imaging Robustness and Ablation Analyses

Discrimination was re-estimated within strata of image acquisition and quality (Supplementary Table S2), each a mask over existing predictions, not a new read. Every stratum also carries horizon-free Harrell concordance, because only 162 of the 741 test patients reach day 1826, leaving the horizon-specific estimator without controls; the pre-specified 50-event floor stands unchanged.

We ran a fusion ablation: mean and max pooling replaced masked attention at inference on both frozen all-views ensembles, the combined arm and the image arm, re-scoring the already-read split. Concatenation changes the head’s input width, so that variant was retrained for both image arms and reported on validation alone. An inference-time swap measures how far the learned representation depends on attention weighting, not what a model trained under mean pooling would have reached.

### 2.8. Clinical Comparators, Missing Data and Statistical Analysis

The following two frozen clinical comparators anchor the analysis: a ridge-penalized Cox model over eleven routinely recorded pre-index predictors, and a second adding the dataset-inferred contralateral Kellgren-Lawrence grade. Two predictors depart from their pre-specified definitions, and the ridge penalty sits at the unwidened edge of its grid.

Missing predictor values took training-split medians and modes, frozen and replayed on validation and test, so no imputation statistic crossed a split boundary. The following two predictors are incompletely observed: the maximum pre-index knee pain score, missing for 7.2% and filled at 7.0, and the inferred contralateral grade, missing for 3.9%. The grade is never imputed: the comparator that uses it is fitted only where it is observed; a pain-score missingness indicator was dropped as exactly aliased with the knee-pain flag. We assume values are missing at random given the observed predictors, an assumption these data cannot test; a complete-case analysis agreed (Supplementary Methods S10).

The pre-specified primary estimand is the difference between combined and clinical models in censoring-weighted time-dependent AUROC at days 365, 730 and 1825, with 95% intervals from a paired 2000-replicate bootstrap. Every contrast uses the intersection of the two arms it compares, not the gap between two marginal levels. The primary contrast is unadjusted, each secondary family is Benjamini–Hochberg adjusted within itself, and estimates below 50 events are suppressed.

Within radiographic severity we report incidence by risk tertile inside each grade stratum, and Cox models adding a standardized risk score to it. Decision-curve analysis [23] was pre-specified in kind, but its estimator, thresholds and arms were fixed after the single read; only 268 of the 741 test patients are classifiable at 5 years, so any classification cell describes that subset.

Reporting follows TRIPOD + AI [24] and CLAIM [25]; completed checklists accompany this report and the sample was checked against Riley criteria [26]. We claim no external validation [27].

## 3. Results

Details are in Supplementary Results.

### 3.1. Cohort and Image Data

Of 3709 landmark-cohort patients, 741 with 106 events formed the locked test split and 2968 with 427 events the development split (Table 1). Preprocessing wrote 6071 crops, 1216 for 740 of the 741 test patients; the one without an image was event-free, so all 106 test events survive. Of those 740, 419 (56.6%) contributed a frontal radiograph only.

### 3.2. Manual Review and Burned-In Markers

Two reviewers with musculoskeletal imaging experience independently scored the 400-image sample on six items. Raw agreement was 95% to 97.9%, Cohen kappa 0.81 to 0.89, and the critical-error rate 0.5% to 1.0%, inside the pre-specified 2% threshold. Laterality, assessable on the 318 panels carrying a full film, was correct on at least 99% (Supplementary Methods S2).

Laterality audit found agreement between the deterministic index-side recovery rule and manual adjudication in 196 of 200 sampled development patients (98.0%; Wilson 95% CI, 95.0% to 99.2%), a 2.0% error rate. It contained no test patients, so it validates the recovery rule on development patients rather than the 371 test patients whose index side was recovered by rule. In the outcome-record audit, which checked the billing record rather than the image, the index side the cohort used agreed with adjudication in 260 of 260 sampled patients (100.0%; Wilson 95% CI, 98.5% to 100.0%) and in 101 of 101 test-split patients (100.0%; 96.3% to 100.0%; Supplementary Results S13). The same audit’s design-weighted label error rate, 0.00%, did not exceed the pre-specified 2% threshold, so the cohort lock was retained.

Automated scans gave 26.4% of 500 training crops before masking, 16.7% of 72 finished development crops after it, 18.5% of all 1216 finished test crops, and a masked band that is not exactly zero on 145 crops (11.9%).

### 3.3. The Radiograph Against the Clinical Record

At 1825 days the combined model out-discriminated the clinical model in censoring-weighted time-dependent AUROC by 0.164 (95% CI 0.087 to 0.240; *p* < 0.001) on the 740 patients both score, where the clinical model reads 0.680, not the 0.682 it reads on all 741 (Table 2 and Table 3, Figure 2).

The single frontal radiograph carries almost all of that advantage: 0.837 (0.785 to 0.888), with a Harrell concordance of 0.779 (0.738 to 0.817). Against the clinical model the paired difference was 0.162 (0.088 to 0.233; *p* < 0.001) on the 734 patients both arms score, where the clinical model reads 0.675. Against that model plus the inferred grade it was 0.114 (0.060 to 0.170; *p* < 0.001) on the 707 both score; both single-view contrasts are post hoc. An automated severity grade does not capture what the network reads off the same film.

**Figure 2:**
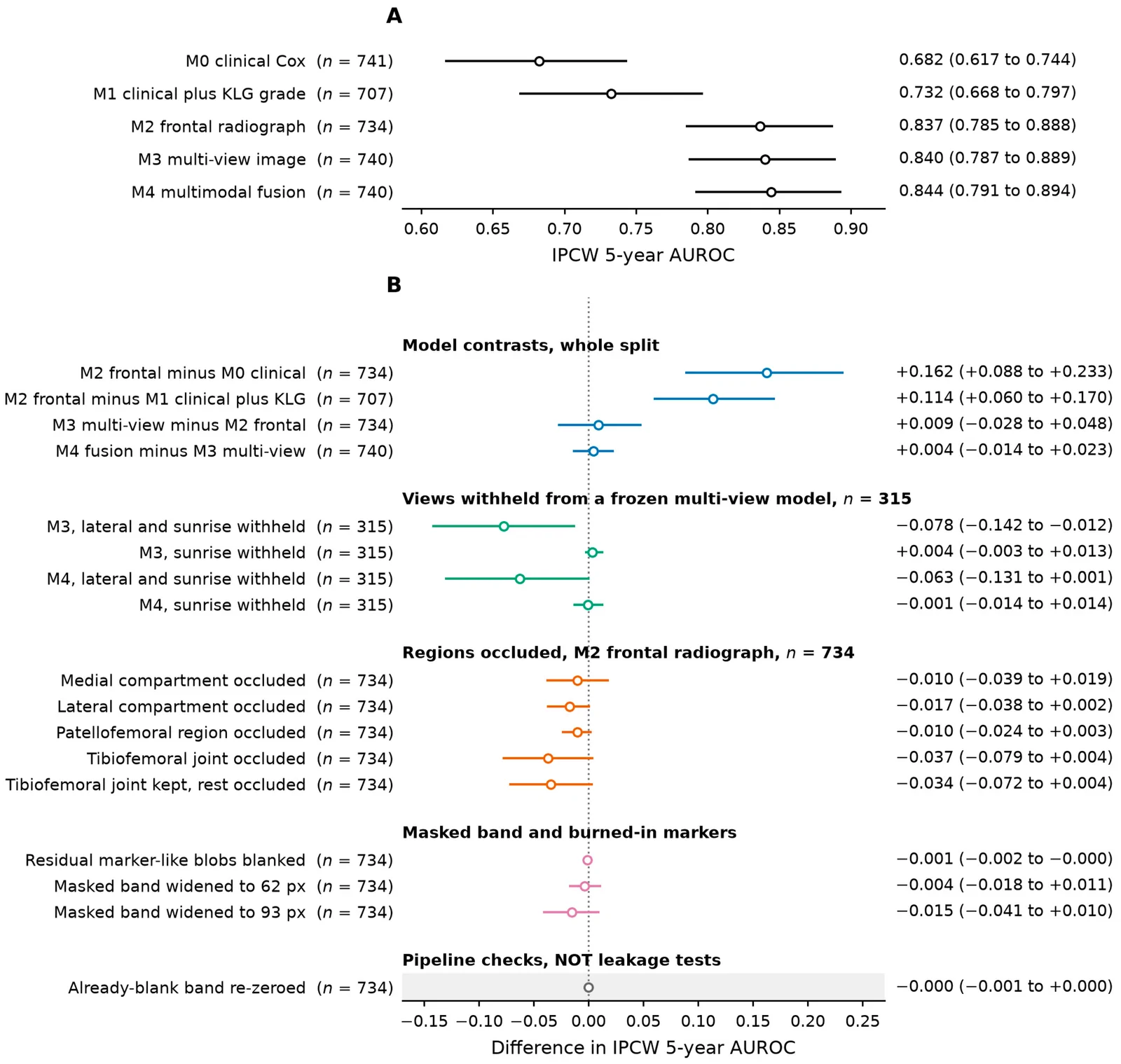
Discrimination of the clinical and imaging models, and the effect of withholding views, occluding regions and widening the masked band. (**A**) gives the 5-year IPCW cumulative dynamic time-dependent area under the receiver operating characteristic curve for five of the nine model arms on the test split, with 2000-replicate percentile bootstrap 95 percent intervals. Each arm is estimated on the patients it can score and those denominators differ; they are printed beside each row. (**B**) gives differences on the same scale, each from a paired bootstrap on the patients both conditions score, with the number of paired patients printed beside each row. In (**B**), the vertical dotted line marks no difference (0.00), and the colours group the contrast families (model contrasts, views withheld, regions occluded, masked band and burned-in markers, and the pipeline check) without carrying any quantitative meaning; the grey shading marks the row identified below as a pipeline check. The model contrasts compare separately trained arms across the whole split. The view-withholding rows take one frozen multi-view network and remove views at input, so they are restricted to the 315 patients who contributed both a frontal and at least one non-frontal radiograph; they answer what a view contains, which is a different question from what a model that was trained without it achieves. The region and masking rows re-score the frozen frontal-view network on perturbed images. Every anatomic occlusion interval drawn here crosses zero, so the compartments must not be ranked against one another. The underlying table also holds a mean-fill variant of each of the two tibiofemoral conditions, which are not drawn because the value the occluded pixels are filled with is a nuisance choice rather than a finding. One of them is the only anatomic condition in the table whose interval excludes zero: keeping the tibiofemoral joint alone and filling the rest with the image mean lowers 5-year discrimination by 0.051, with a 95 percent interval of 0.010 to 0.092. It is reported here rather than left in the table because it is the condition most favourable to the model, and it does not licence a ranking either: the anatomic conditions were not adjusted for multiplicity, and the zero-fill version of the same condition crosses zero. The row in the shaded block is a pipeline check and not a leakage test. The preprocessing pipeline already zeroes the outer 31 pixels, so re-zeroing that band leaves 677 of 734 patients bit-identical, which shows the perturbation path is exact. The pre-specified border-band-only control is not drawn and is withdrawn from the paper: that band is already zero, so supplying it alone hands the network a near-constant input, and the discrimination that follows measures the destruction of the input rather than anything about burned-in text. The evidence about text is the two widened bands and the residual-marker row, all of which perturb pixels that do carry signal and all of which are upper bounds. The single-view contrasts, the view-withholding rows and the occlusion rows are post hoc analyses of an already-read split and are reported as exploratory; the deviation register carries them. These are out-of-sample estimates: the test split was read once, after every model and every hyperparameter choice had been frozen.

### 3.4. What Additional Views Contribute

Across the whole test set a single frontal radiograph matched the multi-view and multimodal arms (0.840, 0.844). The paired contrasts are 0.009 (−0.028 to 0.048; *p* = 0.61) for the all-views against the frontal-view model and 0.004 (−0.014 to 0.023; *p* = 0.64) for the combined against the all-views model, both spanning zero. Those contrasts say nothing about a second film, because 56.6% contributed only a frontal film.

Where additional views existed they were not redundant: withholding the lateral and sunrise views from the frozen all-views network cut discrimination from 0.824 to 0.746 (−0.078, 95% CI −0.142 to −0.012; *p* = 0.024) on the 315 patients with a frontal and a non-frontal crop. Withholding the sunrise view alone changed nothing measurable on the 37 of 740 patients who have one; the attention pool gave the lateral view most of its weight wherever one existed (Supplementary Table S4).

### 3.5. Where the Model Looks, and What It Is Not Reading

Attribution is enriched in the tibiofemoral joint but not confined to it. Across all 858 frontal test crops from 734 patients, Grad-CAM placed 37.8% of its mass inside a joint region occupying 25.0% of the image, a 1.5-fold enrichment; integrated gradients, on 230 crops from 200 patients, placed 55.8% there, a 2.2-fold enrichment (Figure 3). Enrichment rises to 1.77-fold on a per-image region re-centred on each crop’s own joint line (Supplementary Table S3, Supplementary Results S6).

The signal is distributed: blacking out the joint region reduced discrimination to 0.800 (−0.037, 95% CI −0.079 to +0.004) and supplying only that region gave 0.802 (−0.034, −0.072 to +0.004). Neither approach chance: the periphery carries nearly as much signal as the joint. Every anatomic occlusion interval crosses zero except one mean-fill variant, so no compartment is ranked.

Leakage from burned-in text is bounded at roughly one thousandth of AUROC. Blanking every residual marker-like blob, an upper bound because saturated bone edges share the detector’s signature, changed discrimination by −0.00096 (95% CI −0.00230 to −0.00008), and widening the band to 62 and 93 pixels changed it by −0.0038 (−0.0180 to +0.0115) and −0.0154 (−0.0414 to +0.0102), intervals that bound an undetected effect rather than measure one. The pre-specified border-band-only control cannot test leakage here: preprocessing already zeroes that band, so the model sees a near-constant input and we report no result from it.

### 3.6. Prediction Within Kellgren–Lawrence Strata

The inferred grade was recorded for 707 test patients carrying 98 events, so only the advanced stratum clears the 50-event floor (0.802, 0.716 to 0.880).

Tertiles of predicted risk formed within a single grade separated observed 5-year incidence sharply, from 11.3% (5.7 to 21.5) to 52.7% (39.2 to 67.5) among grade 3 to 4 knees (log-rank *p* = 2.1 × 10^−8^); the 144 patients at grades 0 to 1 carry three events, an uninformative cell (Supplementary Table S5, Figure 4). With the grade held in a Cox model, each standard deviation of the risk score carried a hazard ratio of 3.01 (2.26 to 4.00) against the grade’s own 1.04 (0.81 to 1.34; *p* = 0.75). These estimates rest on a model-inferred grade.

### 3.7. Analyses Added at Revision

We ran three analyses specified at revision (Supplementary Methods S11–S13), and all three are reported below. The sweep, the ResNet-50 arm and the concatenation retrain read no test data; the pooling swaps re-score the read split.

The best swept ConvNeXt-Tiny configuration reached a five-seed mean training negative log-likelihood of 0.274, against 0.371 for DenseNet121 on identical crops, so demoting it was premature. A ResNet-50 frontal-view arm reached 0.499 in training, with per-seed minima from 0.452 to 0.610, and a validation AUROC at 1825 days of 0.803 (0.726 to 0.876), where DenseNet121 reads 0.828 (0.751 to 0.898) and ConvNeXt-Tiny 0.700 (0.607 to 0.791) on the same 370 patients (Supplementary Tables S8 and S9). One of its five seeds is degenerate: it retained epoch 0, stopped after nine epochs, and gave this arm the widest validation seed spread in the ladder.

The pooling ablation covers all 740 test patients (106 events) and the 373 with more than one crop, where pooling is not the identity (Supplementary Table S10). On the whole roster, mean and max pooling were marginally higher in the image arm, by +0.0133 (95% CI +0.0018 to +0.0255; *p* = 0.018) and +0.0129 (+0.0019 to +0.0247; *p* = 0.015); there the swap also moves multi-crop patients’ risks relative to single-crop patients, whose predictions do not change. Every other interval includes zero: +0.0093 and +0.0093 in the combined arm on the whole roster, and +0.0043 and +0.0037 in the combined arm and +0.0145 and +0.0138 in the image arm on the multi-crop patients. On validation, where estimates are optimistic, retrained concatenation read 0.841 in the image arm and 0.846 in the combined arm, against 0.833 and 0.835 under attention. Attention pooling was not superior on any roster or split, and the primary estimates do not depend on the aggregator.

Every validation figure here is optimistic, because each checkpoint was selected on the split that scores it; our comparison table suppresses such contrasts.

### 3.8. Robustness Strata

Discrimination was similar across weight-bearing status, localization method, inversion, half-selection and masking, but outside masking only one level per family carried 50 events, so each compares one stratum against the whole split (Supplementary Figure S5, Supplementary Table S2). Masking, the only family with two estimable levels, is confounded with view availability. Era, equipment, manufacturer and site could not be evaluated: no acquisition tag was released, and the study date carries an unconfirmed per-patient shift.

### 3.9. Calibration and Clinical Usefulness

After frozen recalibration the combined model’s calibration slope at 1825 days was 1.02, with a 7.0 percentage-point under-prediction in the large; the frontal-view model gave 1.01 and 5.6 points (Table 3, Figure 4).

In decision-curve analysis (Supplementary Figure S3), at a 20% threshold the frontal-view model avoided 34.5 unnecessary interventions per 100 screened against the clinical model’s 15.7. Both rest on each arm’s own patients, not a paired contrast; the paired difference in net benefit, on the 734 both arms score, is 0.049 (0.017 to 0.082; *p* = 0.003). These too are exploratory.

## 4. Discussion

A single preoperative frontal radiograph of the contralateral knee out-predicted the routine clinical record by 0.162 of 5-year censoring-weighted AUROC (95% CI 0.088 to 0.233; 0.837 versus 0.675 on the 734 patients both arms score; Harrell concordance 0.779). The advantage survives inside a fixed grade, which no between-model contrast can manufacture: each standard deviation of image risk carried a hazard ratio of 3.01 (2.26 to 4.00; likelihood-ratio *p* = 5.1 × 10^−16^), while the grade’s own effect collapsed to 1.04 (0.81 to 1.34; *p* = 0.75). The grade is itself model-inferred, so this is information beyond an automated grade, not beyond a radiologist’s read.

Grad-CAM placed 37.8% of its attribution mass in a tibiofemoral region occupying 25.0% of the image and integrated gradients placed 55.8% yet blacking that region out still left 0.800 against 0.837. The signal is enriched at the joint and not confined to it, and we rank no compartment: every anatomic occlusion interval crosses zero. Burned-in markings do not explain it either. Blanking residual markers moved discrimination by −0.00096 (95% CI −0.00230 to −0.00008) and two band widenings by intervals crossing zero, bounding an undetected effect rather than measuring one, on crops where markers survive in 18.5% of 1216 (Figure 1 and Figure 3).

A frontal-only model matched the multi-view and multimodal arms across the test set (0.837 versus 0.840 and 0.844) because 419 of 740 patients (56.6%) contributed a frontal film and nothing else. Where a second film existed it was not redundant: withholding the lateral and sunrise views cost 0.078 (95% CI 0.012 to 0.142; *p* = 0.024) in the 315 patients who also had a non-frontal crop. A single-view model is the right instrument for this population, not evidence that the other views are uninformative.

We claim no algorithmic novelty. DenseNet121, Grad-CAM, integrated gradients and attention pooling are mature and deliberately off the shelf; settled tools keep the evidentiary claims readable, because any difference belongs to the question rather than an untested architecture. The following four contributions are ours: the clinical question, contralateral arthroplasty predicted from the routine preoperative film in a health-system rather than a research cohort; the within-grade design, which no between-model contrast can manufacture; quantitative attribution, occlusion and leakage controls reported together; and the view-necessity decomposition, which separates a population effect from an information effect. Supplementary Table S7 sets these against prior work [9,10,11,12,13,14].

One of those choices was wrong. We replaced the pre-specified ConvNeXt-Tiny encoder with DenseNet121 after a single configuration failed to fit and registered that as a trade; before the sweep ran, we committed in writing that a sweep which made ConvNeXt-Tiny converge would make the abandonment premature and that this Discussion would have to say so. It converged to a lower training negative log-likelihood than DenseNet121 on identical crops, so on that pre-declared rule the abandonment was premature: an error of judgement rather than a trade. The abandoned run had never improved its fit once its encoder started training, its training loss bottoming out in every seed in the last epoch before the backbone thawed and rising thereafter, so what it measured was a learning rate rather than an architecture. No reported estimate moves, because nothing was retrained and no held-out split was read, but the robustness arm the protocol asked for was never a fair test of the architecture it names.

These findings support stratification, not quantification. After recalibration the multimodal model’s 5-year calibration slope was 1.02, yet it under-predicted absolute risk by 7.0 percentage points in the large. Counselling on the timing or staging of a second procedure [28,29,30] would therefore have to rest on relative risk and observed incidence within a predicted-risk band, not the predicted probability. Any eventual use would be informational and subordinate to the reading clinician, who needs musculoskeletal imaging expertise, selects the input radiograph, and weighs the predicted risk against their own read.

The outcome is a healthcare-utilization event, not a biological one. A knee reaches arthroplasty when structural disease, symptoms, surgeon judgement, patient preference and access align, so the model predicts who gets operated on within 5 years, not whose joint degenerates. Access already differs by race and sex [31,32], so part of what the network learned is a signal about the health system that produced the labels, and it travels only as far as that system does. Transportability is limited by more than case mix: where referral and scheduling differ, the model can be miscalibrated either way.

Every imaging, leakage, view-ablation, within-grade and robustness result is post hoc on an already-read test split. The index side was recovered by rule for 1828 of 3709 patients, and the audit agreeing on 98.0% held no test patient. On the test split the index side was checked against the billing record, where it agreed in 101 of 101 patients, and never against the image. De-identification shifted every study date by a per-patient random amount; era is also confounded with follow-up capture, since median follow-up on this split falls from 1826 days in 2007 to 2011 to 430 days in 2017 to 2021 while the share observed through 5 years falls from 97.2% to 1.0%. No temporal split is constructible from these dates, and no equipment, manufacturer or site tag exists, so no acquisition stratum is evaluable. Subgroup analyses were underpowered to test performance across race and sex. This is a single-institution retrospective development study with no external or temporal validation, so transportability is untested [33,34,35,36]; multi-institution work is the remedy, and knowledge distillation [37] and personalized federated learning [38] are routes to it that avoid pooling images across sites. Mortality was unavailable as a competing event, so every incidence estimate here censors at death and Kaplan–Meier overstates cumulative incidence relative to Aalen–Johansen [39]; correcting for the competing risk of death would move observed incidence toward the predictions and partly offset the 7.0-point under-prediction in the large. We give the direction, not a magnitude. Nothing here supports clinical use: external validation, competing-risk and powered fairness analyses, and evidence that acting on predicted risk changes outcomes must precede it.

## 5. Conclusions

The preoperative contralateral radiograph adds prognostic information beyond the clinical record. In the pre-specified primary contrast the combined model out-discriminated the clinical model by 0.164 of censoring-weighted AUROC at 1825 days (95% CI 0.087 to 0.240; *p* < 0.001) on the 740 patients both score. Exploratory analyses run after the split was read place almost all of that advantage in a single frontal radiograph and separate observed 5-year incidence from 11.3% to 52.7% within grades 3 to 4. Attribution concentrates at the joint without being confined there. The model orders risk better than it quantifies it. External validation and prospective evaluation must precede deployment.

## Figures and Tables

**Figure 1:**
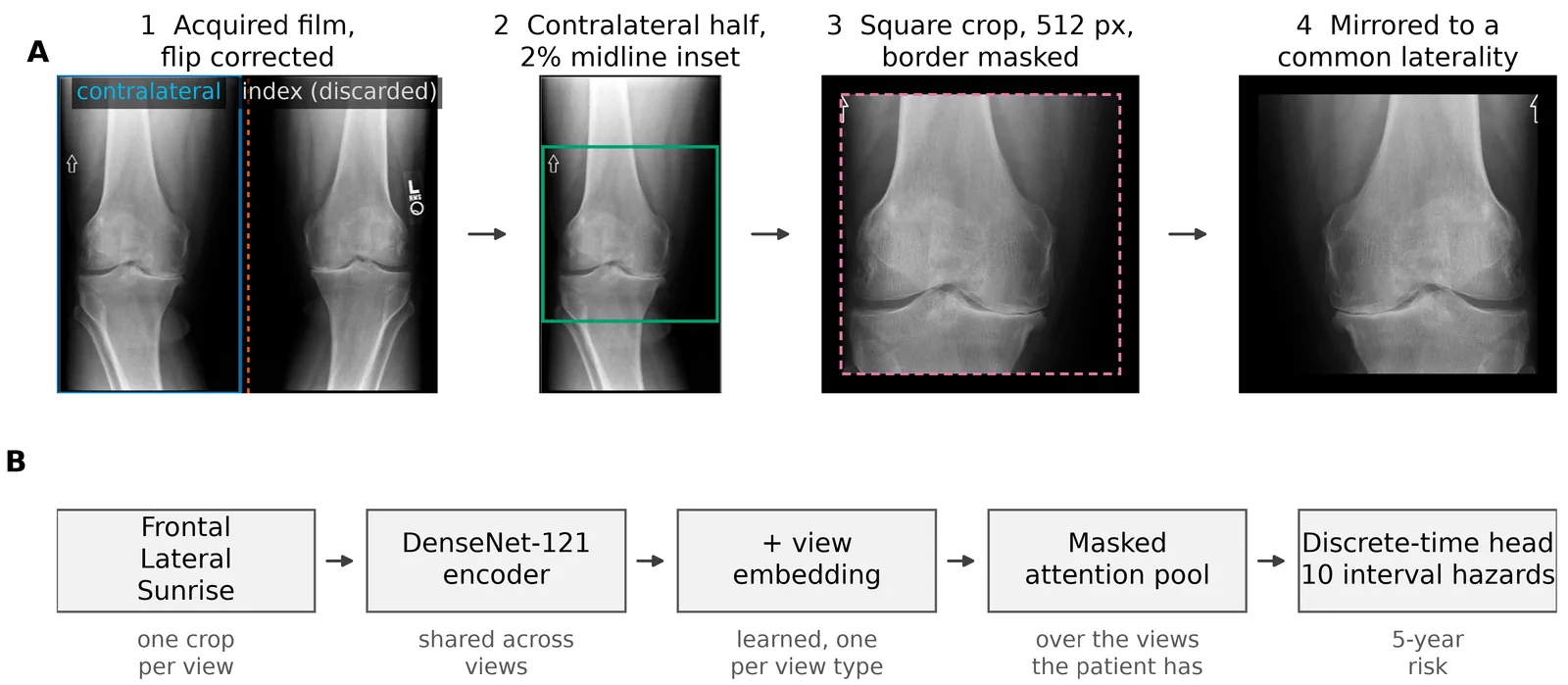
Imaging and modelling workflow, from the acquired radiograph to the 5-year hazard. (**A**) follows one bilateral weight-bearing frontal radiograph through preprocessing. Tile 1 is the flip-corrected film: the blue box marks the contralateral half that is kept, the orange dashed line the midline from which a 2% inset is taken, and the white arrow, the letter “L” and the circle beside it are radiopaque laterality markers burned into the film at acquisition, not annotations added here. Tile 2 is that half, the green box giving the square crop the pipeline computes; resampling it reproduces the shipped crop at a Pearson correlation of 0.99. Tile 3 is the 512-pixel crop, the pink dashed box enclosing the region retained inside the zeroed 31-pixel border band. Tile 4 is that crop mirrored so every knee reads as a left knee, and is the array the network reads. Black arrows give the order of processing. A burned-in marker survives inside the retained region, clipped by the band at the upper left of tile 3 and, after mirroring, the upper right of tile 4; this is a true property of the input, and Figure 2 bounds what such markers are worth. (**B**) gives the model: grey boxes are the stages each crop passes through, from a shared DenseNet-121 encoder through a learned view embedding and a masked attention pool over the views the patient has, to a discrete-time head emitting 10 interval hazards from which the 5-year risk follows. The film was recovered from a reviewer quality-assurance panel, the source DICOM archive no longer being held, so tiles 1 and 2 are a downsampled copy; tiles 3 and 4 are the shipped crop itself.

**Figure 3:**
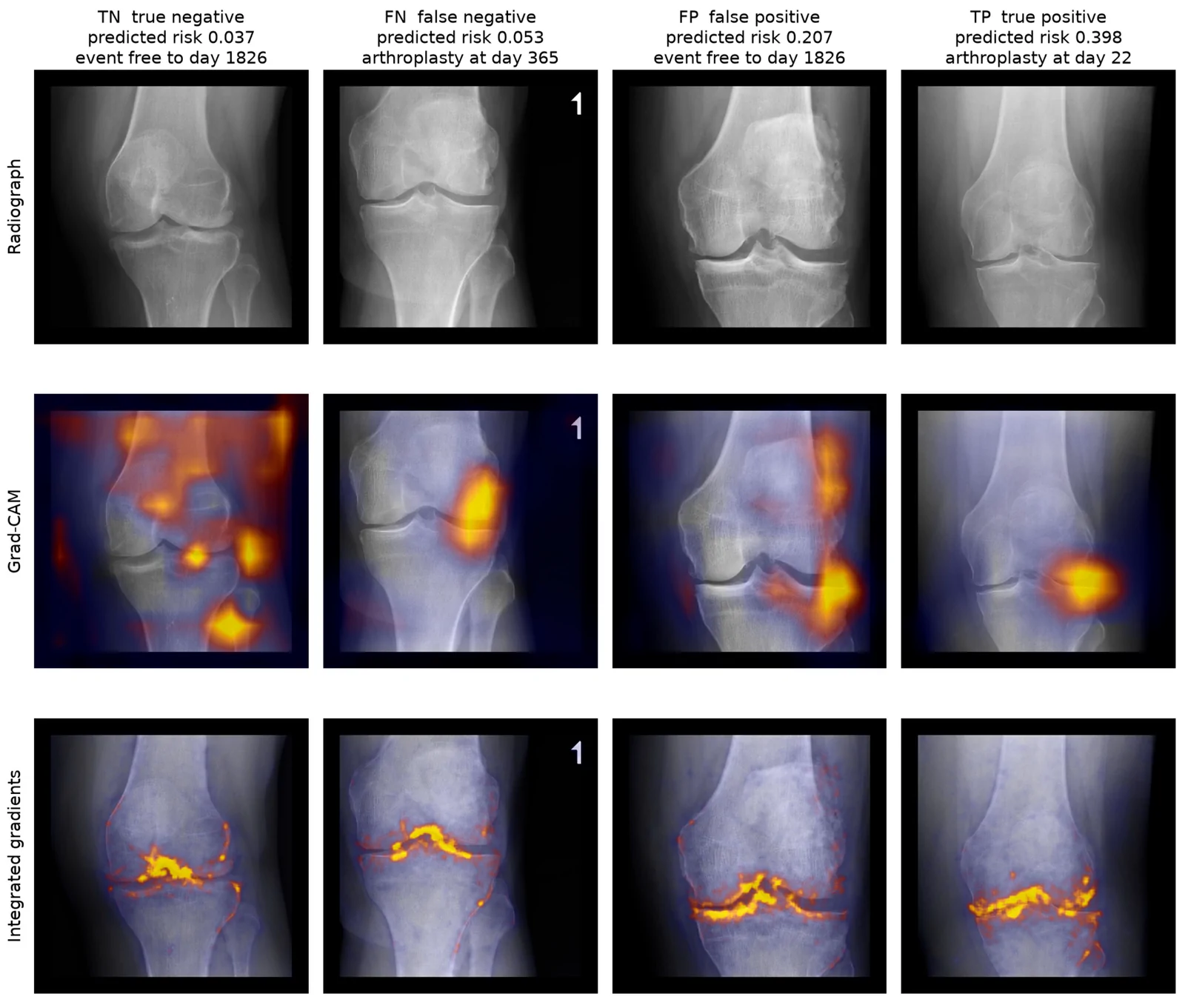
Attribution and occlusion maps for four test-split patients at the same Kellgren–Lawrence grade. Representative image findings at a single radiographic grade. Four test-split patients whose contralateral knee carried an inferred Kellgren–Lawrence grade of 2, one from each cell of the classification table at a fixed operating point of 0.134 predicted 5-year risk. That threshold is the quantile at which the number of predicted positives equals the number of observed events among the 263 patients who are classifiable at 5 years, which is the 106 with the event by then plus the 157 observed event free beyond it; the remaining 471 of 734 are censored earlier and cannot be placed in the table at all and applying the same threshold to the whole test split flags 281. Columns are ordered by predicted risk. (**top row**) is the image the network reads, after the preprocessing in Figure 1. (**middle row**) is the Grad-CAM map at the encoder’s final normalization layer and (**bottom row**) is integrated gradients, both averaged over the five pre-specified seeds and both taken with respect to the predicted 5-year risk. All four risks are shown after the frozen horizon-specific recalibration described in the Methods, which was fitted on the validation split and applied unchanged here, so they are on the scale of every predicted risk this manuscript reports. Predicted 5-year risks across the four columns are 0.037, 0.053, 0.207 and 0.398, a 10.8-fold spread on that recalibrated scale within one radiographic grade, which is the separation the grade itself does not provide; the transform is monotone, so it moves the spread but not the ordering, and the same two patients are 4.24-fold apart on the raw model output. These four are the WIDEST-SPREAD quartet available at this grade: the sampler produced nine candidates at it, and no other choice of one per cell spans a larger ratio, so 10.8-fold is the largest separation these candidates could have been made to show and not a typical one. Each map is scaled to its own 99.5th percentile and drawn with opacity proportional to magnitude, so colour is comparable within a tile and not across tiles; the integrated-gradient tiles are smoothed for display only and every quantity reported in the text is computed on the unsmoothed maps. The false-negative column carries a saturated burned-in numeral “1” at the upper right of the crop, present on the original radiograph rather than added here, clipped by the masked band and visible in all three rows. It was not selected around: the candidate panels come from a seeded sampler that filters only on classification cell and Kellgren–Lawrence grade, and of the three false-negative candidates at this grade the one drawn here is the one carrying the largest residual blob. A total of 18.5 percent of the 1216 finished crops of this split carry such a blob, an upper bound because saturated bone edges share the detector’s signature. Blanking every such blob across the whole split lowers 5-year discrimination by 0.00096 of area under the curve, with a 95 percent interval of 0.00008 to 0.00230; that is the only row of Figure 2’s masked band and burned-in marker block whose interval excludes zero, and it is the bound on what the residual markers can contribute. No claim is made here about where either attribution method placed its mass relative to that character. These are out-of-sample estimates: the test split was read once, after every model and every hyperparameter choice had been frozen.

**Figure 4:**
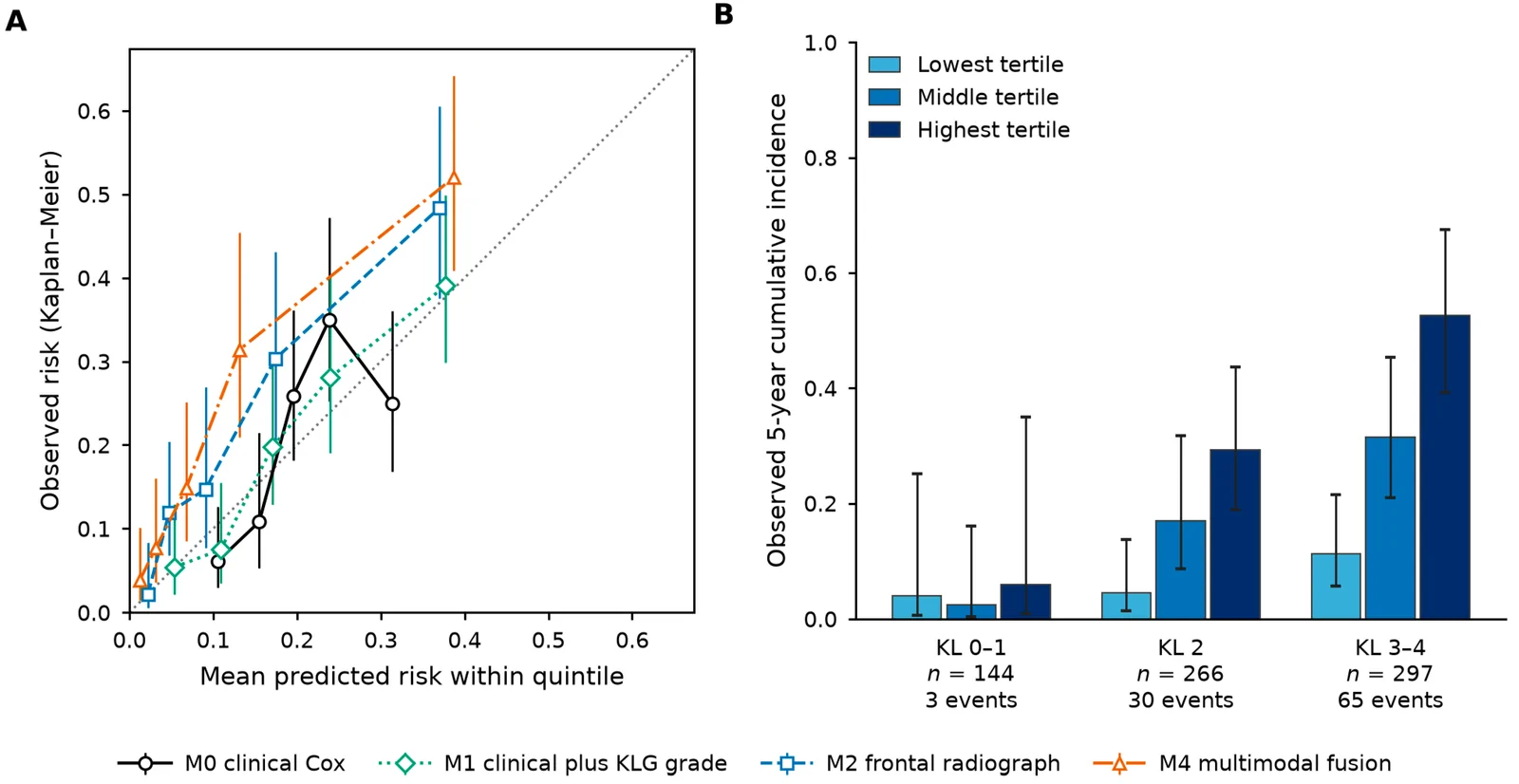
Calibration at 5 years and observed incidence by predicted-risk tertile within Kellgren–Lawrence stratum. (**A**) gives calibration at the 5-year horizon in the test split: each marker is one quintile of predicted risk, plotted as the mean predicted risk against the Kaplan–Meier observed risk within that quintile, vertical bars are Greenwood 95 percent intervals, and the diagonal is the line of perfect calibration. It rests on the 707 patients, carrying 98 events, which all four arms score, so the curves share one population. The two image arms are drawn after the horizon-specific recalibration described in the Methods, which was fitted on the validation split and applied unchanged here, so this panel and the reported calibration slope and calibration in large describe the same predictions. The two frozen Cox comparators carry no such transform and are drawn as fitted. (**B**) gives the observed 5-year Kaplan–Meier incidence of contralateral arthroplasty in each tertile of predicted risk from the frontal-radiograph model, formed within each Kellgren–Lawrence stratum, with Greenwood 95 percent intervals. Stratum sizes and event counts are printed on each group. The lowest stratum carries three events in 144 patients, so its intervals span almost the whole range, and no separation is observable there; that is an uninformative cell rather than a demonstrated null. Death is not ascertainable in this data source, so mortality acts as an unmeasured competing event, and both panels report cause-agnostic risks. The stratified analysis in (**B**) is post hoc on an already-read split and is reported as exploratory; the deviation register carries it. The Kellgren–Lawrence grade is itself model inferred, so ‘beyond the grade’ means beyond an automated grade rather than beyond a radiologist’s reading. These are out-of-sample estimates: the test split was read once, after every model and every hyperparameter choice had been frozen.

**Table 1:** Cohort characteristics of the development and test splits.

Characteristic	Development (*n* = 2968)	Test (*n* = 741)	Missing, %
Age at index, years	66.2 (9.1)	65.7 (9.1)	0.0
Female sex	1859 (62.6)	464 (62.6)	0.0
Radiograph to index interval, days	98 [49 to 223]	90 [50 to 206]	0.0
Any recorded knee pain	2760 (93.0)	681 (91.9)	0.0
Maximum recorded pain score	7 [5 to 9]	7 [5 to 8]	7.2
Obesity	831 (28.0)	216 (29.1)	0.0
Diabetes	534 (18.0)	133 (17.9)	0.0
Hypertension	1594 (53.7)	403 (54.4)	0.0
Autoimmune disease	270 (9.1)	69 (9.3)	0.0
Nicotine use	415 (14.0)	113 (15.2)	0.0
Lower extremity trauma	797 (26.9)	220 (29.7)	0.0
Inferred contralateral Kellgren–Lawrence grade	2 [2 to 3]	2 [2 to 3]	3.9
Index operative side coded on the procedure record	1511 (50.9)	370 (49.9)	0.0
Index operative side recovered by concordance rule	1457 (49.1)	371 (50.1)	0.0
Observed follow-up from landmark, days	832 [275 to 1751]	831 [267 to 1720]	0.0

Development is the combined 70% training and 10% validation split; test is the locked 20% split, held out of model fitting, checkpoint selection and recalibration. Continuous characteristics are mean (SD) or median [interquartile range]; binary characteristics are *n* (%), and percentages within a column are of that column’s patients. The missingness column is the percentage of all 3709 landmark-cohort patients with no recorded value, before imputation. The index operative side was taken as coded where the earliest CPT 27447 record carried a right or left modifier and otherwise recovered by a deterministic concordance rule over three record signals dated on or before the index procedure; that rule was validated against manual adjudication in 200 development patients, where it agreed in 196 (98.0%); that sample held no test patient. In the outcome-record audit, the index side used on the test split, whether coded or recovered, agreed with manual adjudication of the billing record in 101 of 101 patients (Supplementary Results S13).

**Table 2:** Primary and head-to-head model contrasts on the test split.

Contrast	Family	Paired Patients	IPCW AUROC, Model vs. Reference	Difference (95% CI)	*p*, Unadjusted (BH-Adjusted)
M4 combined minus M0 clinical	primary	740	0.844 vs. 0.680	0.164 (0.087 to 0.240)	<0.001 (not adjusted)
M2 frontal minus M0 clinical	post hoc	734	0.837 vs. 0.675	0.162 (0.088 to 0.233)	<0.001 (<0.001)
M2 frontal minus M1 clinical plus grade	post hoc	707	0.846 vs. 0.732	0.114 (0.060 to 0.170)	<0.001 (<0.001)
M3 all views minus M0 clinical	modality	740	0.840 vs. 0.680	0.160 (0.083 to 0.238)	<0.001 (0.001)
M4 combined minus M3 all views	modality	740	0.844 vs. 0.840	0.004 (−0.014 to 0.023)	0.64 (0.64)
M3 all views minus M2 frontal	views	734	0.846 vs. 0.837	0.009 (−0.028 to 0.048)	0.61 (0.61)
M4 combined minus M4 frontal-only combined	views	734	0.849 vs. 0.818	0.031 (−0.005 to 0.071)	0.09 (0.19)
M0d discrete-time clinical minus M0 clinical	modality	740	0.696 vs. 0.680	0.016 (−0.003 to 0.036)	0.10 (0.14)
M4 combined minus M0d discrete-time clinical	modality	740	0.844 vs. 0.696	0.148 (0.070 to 0.225)	<0.001 (0.001)
M2 frontal minus R1 ConvNeXt-Tiny frontal	robustness	734	0.837 vs. 0.709	0.127 (0.076 to 0.176)	<0.001 (<0.001)

Every contrast is the paired difference in inverse probability of censoring weighted (IPCW) time-dependent AUROC at day 1825, computed on the intersection of the two arms it compares, with 95% percentile intervals from a paired patient-level bootstrap of 2000 replicates sharing one resample across arms. An arm’s estimate therefore changes between rows as the paired set changes, and the combined model reads 0.844 on the 740-patient set and 0.849 on the 734-patient set, so a difference need not equal the gap between the marginal levels in Table 3. *p* values at the bootstrap resolution floor of 0.0005 are reported as *p* < 0.001, in the unadjusted and the adjusted column alike. The primary contrast is unadjusted; each pre-declared secondary family is Benjamini–Hochberg adjusted within itself. The two single-view contrasts were not pre-specified, were computed after the locked split had been read, and are reported as post hoc; they form their own adjustment family of two and no published adjusted *p* value changes because of them. The same two contrasts on Harrell concordance give 0.134 (95% CI 0.074 to 0.191; *p* < 0.001) for M2 minus M0 and 0.062 (0.018 to 0.107; *p* = 0.007) for M2 minus M1, and a sensitivity analysis of the second against the discrete-time implementation of the grade model gives 0.055 (0.012 to 0.099) in concordance and 0.105 (0.051 to 0.161) in IPCW AUROC. The contrast between the combined and frontal-view models within advanced radiographic disease is given in Supplementary Table S5.

**Table 3:** Discrimination and calibration of each model arm on the test split.

Model	Patients/Events	IPCW AUROC at 1825 Days (95% CI)	Harrell C (95% CI)	Calibration Slope at 1825 Days	Brier Score at 1825 Days
M0 clinical, frozen penalized Cox	741/106	0.682 (0.617 to 0.744)	0.647 (0.596 to 0.698)	1.28	0.158
M0d clinical, discrete-time control	740/106	0.696 (0.630 to 0.758)	0.658 (0.610 to 0.709)	0.94	0.199
M1 clinical plus inferred KLG, frozen penalized Cox	707/98	0.732 (0.668 to 0.797)	0.724 (0.672 to 0.773)	1.03	0.150
M1 clinical plus inferred KLG, discrete-time control	707/98	0.741 (0.678 to 0.806)	0.732 (0.681 to 0.780)	1.08	0.174
M2 radiograph, frontal view, DenseNet121	734/106	0.837 (0.785 to 0.888)	0.779 (0.738 to 0.817)	1.01	0.143
M3 radiograph, all views, DenseNet121	740/106	0.840 (0.787 to 0.889)	0.780 (0.739 to 0.819)	0.98	0.149
M4 combined, frontal view only	734/106	0.818 (0.764 to 0.870)	0.781 (0.741 to 0.818)	0.98	0.155
M4 combined, all views, primary arm	740/106	0.844 (0.791 to 0.894)	0.786 (0.745 to 0.825)	1.02	0.141
R1 radiograph, frontal view, ConvNeXt-Tiny, robustness	734/106	0.709 (0.643 to 0.775)	0.709 (0.658 to 0.757)	1.03	0.215

All estimates are on the test split; IPCW is inverse probability of censoring weighted. Intervals are 95% percentile intervals from a paired patient-level bootstrap in which a single resample per replicate is shared across arms. Patient counts differ by row and are not harmonized: the grade arms are scored only in patients whose inferred Kellgren–Lawrence grade is observed, and the image arms lose the patients with no admissible crop of the required view, so every paired contrast in Table 2 is computed on the intersection of the two arms it compares. Calibration slope and Brier score for the image arms are computed after the frozen horizon-specific recalibration fitted on the validation split; the two frozen Cox arms are published as fitted and carry no recalibration. The encoder named in each label is the one that arm was trained with, read from the training record: R1 is the robustness arm and retains the originally pre-specified encoder, which a registered protocol deviation replaced everywhere else. Uno concordance, the 365-day and 730-day AUROCs, and calibration-in-the-large at all three horizons are given in Supplementary Table S1.

## Data Availability

The radiographic and linked clinical data analyzed in this study were obtained from the Emory Knee Radiograph (MRKR) dataset, a de-identified collection of 503,261 knee radiographs from 83,011 patients. MRKR is publicly available under the CC BY-SA license through the AWS Registry of Open Data (https://registry.opendata.aws/mrkr/, accessed on 17 July 2026), with access registration at https://data.hitilab.com/ (accessed on 17 July 2026). Documentation of the dataset structure and metadata schema is available at https://github.com/Emory-HITI/MRKR (accessed on 17 July 2026). The dataset is described by Price et al. in the Journal of Imaging Informatics in Medicine (https://doi.org/10.1007/s10278-026-01902-6, accessed on 17 July 2026). The authors do not redistribute the original radiographs or linked clinical records, which remain available from the source repository subject to its applicable license and terms of use. Analysis code supporting cohort construction, outcome and follow-up derivation, clinical variable generation, patient-level data partitioning, image preprocessing, model training, locked test-set evaluation, sensitivity analyses, and generation of aggregate results is publicly available under the MIT License at https://github.com/samersalman/mrkr-contralateral-tka (accessed on 17 July 2026). The version corresponding to the analyses reported in this article is archived on Zenodo (https://doi.org/10.5281/zenodo.22775135, accessed on 17 July 2026) and corresponds to GitHub release v0.2.0. Aggregate outputs supporting the findings, including cohort flow counts and tables of model discrimination, calibration, decision-curve performance, and subgroup analyses, are available in this repository. The trained model weights, which are not part of the MRKR dataset, are available as downloadable assets associated with GitHub release v0.2.0 (https://github.com/samersalman/mrkr-contralateral-tka/releases/tag/v.0.2.0, accessed on 17 July 2026) and are released under CC BY-SA 4.0. These release assets are not included in the associated Zenodo archive. Reproduction of the reported predictions requires independent authorized access to the MRKR source images and compliance with the dataset license and applicable terms of use. Derived patient-level data, including partition assignments and individual predicted risks, are not publicly posted because of data-governance and privacy considerations; the predicted risks for the four de-identified example cases shown in Figure 3 are available in the repository. Other derived patient-level data may be made available by the corresponding author upon reasonable request, subject to applicable data-governance, privacy, and licensing requirements.

## Supplementary Materials

The following supporting information can be downloaded at https://www.mdpi.com/article/10.3390/jimaging12100474/s1, it contains Supplementary Methods S1–S14, which hold the methodological detail, parameter values and protocol-deviation disclosures relocated from the Methods; Supplementary Results S1–S13, which hold the corresponding results detail; Supplementary Tables S1–S10; and Supplementary Figures S1–S6; Supplementary Methods S1–S8 and Supplementary Results S1–S9 follow the section order of the Methods and Results of this article, so any statement in the main text can be resolved against them; Supplementary Methods S9–S14 and Supplementary Results S10–S13 hold the analyses added at revision. Completed CLAIM 2024 and TRIPOD+AI checklists and the deviation register accompany the submission as separate files.
