# Supplementary material for: Deep Learning Analysis of Routine Preoperative Knee Radiographs for Five-Year Prediction of Contralateral Arthroplasty
Source: J Imaging. 2026 Sep 30;12(10):474. doi: 10.3390/jimaging12100474 (PMC13705656; doi:10.3390/jimaging12100474)
Supplement: Supplementary file 1 [file jimaging-12-00474-s001.zip › CLAIM_Checklist_completed_v6.pdf]

Checklist for Artificial Intelligence in Medical Imaging (CLAIM): 2024 Update

Completed for: Single-View Deep Learning Analysis of Routine Knee Radiographs for Five-Year Prediction of Contralateral Arthroplasty (v6 resubmission to Journal of Imaging)

Completion summary: 41 Yes, 2 No, 1 Not Applicable, over 44 rows. Counts re-derived on 2026-08-13 from this file's own status cells rather than adjusted by arithmetic. Re-audited on 2026-08-12 against the two-document v6 submission after the manuscript body was cut to about 3,500 words and the relocated detail was moved into a companion supplementary file. One status moved on 2026-08-13: item 18 goes No to Yes. Items 13 and 43 remain No and item 34 remains Not Applicable. Item 18 moved because the protocol section-23 two-reviewer image review, which the row named as its route to Yes, was performed on 2026-08-13 and reports raw agreement of 95% to 97.9% and Cohen kappa of 0.81 to 0.89 across its six items (deviation D40, which withdraws the decline it registered on 2026-08-12). TRIPOD+AI item 7 does NOT move with it: that item also asks whether pre-processing quality is similar across sociodemographic groups, which this review does not address, so it stays No on that component alone. Yes means every component of the item is addressed somewhere in the submission; No means at least one component is not addressed or was not performed, and the entry names which; Not Applicable means the item's precondition does not hold for this study design. Locators name a document and a heading, never a page or line number, because page numbers depend on the typeset proof and a wrong page number is worse than none. Manuscript means [c] v6 MRKR_contralateral_TKA.docx and Supplementary Materials means [supp] v6 supplementary-material.docx; both are part of this submission. Within the supplement, Supplementary Methods S1 to S8 carry the same eight headings as the Methods and Supplementary Results S1 to S9 follow the order of the Results, so any statement in the body resolves against them; where a Supplementary Methods paragraph is named, the name given is that paragraph's bold lead-in. The eight Methods subsections are named exactly as the manuscript heads them and are unchanged from the previous draft; all eight Results subsection headings changed in the cut and are named as the manuscript now heads them. The three tables numbered 4, 5 and 6 in the previous version are now Supplementary Tables S3, S4 and S5 respectively and are cited under those names throughout; the manuscript itself now carries only Tables 1 to 3 and Figures 1 to 4, so no locator in this file names a Table above 3. Item 43 stays No: the three modules that produced this revision's analyses were pushed to the public GitHub repository at commit b568820 on 2026-08-11, but no new archived Zenodo version has been minted, the Code Availability Statement still cites the DOI covering the pre-revision code, and the manuscript still nowhere states whether the trained model weights are available. TRIPOD+AI items 18f and 22 are scored No on the same evidence and the two checklists still agree.

Section / Topic	No.	Checklist item	Yes	No	NA	Location in the submission and rationale
**TITLE/ABSTRACT**	1	Identification as a study of AI methodology, specifying the category of technology used (eg, deep learning)	X			Manuscript, Title page, title; Manuscript, Abstract, Background and Methods sentences. The title identifies single-view deep learning analysis of knee radiographs; the abstract specifies the multi-view DenseNet121 survival network and the frontal-view arm.
**ABSTRACT**	2	Summary of study design, methods, results, and conclusions	X			Manuscript, Abstract, in full (Background through Conclusions). The structured abstract reports the data source, the locked test cohort and its event count, the model arms compared, the evaluation metric, the discrimination, attribution and view results, and the clinical conclusion. The leakage controls are not in the abstract; they are in Manuscript, Results, Where the model looks, and what it is not reading, in Manuscript, Discussion, second paragraph, and in Supplementary Materials, Supplementary Table S3 Panel B.
**INTRODUCTION**	3	Scientific and/or clinical background, including the intended use and role of the AI approach	X			Manuscript, Introduction, all five paragraphs, including the three imaging questions in the fourth paragraph. The clinical problem, current radiographic grading practice, the literature gap, the intended prognostic role and the anticipated use are described. The revision frames the gap as three imaging questions: whether a routine radiograph carries prognostic information beyond conventional grading, whether additional views are necessary, and whether the signal is robust to real-world acquisition variability.

Section / Topic	No.	Checklist item	Yes	No	NA	Location in the submission and rationale
**INTRODUCTION**	**4**	Study aims, objectives, and hypotheses	**X**			Manuscript, Introduction, fourth and fifth paragraphs (the three imaging questions and the objectives); Manuscript, Methods, Image interpretability and leakage controls, first paragraph (deviations D35 and D36); Manuscript, Methods, Imaging robustness and ablation analyses (deviation D35); Supplementary Materials, Supplementary Methods S6, Status (deviations D35 and D36); Supplementary Materials, Supplementary Methods S7, Strata (deviation D35). The pre-specified objective is to predict 5-year contralateral TKA and to compare image, multiview and clinical-input strategies. The single-view contrasts, the Kellgren-Lawrence-stratified analyses, the interpretability and leakage package and the expanded imaging robustness strata added at revision are labelled post hoc and exploratory (deviations D35 and D36).
**METHODS - Study Design**	**5**	Prospective or retrospective study	**X**			Manuscript, Methods, Radiographic dataset and image selection, first paragraph; Manuscript, Title page, Institutional Review Board Statement. The work is identified as a retrospective secondary analysis of a de-identified, single-institution dataset.
**METHODS - Study Design**	**6**	Study goal	**X**			Manuscript, Introduction, fifth paragraph; Manuscript, Methods, Image interpretability and leakage controls, first paragraph (the protocol section 22 package, deviation D36); Manuscript, Discussion, fourth paragraph, which opens These findings support stratification, not quantification; Supplementary Materials, Supplementary Methods S6, Status (deviations D35 and D36). The study is a prognostic model-development and internal-testing study intended to support individualized counselling and planning. The revision adds the protocol section-22 explainability and shortcut-testing aim, which was pre-specified but never executed before this revision (deviation D36).
**METHODS - Data**	**7**	Data sources	**X**			Manuscript, Title page, Data Availability Statement; Manuscript, Methods, Radiographic dataset and image selection, first paragraph; Supplementary Materials, Supplementary Methods S1, Eligibility and Image selection. The MRKR source dataset, institution, linked records, size, public access route, license and documentation are reported.
**METHODS - Data**	**8**	Inclusion and exclusion criteria	**X**			Manuscript, Methods, Radiographic dataset and image selection, first paragraph; Supplementary Materials, Supplementary Methods S1, Eligibility and Image selection; Supplementary Materials, Supplementary Figure S2 (cohort flow). Eligibility, age, index procedure, laterality, image window, exclusions, follow-up and cohort flow are described. The body states the eligibility rule in one sentence; the full criteria, including the infection and osteomyelitis exclusion, the admitted and dropped view codes and the one-to-five crops per patient, are in Supplementary Methods S1.

Section / Topic	No.	Checklist item	Yes	No	NA	Location in the submission and rationale
**METHODS - Data**	**9**	Data preprocessing	**X**			Manuscript, Methods, Image preprocessing; Manuscript, Figure 1 caption; Manuscript, Methods, Image-quality and laterality verification, second paragraph (the deviation D39 disclosure); Manuscript, Results, Manual review and burned-in markers; Supplementary Materials, Supplementary Methods S3, Decoding and geometry, Joint localization, Border masking, Marker masking (deviation D39) and Augmentation and the masking gate; Supplementary Materials, Supplementary Results S1 and S6. Horizontal-flip correction, contralateral half-selection, joint localization, square cropping, 512 by 512 Lanczos resampling, 8-bit greyscale conversion, 31-pixel border-band masking (22.75% of image area), burned-in marker masking, mirroring to a common side and capped augmentation are specified, with the images removed by the section-13 gates reconciled against the manifest: 6,090 selected images to 6,071 written crops, 19 excluded, 17 in development and 2 in the test split (Supplementary Methods S3, Augmentation and the masking gate). The revision additionally discloses that the masked band is not exactly zero on 145 of 1,216 test crops (11.9%), a rate that is 6.8% among the 858 frontal crops (Supplementary Results S6), because marker masking runs after border masking and refills each accepted blob with that blob's own surrounding ring median, reaching a maximum of 48 of 255 across 0.049% of band pixels. Re-zeroing that residue moves discrimination by -0.0002 (95% CI -0.0009 to 0.0000), which is the direct measurement of what it is worth, and the violation is permanent in the frozen crops (deviation D39).
**METHODS - Data**	**10**	Selection of data subsets	**X**			Manuscript, Methods, Radiographic dataset and image selection, first paragraph (the fixed view preference order); Manuscript, Methods, Image preprocessing (the bilateral half-selection rule); Supplementary Materials, Supplementary Methods S1, Image selection and Crop counts by view; Supplementary Materials, Supplementary Methods S3, Augmentation and the masking gate; Supplementary Materials, Supplementary Results S1. The submission explains contralateral-knee cropping, eligible view selection, the bilateral half-select rule and the number of crops contributed per patient, which is one to five. Half-selection was applied to 4,069 of 6,071 finished crops; the 19 images dropped by the excessive-masking and view-drift gates reconcile the 6,090-image manifest to the 6,071 finished crops.
**METHODS - Data**	**11**	De-identification methods	**X**			Manuscript, Title page, Institutional Review Board Statement and Data Availability Statement; Manuscript, Methods, Radiographic dataset and image selection, first paragraph (the de-identified single-institution archive); Manuscript, Results, Robustness strata (no acquisition tag was released and the study date carries an unconfirmed per-patient shift, deviation D17); Supplementary Materials, Supplementary Methods S1, Acquisition metadata; Supplementary Materials, Supplementary Methods S7, Acquisition era (deviation D17); Supplementary Materials, Supplementary Table S2, Strata that could not be constructed at all. The dataset is identified as de-identified and publicly available; the IRB record and the absence of identifiable private information are reported. De-identification also removed every DICOM equipment, manufacturer and institution tag, and study dates carry a per-patient random shift, so absolute calendar dates are not comparable across patients (see item 13 and deviation D17).

Section / Topic	No.	Checklist item	Yes	No	NA	Location in the submission and rationale
**METHODS - Data**	**12**	How missing data were handled	**X**			Manuscript, Table 1 and its note (the missingness column, stated as the percentage with no recorded value before imputation); Manuscript, Results, Prediction within Kellgren-Lawrence strata (the grade recorded for 707 test patients carrying 98 events); Supplementary Materials, Supplementary Methods S8, Comparators (deviations D20, D21, D23 and D24), which states that missing values took a training-split median or mode, and Prediction within radiographic severity, which states that the grade is missing for 34 of the 741 test patients; Supplementary Materials, Supplementary Results S1 (7.0% and 3.7% development missingness against the 7.2% and 3.9% landmark-cohort figures Table 1 reports). Clinical missingness was imputed with training-split medians or modes, and variable-specific missingness is reported, including the 34 of 741 test patients with no recorded contralateral Kellgren-Lawrence grade, who are excluded from every KLG-conditioned analysis. After the cut the imputation rule itself is stated in the Supplementary Methods rather than in the body, which retains only the phrase before imputation in the Table 1 note.

Section / Topic	No.	Checklist item	Yes	No	NA	Location in the submission and rationale
**METHODS - Data**	**13**	Image acquisition protocol	**X**			Supplementary Materials, Supplementary Methods S1, Acquisition metadata, which opens No acquisition parameter accompanies these images; Manuscript, Results, Robustness strata; Manuscript, Discussion, fifth paragraph; Supplementary Materials, Supplementary Table S2, Strata that could not be constructed at all. The locator points at the disclosure of the gap, not at a description of a protocol that does not exist. No. The source dataset released no image-acquisition parameters. MRKR delivered no Manufacturer, ManufacturerModelName, DeviceSerialNumber, StationName, InstitutionName or exposure tags in any table; a case-insensitive search of the delivered image table (derived-data/source-parquet/image.parquet) and of the identical metadata-files/MRKR_image_metadata.csv for manufacturer, model, serial number, station, institution, kVp, exposure, tube current and slice thickness returns no matching column at all, so the acquisition-parameter class is empty rather than sparse (outputs/tables/v6_robustness_metadata_availability.csv). What was released is wider than the acquisition-parameter gap suggests, and this row does not understate it. Those two files carry 19 identical columns: four de-identified identifiers, a randomly shifted StudyDate_anon, age_at_exam, StudyDescription, SeriesDescription, dicom_path, the stored pixel dimensions img_height and img_width, and the model-inferred annotations laterality, view_position, horizontal_flip, weight_bearing, inverted, arthroplasty, L_KLG_inference and R_KLG_inference. Three statements made elsewhere in this package rest on fields in that list: L_KLG_inference and R_KLG_inference are the source of the contralateral Kellgren-Lawrence grade that the M1 comparator uses as a predictor; that same grade defines the strata of the within-grade risk-tertile analysis; and weight_bearing and inverted are robustness strata reported under item 30 and drawn in Supplementary Figure S5. Not one of the 19 columns records how the radiograph was acquired, which is what this item asks for. The 34.85 GB source DICOM set is no longer held and the retained artifacts are 512 by 512 crops with the border band zeroed, so the tags cannot be recovered. The submission reports the radiographic views and the image characteristics that do exist and states the gap as a limitation; it does not describe the acquisition protocol and does not claim to have characterized variation in equipment or exposure. This item cannot be met on this dataset by any amount of further work.
**METHODS - Reference Standard**	**14**	Definition of method(s) used to obtain reference standard	**X**			Manuscript, Methods, Radiographic dataset and image selection, first paragraph (CPT 27447 within 5 years of the day-90 landmark, censored at day 1826); Supplementary Materials, Supplementary Methods S1, Eligibility. The outcome is contralateral TKA identified from linked CPT 27447 records within 5 years after the day-90 landmark.
**METHODS - Reference Standard**	**15**	Rationale for choosing the reference standard	**X**			Manuscript, Introduction, first and second paragraphs; Manuscript, Methods, Radiographic dataset and image selection, first paragraph. Contralateral arthroplasty is the clinically relevant definitive event, selected instead of radiographic progression or a subjective severity label.

Section / Topic	No.	Checklist item	Yes	No	NA	Location in the submission and rationale
**METHODS - Reference Standard**	**16**	Source of reference standard annotations	**X**			Manuscript, Title page, Data Availability Statement; Manuscript, Methods, Radiographic dataset and image selection, first paragraph; Supplementary Materials, Supplementary Methods S1, Eligibility. Reference outcomes originate from the MRKR dataset linked to procedural records rather than from new human image annotations.
**METHODS - Reference Standard**	**17**	Annotation of test set	**X**			Manuscript, Methods, Radiographic dataset and image selection, second paragraph (the locked split took no part in fitting or checkpoint selection and was scored exactly once); Manuscript, Methods, Image-quality and laterality verification, second paragraph (the three manual reviews and the state of each, deviations D22 and D40); Manuscript, Results, Manual review and burned-in markers (the section 23 review result); Supplementary Materials, Supplementary Methods S1, Splitting; Supplementary Materials, Supplementary Methods S2, Section 23 manual image review (deviation D40). The same record-derived outcome algorithm was applied to every partition before splitting; the locked test set was held unread and scored exactly once after every model was frozen, under a gate that records that single read (deviation D31 in the accompanying deviation register). No human image annotation was used to label the test set. The protocol section-23 manual review of a 400-image sample that included 82 test crops was performed by two independent reviewers on 2026-08-13, so images of the reported split were inspected; the selected half remains undecidable on those 82 crop-only panels by construction (deviation D40, and see item 18).

Section / Topic	No.	Checklist item	Yes	No	NA	Location in the submission and rationale
**METHODS - Reference Standard**	**18**	Measures of inter- and intrarater variability of features described by the annotators	X			Manuscript, Methods, Image-quality and laterality verification, first paragraph (the model-inferred MRKR annotations and the uncheckable horizontal-flip flag) and second paragraph (the three manual reviews and the state of each, deviations D22 and D40); Manuscript, Results, Manual review and burned-in markers (the section 23 review result and the section 7 audit result); Manuscript, Discussion, fifth paragraph; Supplementary Materials, Supplementary Methods S2, Section 23 manual image review (deviation D40), Section 7 laterality audit (deviation D22) and Dataset-supplied image annotations. Yes, as of 2026-08-13. The record-derived outcome requires no human annotation, so no rater variability applies to it. The annotator-dependent measurement this item bears on is the image-level quality annotation, and it now carries an inter-rater estimate. Protocol section 23 pre-specifies two independent reviewers with orthopaedic or musculoskeletal imaging experience, raw agreement, Cohen kappa, consensus or third-reviewer adjudication and a 2% critical-error gate over a stratified sample of at least 400 index images. That review was PERFORMED on 2026-08-13 (deviation D40). Two reviewers with musculoskeletal imaging experience scored the 400-image sample (train 273 / val 45 / test 82, 393 patients) independently and their disagreements were adjudicated in a separate recorded step. Across the six items raw agreement runs 95% to 97.9% and Cohen kappa 0.81 to 0.89; the critical-error rate runs 0.5% to 1.0% against the pre-specified 2% threshold, which it clears, so no expansion was triggered. Per item, laterality, view, native-knee status and freedom from non-knee content were each correct on at least 99%, crop adequacy on at least 98%, and at least 95% of crops were clean of burned-in text; these are the bounds and ranges the reviewers' summary reports and none is converted to a point estimate. Three qualifications are stated rather than left to inference. First, laterality was assessable on 318 of the 400 images, the panels that carry a full film; the 82 test-split panels show the finished crop alone, on which the selected half is undecidable by construction, so that figure must never be attributed to 400 images. Second, intra-rater variability is not measured: each reviewer read each image once, so the design yields an inter-rater estimate and no repeat-read estimate, and the submission says so. Third, the per-image adjudications are held on a secure institutional system and are not part of the release, so the released material is the summary statistics rather than the per-image scores. Separately, the contralateral Kellgren-Lawrence grade used by the M1 comparator is dataset-inferred by an automated model rather than read by a human, so it is not a feature described by an annotator and no interrater estimate applies to it. The protocol section 7 laterality audit is also complete and is reported alongside: the deterministic index-side recovery rule agreed with manual adjudication on 196 of 200 sampled development patients, 98.0% (Wilson 95% CI 95.0 to 99.2), a 2.0% observed error rate (deviation D22). That audit does not by itself satisfy this item, because it compares a rule against a reviewer rather than two raters against each other; the section 23 review does. The section 23(ii) outcome-record review of 200 records is drawn and unadjudicated (deviation D22), so it reports no agreement statistic, and it is reported under item 40 as an outstanding review rather than here.

Section / Topic	No.	Checklist item	Yes	No	NA	Location in the submission and rationale
**METHODS - Data Partitions**	**19**	How data were assigned to partitions	**X**			Manuscript, Methods, Radiographic dataset and image selection, second paragraph (the locked, stratified 70% training, 10% validation and 20% test partition); Supplementary Materials, Supplementary Methods S1, Splitting, which names the stratification variables as 5-year event status, sex and major race group. Patients were assigned once to locked 70% training, 10% model-optimization and 20% test partitions with the stratification factors reported. After the cut the body states that the partition was stratified and the Supplementary Methods name the three variables.
**METHODS - Data Partitions**	**20**	Level at which partitions are disjoint	**X**			Manuscript, Methods, Radiographic dataset and image selection, second paragraph, in which the 3,709 qualifying patients were partitioned once, that is at the patient level; Supplementary Materials, Supplementary Methods S1, Splitting (Patients were partitioned once). Partitioning was performed at the patient level, preventing a patient's images from appearing in more than one partition.
**METHODS - Testing Data**	**21**	Intended sample size	**X**			Manuscript, Methods, Clinical comparators and statistical analysis, final paragraph (the sample checked against the Riley criteria); Manuscript, Results, Prediction within Kellgren-Lawrence strata (the 98-event arithmetic); Supplementary Materials, Supplementary Methods S8, Prediction within radiographic severity. The locked test set size and event count are specified, and the sample was checked against Riley prediction-model criteria. The revision also states the arithmetic limit the same sample imposes on stratified analysis: the 98 KLG-eligible events cannot support two strata each clearing the pre-specified 50-event floor, which would need at least 100, so at most one within-grade stratum is estimable under any partition and the one that clears is necessarily the advanced-disease stratum.
**METHODS - Model**	**22**	Detailed description of model	**X**			Manuscript, Methods, Network architecture and multi-view aggregation, both paragraphs; Manuscript, Table 3; Supplementary Materials, Supplementary Methods S4, Architecture, The nine arms and Encoder substitution (deviation D28); Supplementary Materials, Supplementary Table S1. The DenseNet121 encoder, the learned 32-dimensional view embedding, masked attention pooling, clinical fusion, ten six-month interval hazards, censoring treatment and the nine model arms are described; the body gives the architecture and points to Table 3, and Supplementary Methods S4 defines the nine arms one by one. The nine are M0 and M1, the two frozen ridge-penalized Cox comparators (artifact keys m0 and m1), their discrete-time controls M0d and a discrete-time M1 (m0d_clinical and m1_klg), the frontal-view image model M2, the all-views image model M3, the multimodal fusion arms M4-frontal and M4, and the ConvNeXt-Tiny robustness arm R1; Table 3 and Supplementary Table S1 report all nine, and outputs/tables/test_metrics.csv carries all nine. Seven of the nine are trained networks, so outputs/tables/train_history.csv and Supplementary Figure S1 carry seven: M0 and M1 are replayed from stored coefficients and never enter the training ladder. Note for anyone reading the released tables that the robustness arm keyed r1_densenet_frontal is a ConvNeXt-Tiny network; the arm key is a legacy misnomer, the submission labels that arm ConvNeXt-Tiny in every table and in the text, and the architecture label in train_arms.json is authoritative.

Section / Topic	No.	Checklist item	Yes	No	NA	Location in the submission and rationale
**METHODS - Model**	**23**	Software libraries, frameworks, and packages	**X**			Manuscript, Methods, Training and internal validation (the sealed-read artifact records no interpreter, device or precision and the published environment file describes the revision workstation, deviations D37 and D38); Supplementary Materials, Supplementary Methods S5, Environment (deviations D37 and D38), which names the cloud graphics processor, float16 automatic mixed precision, Python 3.12.13, PyTorch 2.11.0 and timm 1.0.28. The framework and architectures are named with versions taken from the record of the environment that actually produced the models, derived-data/cohort/train_arms.json interpreter block: Python 3.12.13, Linux-6.6.122+-x86_64-with-glibc2.35, torch 2.11.0+cu128, timm 1.0.28, numpy 2.0.2, pandas 2.2.2, device cuda, automatic mixed precision enabled. outputs/environment_training.json describes the local macOS analysis and re-run environment only and is not the training environment (deviation D38); the frozen sealed-read artifact records no environment at all (deviation D37). The code that trains the models and scores the sealed split (src/train_model.py, src/score_test.py) is in the public repository the manuscript cites. The modules that produced this revision's interpretability, single-view and within-grade analyses were added to that repository at commit b568820 on 2026-08-11, together with the aggregate v6 output tables, but they are still absent from the archived Zenodo version the Code Availability Statement cites, which is frozen at the pre-revision commit dated 2026-07-30. The restriction is recorded under item 43 and under TRIPOD+AI item 18f, and no claim is made here that the archived code covers this revision's analyses.
**METHODS - Model**	**24**	Initialization of model parameters	**X**			Manuscript, Methods, Network architecture and multi-view aggregation, first paragraph (the ImageNet-initialized DenseNet121 encoder); Manuscript, Methods, Training and internal validation; Manuscript, Title page, Code Availability Statement; Supplementary Materials, Supplementary Methods S4, Architecture; Supplementary Materials, Supplementary Methods S5, Optimization (deviation D26), which states that the encoder was frozen for the first three epochs and the head initialized at 0.1 of its normal scale. Initialization, including ImageNet-pretrained encoder weights and the architecture-specific implementation details, is provided in the Supplementary Methods and in the reproducible code the manuscript references.
**METHODS - Training**	**25**	Details of training approach	**X**			Manuscript, Methods, Training and internal validation; Supplementary Materials, Supplementary Methods S5, Optimization (deviation D26), Recalibration and Convergence; Supplementary Materials, Supplementary Methods S3, Augmentation and the masking gate; Supplementary Materials, Supplementary Results S5; Supplementary Materials, Supplementary Figure S1. Augmentation limits, optimizer, learning-rate schedule, warm-up, gradient clipping, early stopping on validation negative log-likelihood, the objective, the five pre-specified seeds, hazard-averaged ensembling and the frozen validation-fitted recalibration are described, together with dropout 0.3 and AdamW weight decay 0.05. After the cut the body keeps early stopping, the five seeds, the hazard averaging and the frozen recalibration, and the parameter values are in Supplementary Methods S5 and S3. Supplementary Figure S1 shows per-arm, per-seed learning curves; at the retained checkpoint the validation-minus-training negative log-likelihood gap was no larger than 0.052 for any arm, and those gaps are lower bounds.

Section / Topic	No.	Checklist item	Yes	No	NA	Location in the submission and rationale
**METHODS - Training**	**26**	Method of selecting the final model	**X**			Manuscript, Methods, Training and internal validation (early stopping on validation loss; the test split entered neither checkpoint selection nor recalibration); Supplementary Materials, Supplementary Methods S5, Optimization (deviation D26), which states that the retained checkpoint is the epoch at the validation negative-log-likelihood minimum, and Convergence. Checkpoint selection used validation negative log-likelihood; the test set took no part in model or hyperparameter selection.
**METHODS - Training**	**27**	Ensembling techniques	**X**			Manuscript, Methods, Training and internal validation (five pre-specified seeds averaged on the hazards); Supplementary Materials, Supplementary Methods S5, Optimization (deviation D26). Five pre-specified seeds per arm were ensembled by averaging predicted interval hazards and converting to risk once.
**METHODS - Evaluation**	**28**	Metrics of model performance	**X**			Manuscript, Methods, Clinical comparators and statistical analysis, second and third paragraphs; Manuscript, Methods, Image interpretability and leakage controls, second paragraph; Supplementary Materials, Supplementary Methods S8, Estimand and uncertainty and Prediction within radiographic severity; Supplementary Materials, Supplementary Methods S6, Attribution, Regions, Occlusion and negative controls, and View attention and view withholding. Censoring-weighted time-dependent (IPCW cumulative/dynamic) AUROC, Harrell and Uno concordance, calibration slope and calibration-in-the-large, Brier score, decision curves and the comparator models are specified; the body names the primary estimand and the Supplementary Methods carry the full metric list and the day-1825-rather-than-1826 rationale. The revision adds Cox hazard ratios per standard deviation of the image risk score with likelihood-ratio tests against a model already containing the Kellgren-Lawrence grade, observed Kaplan-Meier 5-year incidence by risk tertile within grade with log-rank tests, attribution mass fractions by image region with area-normalised enrichment, change in AUROC and in mean predicted 5-year risk under occlusion, and per-view attention weights.
**METHODS - Evaluation**	**29**	Statistical measures of significance and uncertainty	**X**			Manuscript, Methods, Clinical comparators and statistical analysis, second paragraph (the paired 2,000-replicate bootstrap, the unadjusted primary contrast, Benjamini-Hochberg adjustment within each secondary family and the 50-event suppression rule); Manuscript, Table 2 note (P values at the bootstrap resolution floor of 0.0005 are reported as P < .001); Supplementary Materials, Supplementary Methods S8, Estimand and uncertainty and Multiplicity and suppression. Paired patient-level bootstrap confidence intervals on 2,000 replicates sharing one resample across arms, P values, Benjamini-Hochberg adjustment within pre-declared families and the 50-event suppression rule are reported. The post hoc contrasts added at revision form their own adjustment families (posthoc_single_view m=2, posthoc_single_view_c m=2, posthoc_single_view_sens m=2, posthoc_kl_within 2 estimable of 6) and are adjusted inside those alone, so no published adjusted P value moved.

Section / Topic	No.	Checklist item	Yes	No	NA	Location in the submission and rationale
**METHODS - Evaluation**	**30**	Robustness or sensitivity analysis	**X**			Manuscript, Methods, Imaging robustness and ablation analyses; Manuscript, Results, Robustness strata; Manuscript, Methods, Network architecture and multi-view aggregation, second paragraph (deviation D28 and protocol section 25); Supplementary Materials, Supplementary Methods S7, Strata (deviation D35), Suppression and undefined cells, Two masking definitions, Acquisition era (deviation D17) and Two view estimands; Supplementary Materials, Supplementary Results S7; Supplementary Materials, Supplementary Table S2, including its Strata that could not be constructed at all panel; Supplementary Materials, Supplementary Figure S5. Robustness analyses cover an alternative encoder, multiple views, multimodal inputs, five seeds per arm and pre-specified subgroups. The revision adds imaging robustness strata for weight-bearing status, view availability, acquisition era under three declared binning schemes, image quality (masked fraction, crop confidence, localization method), laterality source, photometric inversion and bilateral half-selection, plus occlusion, fill-value and region-definition sensitivity analyses in the interpretability package and a tie-rule sensitivity for the Kellgren-Lawrence bin edges. The limits are reported rather than glossed: across the two image arms the stratum table holds 136 cells, of which 62 carry an estimate and 74 do not, 68 suppressed by the pre-specified 50-event floor and 6 because no patient in the stratum was observed beyond the 5-year horizon, which makes the estimator undefined rather than imprecise (Supplementary Results S7). Only the masking family has two estimable levels, so the remaining families compare one stratum against the whole split. Acquisition era must not be read as an equipment or technology effect: de-identified study dates carry a per-patient random shift whose cross-patient comparability protocol section 17 requires in writing and which has never been obtained (deviation D17), and era is confounded with follow-up length and record availability. Equipment, manufacturer and site strata are impossible on this dataset and the reason is reported (item 13). Protocol section 25 specifies a DenseNet121 robustness architecture; because DenseNet121 became the primary encoder (deviation D28), what was run meets that item's intent and not its wording.

Section / Topic	No.	Checklist item	Yes	No	NA	Location in the submission and rationale
**METHODS - Evaluation**	**31**	Methods for explainability or interpretability	**X**			Manuscript, Methods, Image interpretability and leakage controls, both paragraphs; Manuscript, Results, Where the model looks, and what it is not reading, in full; Manuscript, Figure 2 and Figure 3 with their captions; Supplementary Materials, Supplementary Methods S6, Status (deviations D35 and D36), Attribution, Regions, Anatomic registration of the region, Occlusion and negative controls, Randomization checks, View attention and view withholding, and Provenance of every reported difference; Supplementary Materials, Supplementary Results S2 and S6; Supplementary Materials, Supplementary Table S3 Panels A and B; Supplementary Materials, Supplementary Table S4 Panel A; Supplementary Materials, Supplementary Figure S6. Yes. Post hoc attribution was computed for the frontal-view model (M2) on the locked test split with two methods: Grad-CAM at the DenseNet121 encoder's final normalisation layer (encoder.features.norm5, native 16 by 16 resolution) and integrated gradients (32 midpoint steps, zero baseline, pixel resolution). Both were averaged over the five pre-specified seeds and target the model's predicted 5-year risk. Attribution was quantified, not only displayed: the fraction of absolute attribution mass falling inside a predefined tibiofemoral joint region (the central half of the 512-pixel crop, 25.00% of the image), inside the 31-pixel masked border band (22.75%), and in the remaining background. Grad-CAM was computed on all 858 frontal test crops from 734 patients and integrated gradients on 230 crops from 200 patients; the two denominators are not interchangeable. Grad-CAM placed a mean 37.8% (SD 13.1) of its mass in the joint region, a 1.51-fold enrichment over that region's area, and 8.4% (SD 4.7) in the border band; integrated gradients placed 55.8% (SD 11.6) in the joint region and essentially no mass in the border band (mean 5.6 x 10^-6, SD 3.7 x 10^-5, maximum 4.9 x 10^-4, non-zero on 18 of 230 crops), which is zero by construction only where the band pixel is exactly zero, so the cohort-level value is essentially rather than exactly zero. Representative maps were generated for a Kellgren-Lawrence-matched true-positive / false-positive / true-negative / false-negative sample of 32 patients, 8 per cell; Figure 2 reproduces one patient from each of the four cells, all at grade 2, and the other 28 panels are not part of the submission (see item 39). Leakage was tested by occlusion: blanking every residual saturated marker-like blob across the whole test set changed discrimination by -0.00096 (95% CI -0.00230 to -0.00008), and widening the masked band from 31 to 62 and to 93 pixels changed it by -0.0038 (95% CI -0.0180 to +0.0115) and -0.0154 (95% CI -0.0414 to +0.0102); all three are upper bounds because they also remove peri-articular anatomy and saturated bone edges, and they bound any contribution from burned-in text at roughly one thousandth of AUROC. Negative controls (a joint-occluded input, a joint-only crop and the pre-specified border-band-only input) were re-scored through the frozen five-seed ensemble against a locally recomputed unperturbed baseline. The border-band-only control is degenerate on these data rather than reassuring, because the pipeline already zeroes that band, so 676 of 734 patients receive one identical risk, only 59 distinct risks exist across the split, and its chance-level discrimination (AUROC 0.497) follows by construction rather than from the absence of text; the leakage conclusion rests on the marker-blanking and band-widening results instead, and re-zeroing the already-blank band (677 of 734 patients bit-identical, delta -0.0002) is reported as a pipeline validation and not as a leakage test. The attribution methods were validated in this study by cascading randomisation of the model parameters, after which the map correlation with the trained network fell from +0.604 for the untouched seed to at or below +0.268 in every randomisation scope, three of five at or below zero

Section / Topic	No.	Checklist item	Yes	No	NA	Location in the submission and rationale
**METHODS - Evaluation**	**32**	Evaluation on internal data	**X**			Manuscript, Methods, Radiographic dataset and image selection, second paragraph (the single sealed read); Manuscript, Methods, Image interpretability and leakage controls, first paragraph (deviation D35); Manuscript, Methods, Training and internal validation (deviations D37 and D38); Manuscript, Discussion, fifth paragraph (the tolerance near 0.006 on the calibration slope); Supplementary Materials, Supplementary Methods S6, Provenance of every reported difference; Supplementary Materials, Supplementary Results S5; Supplementary Materials, Supplementary Figure S1. All pre-specified performance is from a locked patient-level internal test cohort scored once after model freezing (deviation D31 in the accompanying deviation register), and consistency between the training and internal test sets is shown by the per-arm learning curves in Supplementary Figure S1. Every analysis added at revision is a further reading of predictions that already existed on that same, already-read split, performed by people who had seen its results, and is post hoc and exploratory (deviation D35); no model was refitted, re-tuned, re-ensembled or re-recalibrated. The artifact recording the single read carries no interpreter, device, platform or mixed-precision setting, so the float16-autocast provenance of the published estimates is documented only in the separate training record (deviation D37). Against a local float32 recomputation through the identical scoring function the maximum absolute difference in per-patient 5-year risk is 0.0096 and the rank correlation is 0.99994, while the locally recomputed area under the curve is 0.8367 against a published 0.8368, so aggregate discrimination is reproducible to about the fourth decimal place and an individual patient's risk only to about the second; predicted risks are therefore quoted to no more than two decimals and the 5-year calibration slope carries a tolerance near 0.006.
**METHODS - Evaluation**	**33**	Testing on external data	**X**			Manuscript, Abstract, Conclusions; Manuscript, Methods, Clinical comparators and statistical analysis, final sentence (No external validation is claimed); Manuscript, Discussion, fifth paragraph; Manuscript, Conclusion; Supplementary Materials, Supplementary Methods S8, Reporting. No external testing was performed; this is explicitly stated, justified as a limitation, and identified as a required next step.
**METHODS - Evaluation**	**34**	Clinical trial registration			**X**	Not applicable. This was a retrospective, non-interventional secondary analysis of a de-identified public dataset and not a clinical trial, so ICMJE clinical-trial registration does not apply. Stated for completeness because the item's guidance also mentions protocol registration: a full written study protocol was prepared before the analysis and is the reference against which every deviation is recorded, but it was not deposited in any public registry and no prospective registration of any kind was obtained. The Manuscript, Title page carries no registration statement of any kind; TRIPOD+AI item 18d scores that omission No under an item that is satisfied either by registration details or by an explicit statement of non-registration, which is a different requirement from this one.

Section / Topic	No.	Checklist item	Yes	No	NA	Location in the submission and rationale
**RESULTS - Data**	**35**	Numbers of patients or examinations included and excluded	**X**			Manuscript, Results, Cohort and image data; Manuscript, Table 1; Supplementary Materials, Supplementary Results S1; Supplementary Materials, Supplementary Figure S2. The source population, exclusions, final cohort, partition sizes, image usability and event counts are reported with a flow diagram: the 83,011 source-archive patients and the 3,709-patient landmark cohort in Supplementary Results S1 and Supplementary Figure S2, and the 741 test patients carrying 106 events, the 2,968 development patients carrying 427, the 6,071 written crops, the 1,216 test crops for 740 of the 741 patients and the one event-free test patient with no usable image in the body.
**RESULTS - Data**	**36**	Demographic and clinical characteristics of cases in each partition and dataset	**X**			Manuscript, Table 1 and its note; Manuscript, Results, Cohort and image data; Supplementary Materials, Supplementary Results S1. Table 1 compares development and test cohorts across demographic, clinical, imaging, follow-up and missingness variables.
**RESULTS - Model Performance**	**37**	Performance metrics and measures of statistical uncertainty	**X**			Manuscript, Tables 2 and 3; Manuscript, Results, The radiograph against the clinical record, What additional views contribute, Where the model looks, and what it is not reading, Prediction within Kellgren-Lawrence strata, Robustness strata, and Calibration and clinical usefulness; Supplementary Materials, Supplementary Tables S1, S2, S3, S4 and S5; Supplementary Materials, Supplementary Results S3, S4, S6, S7, S8 and S9. Discrimination, calibration, Brier scores, subgroup results, decision-curve findings and 95% confidence intervals are reported, together with the post hoc single-view contrasts, the Kellgren-Lawrence-stratified estimates, the imaging robustness strata, the occlusion and negative-control deltas and the per-view attention weights, each with its interval and its denominator.
**RESULTS - Model Performance**	**38**	Estimates of diagnostic performance and their precision	**X**			Manuscript, Tables 2 and 3; Manuscript, Results, Prediction within Kellgren-Lawrence strata; Supplementary Materials, Supplementary Table S5 Panels A, B and C; Supplementary Materials, Supplementary Table S1; Supplementary Materials, Supplementary Results S8. The prognostic analogues of diagnostic performance, including time-dependent AUROC and concordance, are reported with precision estimates. The revision identifies where the model works best and worst as far as these data permit: within the advanced-disease Kellgren-Lawrence 3-4 stratum, observed incidence by risk tertile within grade, and the imaging robustness strata, each reported with its event count and with the pre-specified suppression rule honoured rather than weakened. The Kellgren-Lawrence 0-1 stratum carries 3 events in 144 patients and is uninformative rather than null, and is reported as such. Class imbalance is inherent (106 events among 741 test patients) and is handled by the censoring-weighted estimators and the event-count floor rather than by resampling.

Section / Topic	No.	Checklist item	Yes	No	NA	Location in the submission and rationale
**RESULTS - Model Performance**	**39**	Failure analysis of incorrectly classified cases	**X**			Manuscript, Figure 2 and its caption (the four classification cells and the 0.134 operating point); Manuscript, Results, Where the model looks, and what it is not reading; Manuscript, Methods, Clinical comparators and statistical analysis, third paragraph (only 268 of the 741 test patients are classifiable at 5 years); Supplementary Materials, Supplementary Results S6; Supplementary Materials, Supplementary Methods S8, Classifiable denominators. Yes. The model output is a continuous time-to-event risk with no pre-specified binary threshold, so no confusion matrix is reported and the item's classification clause does not bind. Case-level failure material is nonetheless provided, and it is provided inside the submission rather than by reference to files a reader cannot obtain. Test patients were classified into true-positive, false-positive, true-negative and false-negative cells at an explicitly post hoc operating point, the risk quantile at which predicted positives equal observed cases, which is 0.134 on the published 5-year risk and is defined against the 263 of 734 patients who are classifiable at 5 years rather than against the whole cohort; the remaining 471 are censored before the horizon and cannot be placed in a classification table at all. Figure 2 shows one patient from each of the four cells, all matched at an inferred Kellgren-Lawrence grade of 2 so that a cell-to-cell difference in the maps is not a difference in disease severity, each presented as the radiograph the network reads with its Grad-CAM and its integrated-gradients overlay, and the caption gives each case's predicted 5-year risk, discloses that the false-negative case carries a residual burned-in character, and states that these four are the widest-spread quartet the sampler produced at that grade rather than a typical one. The occlusion, negative-control and per-view attention analyses in Supplementary Table S3, Supplementary Table S4 and Figure 3 supply the mechanistic context for incorrect predictions. A larger panel set of 32 cases, 8 per cell, with a manifest recording each case's grade, predicted risk, event status and follow-up time, was generated (outputs/tables/interp_panel_manifest.csv, outputs/interpretability/panels/) but is in neither the submitted figure bundle nor the public repository, which excludes both by name in its committed .gitignore as patient-level artefacts, so it is not evidence a reader can reach; it is available from the corresponding author on reasonable request, under the same restriction TRIPOD+AI item 18e records for patient-level derived data. The operating point was chosen after the single test read, so every threshold-specific statement is exploratory (deviations D35 and D36).

Section / Topic	No.	Checklist item	Yes	No	NA	Location in the submission and rationale
**DISCUSSION**	**40**	Study limitations	**X**			Manuscript, Discussion, fifth paragraph, which is now the whole limitations paragraph; Manuscript, Discussion, second and third paragraphs; Manuscript, Results, Manual review and burned-in markers, Where the model looks, and what it is not reading, Robustness strata, and Calibration and clinical usefulness; Manuscript, Methods, Image-quality and laterality verification, first paragraph; Supplementary Materials, Supplementary Methods S2, S6 and S7; Supplementary Materials, Supplementary Results S7. Limitations address the single-site design, the absence of external testing, competing mortality, the outstanding outcome-record review, the inferred-laterality exposures, architecture selection, subgroup power and post hoc thresholds. The revision adds: every new imaging, leakage, view-ablation, within-grade and robustness result is post hoc on an already-read test split (D35); the protocol section-23 two-reviewer image review was performed and reports raw agreement of 95% to 97.9%, Cohen kappa of 0.81 to 0.89 and a critical-error rate of 0.5% to 1.0% against a 2% threshold it clears, with the per-image scores held on a secure institutional system rather than released (D40); half-selection was verifiable only on the 318 development images whose panels carried the full film and never on the 82 test images, because the source films are gone, so that item's at-least-99% figure rests on 318 panels and not on 400; the protocol section 7 laterality audit was completed at 98.0% agreement on 196 of 200 development patients (Wilson 95% CI 95.0 to 99.2), adjudicated on paper and author-attested rather than recomputable, on a sample holding no test patient, so the recovered index side used for 49.3% of the cohort is validated indirectly rather than directly on the 371 test patients it recovered (D22); the section 23(ii) outcome-record review of 200 records is drawn and unadjudicated, and is the one manual review still outstanding (D22); the Kellgren-Lawrence grade is itself dataset-inferred, so prediction beyond conventional grading means beyond an automated grade rather than beyond a radiologist's read; no equipment, manufacturer or site metadata exists; acquisition era is uninformative rather than null because de-identified dates carry a per-patient random shift and are confounded with follow-up (D17); the masked border band is not exactly zero on 11.9% of test crops (D39); the pre-specified border-only negative control is degenerate and cannot support a leakage conclusion; the label-randomisation sanity check was not run (D36); and the environment-provenance gaps D37 and D38 leave the calibration slope with a tolerance near 0.006. The revision also states the estimand limit on the view result: a frontal-only model matches the multi-view arms across the whole cohort because 419 of the 740 test patients with a prepared crop, 56.6%, contributed a frontal radiograph and nothing else, which is a claim about this population and not evidence that the other views are uninformative.

Section / Topic	No.	Checklist item	Yes	No	NA	Location in the submission and rationale
**DISCUSSION**	**41**	Implications for practice, including intended use and/or clinical role	**X**			Manuscript, Discussion, fourth paragraph, which opens These findings support stratification, not quantification; Manuscript, Discussion, fifth paragraph, final sentence; Manuscript, Conclusion. The manuscript defines the counselling and planning use case, states that the model orders risk better than it quantifies it and that counselling on the timing or staging of a second procedure must rest on relative risk and observed incidence within a predicted-risk band rather than on the predicted probability, and states that external and temporally separated validation, the completed protocol reviews, competing-risk and powered fairness analyses and evidence that acting on predicted risk changes outcomes must precede clinical use and deployment. The description of the attribution maps as model-attribution visualisations rather than validated imaging biomarkers, which earlier drafts of this file cited, is not in either v6 document; what the submission carries instead is the statement that the whole interpretability package is post hoc and exploratory (Manuscript, Methods, Image interpretability and leakage controls, first paragraph; Manuscript, Figure 3 caption; Supplementary Materials, Supplementary Methods S6, Status).
**OTHER INFORMATION**	**42**	Provide a reference to the full study protocol or to additional technical details	**X**			Manuscript, Title page, Code Availability Statement; Manuscript, Methods, Clinical comparators and statistical analysis, final paragraph (deviations are registered); Supplementary Materials, Supplementary Methods S8, Reporting, which states that deviations are listed in the deviation register provided as accompanying material. The manuscript links the public code repository and archive and identifies the deviation register, which runs D1 to D40, provided as accompanying material. The written study protocol itself is not publicly deposited; its pre-specified procedures are reproduced in the Methods and the Supplementary Methods and in config/feasibility.yaml, which is in the public repository.

Section / Topic	No.	Checklist item	Yes	No	NA	Location in the submission and rationale
**OTHER INFORMATION**	**43**	Statement about the availability of software, trained model, and/or data		**X**		Manuscript, Title page, Code Availability Statement and Data Availability Statement. No. This item has three components and the entry is scored on all three. Data: met. The source-data access route, the CC BY-SA license, the documentation, the aggregate outputs, the withholding of patient-level derived data and the request procedure are all stated in the Data Availability Statement. Software: partly met, and not as the manuscript states it. The three modules that produced this revision's analyses (src/interpretability.py, src/v6_analyses.py and src/manuscript_figures.py) together with the aggregate v6 output tables were pushed to the public GitHub repository at commit b568820 on 2026-08-11, so the GitHub link in the Code Availability Statement now resolves to code that produces the reported analyses. The Statement also cites an archived Zenodo version, doi 10.5281/zenodo.21712883, and that archive is still frozen at the pre-revision commit dated 2026-07-30 and contains none of those modules. No new archived version has been minted, so the persistent, citable artifact the manuscript names does not hold the code for this revision, and the Statement's coverage of sensitivity analyses and generation of aggregate results remains true of the archive only for the v5 analyses. TRIPOD+AI item 18f is scored No on exactly this evidence and the two checklists agree. Trained model: not met. The manuscript nowhere states whether the trained model weights are available; they are not shared, so a third party can reproduce the method from the code but cannot reproduce the reported predictions, and TRIPOD+AI item 22 is scored No for the same omission. The record of the environment that produced the models (derived-data/cohort/train_arms.json) also sits in a git-ignored directory and is not in the public repository (deviation D38). This item becomes Yes when a new archived version is minted over commit b568820 or later and the manuscript cites that new DOI, and the manuscript adds one sentence stating that trained model weights are not publicly available together with the route for requesting them.
**OTHER INFORMATION**	**44**	Sources of funding and other support; role of funders	**X**			Manuscript, Title page, Funding statement. The manuscript states that the research received no external funding.

Checklist source: Tejani AS, Klontzas ME, Gatti AA, et al. Checklist for Artificial Intelligence in Medical Imaging (CLAIM): 2024 Update. Radiol Artif Intell. 2024;6(4):e240300.
