# Supplementary material for: Deep Learning Analysis of Routine Preoperative Knee Radiographs for Five-Year Prediction of Contralateral Arthroplasty
Source: J Imaging. 2026 Sep 30;12(10):474. doi: 10.3390/jimaging12100474 (PMC13705656; doi:10.3390/jimaging12100474)
Supplement: Supplementary file 1 [file jimaging-12-00474-s001.zip › jimaging-4534763-supplementary.pdf]

Journal of Imaging

MDPI

Supplementary Material

Deep Learning Analysis of Routine Preoperative Knee Radiographs for Five-Year Prediction of Contralateral Arthroplasty

Samer G. Salman [1,*,†], Rohan A. Phadke [1,*,†], Zane G. Salman [2,*,†], Ryan T. Zeitouny [3], Sai M. Yedupati [4], Joshua Ong [5], Alireza Tavakkoli [6], Sainyam Galhotra [7], Ajay Tripuraneni [1] and James Rizkalla [8]

[1] School of Medicine, Baylor College of Medicine, 1 Baylor Plaza, Houston, TX 77030, USA; ajay.tripuraneni@bcm.edu
[2] College of Natural Sciences, The University of Texas at Austin, Austin, TX 78712, USA
[3] Department of Biomedical Sciences, Texas A&M University, College Station, TX 77843, USA; ryanzeitouny@tamu.edu
[4] College of Engineering and Applied Sciences, University of Cincinnati, Cincinnati, OH 45221, USA; yedupasv@mail.uc.edu
[5] Massachusetts Eye and Ear, Department of Ophthalmology, Harvard Medical School, Boston, MA 02114, USA; joshong95@gmail.com
[6] Department of Computer Science, University of Nevada, Reno, NV 89557, USA; tavakkol@unr.edu
[7] Department of Computer Science, Cornell University, Ithaca, NY 14853, USA; sg@cs.cornell.edu
[8] Department of Orthopaedic Surgery, Baylor University Medical Center, Dallas, TX 75246, USA; james.rizkalla@bswhealth.org
* Correspondence: samer.salman@bcm.edu (S.G.S.); rohan.phadke@bcm.edu (R.A.P.); zgs277@eid.utexas.edu (Z.G.S.)
† These authors contributed equally to this work.

This document is part of the submission and holds the disclosures, parameter values and detail relocated from the Methods and Results of the main text. It contains Supplementary Methods S1 to S14, Supplementary Results S1 to S13, Supplementary Tables S1 to S10 and Supplementary Figures S1 to S6. Supplementary Methods S1 to S8 and Supplementary Results S1 to S9 carry the same headings as the Methods and Results of the main text, so any statement in the body can be resolved here; Supplementary Methods S9 to S14 and Supplementary Results S10 to S13 hold the analyses added at revision and have no main-text counterpart.

Supplementary Methods

This file holds the methodological detail, parameter values and protocol-deviation disclosures relocated from the Methods when the body was compressed for length. Sections S1 to S8 carry the same headings as the Methods, so any statement in the body can be resolved here, and nothing in them is new; every fact in those eight sections appeared in an earlier version of the Methods and is reproduced without change of meaning. Sections S9 to S14 were added at revision and are new. They give the missing-data mechanism and imputation method, a hyperparameter search for the pre-specified encoder, a second architecture, a fusion ablation, and the design of the outcome-record audit. The main text carries no protocol-deviation numbering; Supplementary Table S6 maps each main-text claim to the register entry that qualifies it, and this file keeps its own deviation citations.

S1. Radiographic dataset and image selection

 https://doi.org/10.3390/jimaging12100474

Eligibility. Patients were eligible if their earliest total knee arthroplasty (CPT 27447) was unilateral and interpretable, was performed at age 40 or older, was not preceded by contralateral arthroplasty, carried no infection or osteomyelitis exclusion, and was preceded by an interpretable contralateral knee radiograph 1 to 730 days beforehand. Follow-up began at a day-90 landmark, the outcome was contralateral arthroplasty within 5 years, and censoring was administrative at day 1826 or at last observed activity. In total, 3,709 patients qualified and formed the landmark cohort (Supplementary Figure S2).

Image selection. Radiographs entered through the dataset's own image-level annotations. A study was eligible when its knee-side annotation matched the contralateral side, or when the annotation read bilateral and the view was frontal, in which case the film shows both knees and the contralateral half is selected during preprocessing. Where a patient had more than one eligible pre-index study, a fixed preference order took a weight-bearing frontal film with lateral and sunrise views first, then a weight-bearing frontal with any additional view, then any frontal, then a non-weight-bearing frontal, with extra views preferred from the same study. Frontal, lateral and sunrise views were admitted and other view codes were dropped. A patient contributed one to five crops.

Splitting. Patients were partitioned once into locked 70% training, 10% validation and 20% test sets, stratified by 5-year event status, sex and major race group. The test split took no part in fitting, hyperparameter choice or checkpoint selection, and was scored exactly once under a gate that records that single read.

Crop counts by view. Across all splits the finished crops comprised 4,259 frontal, 1,651 lateral and 161 sunrise images. In the test split there are 858 frontal crops from 734 patients, 321 lateral and 37 sunrise.

The two frontal-only counts. Two frontal-only counts appear in this report and they classify different things. The 419 (56.6%) used in the body is a crop-based count on the 740 test patients with a prepared crop. The 418 is the count under the source dataset's own pre-preprocessing view-set label, which is the label the pre-declared imaging-views stratum uses, and which reads 418 frontal-only against 323 multi-view on the full 741-patient test roster and against 316 multi-view on the 734-patient frontal-view roster. Each count is stated with its own roster wherever it appears and neither is substituted for the other. Five multi-view denominators are in play and each is correct for a different population: 315 patients contributed a frontal crop and at least one non-frontal crop, which is the paired view-ablation denominator; 316 hold two or more distinct views; 321 contribute a non-frontal crop; 322 carry a non-frontal pre-preprocessing view-set label on the 740-patient fusion roster; 323 carry it on the full 741-patient roster.

Acquisition metadata. No acquisition parameter accompanies these images. MRKR releases 19 image-level columns and none records manufacturer, device model, station, institution, tube current, exposure or slice thickness. The 34.85 GB source DICOM archive is no longer held and the retained artifacts are 512 by 512 crops with the border band zeroed, so those tags cannot be recovered. Robustness by equipment, manufacturer and site therefore cannot be evaluated at all, and we report that rather than omitting the analysis.

S2. Image-quality and laterality verification

Index operative side (deviation D2). Where the earliest CPT 27447 record carried a right or left modifier the side was taken as coded. Sixty-one percent of CPT 27447 records in the source archive carry no modifier, and for those patients the index side was recovered by a deterministic rule from three record signals dated on or before the index procedure: the

laterality annotation of an image from the same date, a laterality-specific ICD-10 M17 diagnosis, and the free-text study description of a same-day study. Recovery required exactly one side across the three signals with no conflicting signal; a patient with conflicting or absent signals was excluded. No post-index or outcome information was used. The index side was coded for 1,881 patients and recovered for 1,828, that is 49.3 percent of the 3,709-patient landmark cohort, with a comparable split in the locked test set (370 coded, 371 recovered).

Section 7 laterality audit (deviation D22). Protocol section 7 requires a manual laterality audit of at least 200 patients, over-sampling recovered patients, before the cohort is locked. That audit was performed. The deterministic index-side recovery rule agreed with manual adjudication in 196 of 200 sampled development patients, 98.0 percent, with a Wilson 95 percent confidence interval of 95.0 to 99.2 percent and a 2.0 percent observed error rate. The method is named because it matters at this boundary: Agresti-Coull gives 94.8 to 99.4 percent and Clopper-Pearson 95.0 to 99.5 percent, while the normal approximation gives 96.1 to 99.9 percent, which is not a defensible interval for 196 of 200 and is not what we report. The adjudication was performed on paper, the per-patient reviewer records are held offline and are not digitised, and no sampling workbook or audit summary table accompanies the release, so this figure is author-attested rather than recomputable from the code and tables published with this report. The audit is also asymmetric: its 200 patients are 168 training and 32 validation, and it holds no test patient at all. Which side the index knee was operated on is answerable only from a full film, and the source films for the test split are gone, so a test patient could only be entered as not assessable. The audit therefore validates the recovery rule on development patients and never on the 371 test patients whose index side was recovered by rule. Split assignment is unrelated to laterality, so a development-only sample remains an unbiased estimate of the cohort-wide error rate, but it is an estimate rather than a direct check of the test split. The outcome-record audit (S14) later checked the index side of the 101 test-split patients in its sample against the billing record, and Supplementary Results S13 reports the result. Protocol section 7 states that the audit passes only if the reviewed index side matches the coded or recovered side for every row; four rows disagreed, and the cohort lock was not re-opened on them. The audit is therefore complete and reported rather than passed, and the 2.0 percent observed error rate is the disclosure that stands in place of that gate.

Dataset-supplied image annotations. MRKR distributes knee side, view position, horizontal flip, weight-bearing status and photometric inversion as model-inferred annotations produced by classifiers trained on roughly six thousand annotated images, and publishes an accuracy figure for only some of them. Knee side and view position determine which radiographs were eligible and how each was labelled as frontal, lateral or sunrise. The horizontal-flip flag determines which half of a bilateral film contains the contralateral knee, because 4,069 of the 6,071 prepared crops came from films showing both knees. The inversion flag was cross-checked against each DICOM's own photometric interpretation tag on every decode and the tag was allowed to override it, which it did on 6 of 1,218 scheduled test images. The horizontal-flip flag cannot be cross-checked, because no DICOM tag records it; it altered the selected half on 287 frontal films, 63 of them in the test set, and an error there would present the already-replaced knee to the model.

Section 23 manual image review (deviation D40). Protocol section 23 requires two independent reviewers with orthopaedic or musculoskeletal imaging experience to score at least 400 images for laterality, view, native-knee status, crop adequacy, burned-in text and non-knee content, with raw agreement, Cohen kappa, third-reviewer adjudication and a 2 percent critical-error threshold. A sample of 400 images over 393 patients was drawn across

all three splits, 273 training, 45 validation and 82 test, stratified on split, view, contralateral side and bilateral or unilateral acquisition. Two reviewers with musculoskeletal imaging experience then scored that sample independently, without conferring and without comparing sheets, and their disagreements were adjudicated afterwards in a separate recorded step. Each reviewer made 2,318 judgements, 400 on each of the five items decidable from a finished crop and 318 on laterality, so 4,636 across both. Each image was read once by each reviewer, so the design yields an inter-rater estimate and no intra-rater estimate.

The result, reported as ranges and bounds across the six items. Raw agreement between the two reviewers ran from 95 to 97.9 percent, and Cohen kappa from 0.81 to 0.89. The critical-error rate ran from 0.5 to 1.0 percent against the pre-specified 2 percent threshold, so the review clears that threshold on every item and no expansion was triggered. Per item, laterality, view, native-knee status and freedom from non-knee content were each correct on at least 99 percent, crop adequacy on at least 98 percent, and at least 95 percent of crops were clean of burned-in text. These figures are the ranges and bounds the reviewers' summary reports; we do not convert a bound into a point estimate, average a range, or derive a per-item kappa from them. The per-image adjudications are held on a secure institutional system and are not part of the release, so what accompanies this report is the summary statistics rather than the per-image scores.

Laterality was scored on 318 of the 400 images, and no review could have reached the other 82. Five of the six items, that is view, native-knee status, crop adequacy, burned-in text and non-knee content, are decidable from a finished crop and were scored on all 400 images. The sixth, the selected half, is not decidable from a crop at all: every pre-index film shows two native knees, right knees are mirrored to a common orientation and the border mask removes any burned-in side marker, so the crop is anatomically identical whether the correct half or the wrong half was taken. Reviewer panels for the 318 development images showed the full film beside the crop, so the selected half was judged there; the source images for the test split are gone, so its 82 panels showed the finished crop alone and were issued already marked not assessable on that item. The at-least-99-percent laterality figure therefore rests on 318 panels, all in development, and half-selection on the reported split is unverified by construction rather than by oversight. The outcome-record review required by section 23(ii) was completed at revision. Its pre-specified 200-record sample was kept unchanged and a 60-record test-split extension took it to 260 records; two reviewers scored every record, with no disagreement requiring adjudication (S14, with results in Supplementary Results S13). Deviation D22 records that the reported results rest on reviews that were incomplete when the locked split was opened. This image review and the outcome-record review were both scored after that split was read, so they verify the crops and the labels the reported estimates rest on rather than gating them.

Burned-in markers, four automated rates and one human read. Three separate automated measurements of burned-in markers exist and each carries its own sample and pipeline stage. Before masking, 26.4 percent of 500 sampled training crops carried a marker inside the border band (frontal 15.8, lateral 52.7, sunrise 50.0 percent). After masking, a residual audit of 72 finished development crops found a surviving marker-like blob on 16.7 percent, and an automated scan of all 1,216 finished test crops found one on 18.5 percent (frontal 9.2, lateral 43.0, sunrise 21.6 percent). The two post-masking figures are the same quantity on different splits and both are upper bounds, because saturated bone edges share the detector's signature; the pre-masking figure is not comparable to either. The same scan established that the masked border band is not exactly zero on 145 of the 1,216 test crops (11.9

percent), with a maximum value of 48 of 255 affecting 0.049 percent of band pixels, because marker masking runs after border masking and refills each accepted blob with the surrounding ring median. That is a departure from the section 13 contract, recorded as deviation D39; its measured cost is given in the leakage analysis. The section 23 review adds a human read of the same question on a different sample: at least 95 percent of the 400 sampled crops were clean of burned-in text. It is not a fifth denominator for the automated rates and must not be pooled with them. The automated scan flags any marker-like blob and so bounds above, because saturated bone edges share its signature; the reviewers judged whether text or a marker is actually visible.

S3. Image preprocessing

Decoding and geometry. Each DICOM was decoded with rescale slope and intercept applied, the value-of-interest lookup table applied, MONOCHROME1 images inverted so that higher values are brighter, and intensities clipped at the 0.5th and 99.5th percentiles of that image before scaling to 8-bit greyscale. Geometry followed the radiological display convention, in which the patient's right knee appears on the image left, corrected first by the dataset's horizontal-flip annotation. On a bilateral film the half holding the contralateral knee was taken with a 2 percent inset from the midline, so that no index-knee pixel could survive an off-centre patient. A square region was cropped from that half, centred on the collimated field at 95 percent of its short side, resampled to 512 by 512 pixels with Lanczos interpolation, and finally mirrored where necessary so that all 6,071 crops read as a left knee.

Joint localization. Localization was deliberately conservative. A centred box is a strong prior because radiographers centre the knee in the field, and a classical intensity-profile localizer measured on 800 training images was worse than doing nothing: it fell back to the centre on 26.0 percent of films, and visual review showed that its successes frequently locked onto a bright femoral or tibial shaft and dragged the box off the joint. The centred box was therefore made the primary path and the localizer demoted to a refinement that may move the box only above a confidence of 0.60, against an observed median confidence of 0.21. In the finished set the centred box was used on 5,985 of 6,071 crops and the refinement on 86, with a mean crop confidence of 0.9957.

Border masking. The outer 31 pixels of every crop were set to zero, which blanks 22.75 percent of the output. That figure is the integer band the pipeline actually zeroes, 1 minus (450/512) squared; the continuous form gives 22.56 percent and is not what the pipeline does, because 0.06 times 512 rounds to 31 rather than 30.72. Out-of-bounds padding and any accepted marker blob both add to the band on individual images, so the recorded masked fraction had a mean of 23.09 percent, a 90th percentile of 23.64 percent and a maximum of 44.75 percent, and 1,572 of 6,071 crops carried masked pixels beyond the band. That count is the number of crops whose recorded masked fraction exceeds the band, so it counts padding, marker masking or both and is not a count of padded crops alone. Crops with more than 45 percent masked were excluded.

Marker masking (deviation D39). Because the fixed band alone was not sufficient, a marker detector then searched the retained region. A candidate blob had to be saturated at or above a value of 250, cover at least 20 pixels and no more than 1 percent of the crop, and sit on background dark enough that the median of a 6-pixel surrounding ring was at or below 60. Candidates were dilated by 10 pixels so that multi-character text was removed whole rather than one letter at a time, and each accepted region was filled with its own ring median. This step runs after border zeroing, which is the mechanism behind deviation D39.

https://doi.org/10.3390/jimaging12100474

Augmentation and the masking gate. Augmentation was capped at rotation of 5 degrees or less, translation and scale of 5 percent or less, and brightness and contrast perturbation of 10 percent or less, with nothing that deforms joint-space geometry and specifically no random resized cropping. The border band was re-zeroed after augmentation. Nineteen images were excluded by the section 13 masking gate between the selected manifest of 6,090 images and the 6,071 written crops, 17 in development and 2 in the test split; none was excluded for a localization failure, because a centred crop is a valid outcome rather than a failure.

S4. Network architecture and multi-view aggregation

Architecture. The image arms share one DenseNet121 encoder across views, initialized from ImageNet weights. Each crop a patient contributes is encoded separately, a learned 32-dimensional view embedding is added to the pooled feature vector, and an attention pool over that patient's available views returns a single patient-level representation. Patients contribute different numbers of views, so the pool is masked and padded slots are never passed through the encoder at all. A linear head emits the probability of replacement in each of ten six-month intervals among patients who reach it, and those interval hazards combine into a cumulative 5-year risk. In the fusion arm the pooled image vector is concatenated with the frozen standardized clinical vector before the head. A censored patient remains in the risk set until observation ends, and the loss is the censoring-aware negative log-likelihood of the discrete-time model.

The nine arms. M0 is a frozen ridge-penalized Cox model over eleven routinely recorded clinical predictors and M1 adds the dataset-inferred contralateral Kellgren-Lawrence grade on the subset where a grade is recorded. M0d and a discrete-time M1 are the corresponding discrete-time controls, which separate the effect of the survival model from the effect of the imaging. M2 is the radiograph model on the frontal view alone, M3 on all available views, M4 the multimodal fusion of all views with the clinical vector and the pre-specified primary arm, and M4-frontal the fusion arm restricted to the frontal view. R1 is the single robustness architecture.

Encoder substitution (deviation D28). ConvNeXt-Tiny was the pre-specified encoder, and under the one configuration tried before the split was read it did not fit these training data. The change was decided under a configuration that was superseded before any reported number was produced, and the figures D28 published for that configuration are withdrawn. A correction to that entry dated 2026-09-04 records that none of them is backed by a surviving artifact, including the round base rate of 0.610, and this report quotes none of them. What reproduces is the retrained ladder that produced every number reported here: frontal-view ConvNeXt-Tiny reached a training negative log-likelihood of 0.607 averaged over its five seeds and 0.603 at its single best seed-epoch, against 0.371 and 0.357 for DenseNet121 on the same frontal crops. Read against each arm's own mean first-epoch training negative log-likelihood in `outputs/tables/train_history.csv`, 0.611804 for ConvNeXt-Tiny and 0.612705 for DenseNet121, that is a fall of 0.76% against one of 39.4%. DenseNet121 therefore became the primary encoder and ConvNeXt-Tiny was retained on frontal views as the single robustness architecture, so every comparison across encoders is exploratory rather than a pre-specified test. Protocol section 25 asks for a simpler DenseNet121 frontal-image model as the robustness architecture, and DenseNet121 is now the primary encoder, so what was run meets the intent of that section and not its wording. That contrast rests on a single configuration of the abandoned encoder, which is the gap the revision-stage hyperparameter sweep was registered to close; S11 gives the sweep and Supplementary Results S10 reports what it found.

https://doi.org/10.3390/jimaging12100474

S5. Training and internal validation

Optimization (deviation D26). Every image arm carried the same regularization: dropout of 0.3 in the risk head, AdamW weight decay of 0.05, gradient-norm clipping at 1.0, a cosine schedule with two warm-up epochs, and the encoder frozen for the first three epochs so that a randomly initialized head could not drag the backbone before it had learned anything. The encoder trained at a learning rate of 1e-4 and every randomly initialized component at ten times that rate, with the head initialized at 0.1 of its normal scale. The effective batch size was 32, formed as a micro-batch of 8 with 4 gradient-accumulation steps, which is deviation D26. Training ran to a 40-epoch budget and stopped when validation negative log-likelihood had not improved for 8 epochs; the retained checkpoint is the epoch at that minimum. Five pre-specified seeds per arm were combined by averaging the predicted interval hazards and converting to risk once.

Recalibration. A horizon-specific recalibration was fitted on the validation split alone and frozen. It is a complementary log-log transform, cloglog (P) = intercept + slope times cloglog (p-hat), estimated separately at each horizon: at day 1825 it is 0.3469 + 1.6383 times cloglog (p-hat) for the fusion arm and 0.1644 + 1.5622 for the frontal-view arm. It is applied to the image arms only; the two frozen Cox comparators are published as fitted. Every predicted risk reported in this manuscript, including every risk shown in the figures, is on that recalibrated scale, and the transform is strictly monotone at every horizon of every arm, so it changes the spacing of predicted risks without changing their order.

Convergence. Two convergence quantities must be kept apart. At the retained checkpoint the four DenseNet121 image arms carried a training-to-validation negative log-likelihood gap of 0.023 to 0.052 (0.028 frontal view, 0.052 all views, 0.023 frontal-view fusion, 0.052 fusion), in the same range as the 0.036 and 0.052 of the two clinical arms; the ConvNeXt-Tiny arm sits below both groups at 0.009. The two largest gaps, the all-views image arm and the clinical-plus-grade arm, agree to three decimal places and differ by about one part in five thousand, so the image arms are not separated from the clinical arms on this quantity in either direction. Because training loss is recorded in training mode with dropout and augmentation active and averaged across each epoch while the weights update, whereas validation loss is a single clean evaluation pass, these gaps are lower bounds of unknown tightness rather than like-for-like measurements. Separately, the convergence table's overfitting diagnostic is validation loss at the last epoch minus its own minimum, and because the patience is 8 the last epoch is always exactly eight epochs past the retained checkpoint. Supplementary Figure S1 gives the learning curves for all seven arms and all 35 arm-seed series, aligned on the retained epoch. The test split took part in neither checkpoint selection nor recalibration.

Environment (deviations D37 and D38). The models reported here were trained, and the sealed split was scored, on a cloud graphics processing unit under float16 automatic mixed precision, with Python 3.12.13, PyTorch 2.11.0 and timm 1.0.28; that record is held in the frozen training-arm file. The artifact recording the sealed read itself carries no interpreter, device or precision field (deviation D37), and the environment file published beside the code describes the local workstation used for the revision analyses rather than the environment that produced the models (deviation D38).

S6. Image interpretability and leakage controls

Status (deviations D35 and D36). Protocol section 22 pre-specified attribution mapping, regional occlusion, negative controls, randomization sanity checks and a masked-text sensitivity test. None had been run at first submission and no deviation covered the gap; the

https://doi.org/10.3390/jimaging12100474

package was executed at revision stage and is recorded as deviation D36. Every quantity in it is post hoc on an already-read split and reported as exploratory under deviation D35. No model was refitted, re-tuned, re-ensembled or re-recalibrated for it.

Attribution. Attribution was computed for the frontal-view model with two methods, both averaged over the five pre-specified seeds and both taken with respect to the predicted 5-year risk. Gradient-weighted class activation mapping was taken at the encoder's final normalization layer, whose native resolution is 16 by 16, on all 858 frontal test crops from 734 patients. Integrated gradients were computed at pixel resolution with 32 midpoint steps and a zero baseline, on 230 crops from 200 patients; that sample was chosen rather than exhausted, because integrated gradients cost 64 forward and backward passes per seed per image. The two denominators are not interchangeable and are stated wherever either result appears.

Regions. Three regions partition the crop: a tibiofemoral joint box at rows and columns 128 to 384, the central half of the 512-pixel crop in each dimension and 25.00 percent of its area; the 31-pixel masked border band, 22.75 percent; and the remaining peripheral background, 52.25 percent. Three further regions subdivide the joint box: medial and lateral halves at 12.50 percent each, and a patellofemoral zone at rows 128 to 256 and columns 192 to 320, 6.25 percent. Because every crop is mirrored to read as a left knee under the radiological convention, the medial aspect is the image-left half of the joint box, which was confirmed on the images by the position of the fibular head. For each map we report the fraction of absolute attribution mass in each region and its enrichment against that region's share of the image.

Anatomic registration of the region. A fixed central box is defensible because the crop is a square region centred on the collimated field, but we tested it rather than asserting it. A model-free matched-filter joint-line detector run over all 858 frontal test crops placed the detected joint line inside the box rows on 91.0 percent of images and the limb centre inside the box columns on 98.5 percent, with both landmarks inside on 90.1 percent. Horizontal centring is looser than vertical, so the whole attribution analysis was repeated on a per-image anatomically registered region of the same shape, re-centred on each crop's own joint line and limb centre. That region overlaps the fixed box with a mean intersection over union of only 0.42 and occupies 20.7 percent of the image, which makes it a genuine sensitivity analysis rather than a restatement.

Occlusion and negative controls. Occlusion analyses re-scored the frozen five-seed ensemble on perturbed images. Occluded pixels were set to zero, which is what the pipeline itself writes into the band and into out-of-bounds padding, and the two tibiofemoral conditions were repeated with a mean fill as a sensitivity to that choice. Regional conditions occluded the medial compartment, the lateral compartment, the patellofemoral zone, the border band and the whole joint box. One negative control stands, and it supplied the joint box alone. A second was pre-specified, supplying the border band alone, and it is withdrawn from the paper: preprocessing already zeroes that band, so supplying the band alone hands the network a near-constant input, and any discrimination that follows measures the destruction of the image rather than a property of the model or any burned-in text (deviation D36). No result from it is reported here or anywhere else in this report. Masking sensitivity blanked every residual saturated marker-like blob across the test split, using the looser of the two available blob criteria so that the result is an upper bound on what markers could contribute, and separately widened the masked band from 31 pixels to 62 and to 93.

Randomization checks. Attribution was re-run after cascading randomization of the model parameters, in which randomizing a block also randomizes everything above it and the head. Map similarity is reported as a Spearman correlation restricted to imaged pixels,

because a whole-image correlation is inflated by the agreement of any two maps that the blanked band and the black collimation margin are cold. Map statistics rest on 60 sampled crops and discrimination on the arm's whole 734-patient roster, and the two denominators are reported separately. The second sanity check section 22 names, re-running attribution after randomizing the outcome labels, requires retraining on permuted labels and was not performed; that is a scope cut recorded in deviation D36 and is claimed nowhere as done.

View attention and view withholding. Per-view attention weights were read out of the frozen aggregators of the two multi-view arms. Weights sum to one over the views a patient actually contributed, so a patient holding only a frontal film contributes a weight of one to it trivially, and the interpretable comparison is within patients holding one crop of each of two views. We also withheld views at input from the same frozen networks. Because one condition requires a frontal crop and another requires a non-frontal crop, that analysis is restricted to the intersection of all four conditions' rosters, which is the 315 test patients who contributed both a frontal crop and at least one non-frontal crop.

Provenance of every reported difference. One provenance convention governs the package. Cases for the display panels were selected on the published risks, so that a case falls in the same cell of the classification table as every published estimate implies. Every difference was instead formed from locally recomputed float32 predictions produced in one process on one device through the identical scoring function, so that no difference measures float16 against float32. Agreement between the two paths is close but not exact: the maximum absolute difference in per-patient 5-year risk is 0.0096, the rank correlation is 0.99994, and the locally recomputed area under the curve is 0.8367 against a published 0.8368. Aggregate discrimination is therefore reproducible to about the fourth decimal place while an individual patient's risk is reproducible only to about the second, and individual risks should be read with that tolerance. Uncertainty came from the same paired patient-level bootstrap of 2,000 replicates and the same seed used for the primary analysis, so a given replicate is the same set of patients here as there.

S7. Imaging robustness and ablation analyses

Strata (deviation D35). Discrimination was re-estimated within strata of image acquisition and image quality: weight-bearing status, view availability, the source of the index laterality, the amount of border and marker masking, crop confidence, joint-localization method, photometric inversion, bilateral half-selection and acquisition era. Every stratum is a mask over predictions that already existed, so none required a further read of the test split, and all of it is post hoc under deviation D35.

Suppression and undefined cells. The pre-specified 50-event suppression floor was applied unchanged. A cell can be blank for two different reasons and the two are not interchangeable. Most suppressed cells fall below the floor. A minority are undefined rather than imprecise: the 5-year time-dependent area under the curve compares patients with an event by the horizon against patients observed event free beyond it, and only 162 of the 741 test patients reach day 1826, so a level can clear the event floor and still hold no eligible control at all. Every stratum is therefore also reported on Harrell concordance, which is horizon free, and every estimated cell states its case and control counts.

Two masking definitions. The image-quality strata are reported under two definitions, over all of a patient's crops and over the frontal crop alone. These are different denominators and, for masking, different conclusions, so they are labelled and never averaged. Crop confidence and localization method are one partition and not two: a confidence below 1.0

holds if and only if the intensity-profile localizer was used, on all 6,071 images. The horizontal-flip flag is described and not charted, because it is set on 63 test images carrying 7 events in total and no partition of that level can reach 50 events.

Acquisition era (deviation D17). Era was analysed under three pre-declared schemes and all three are reported: three equal five-year calendar spans, which use no data at all to place an edge; the study-year tertiles of the development split; and the development split's median study year. No edge is a function of a test event count. An edge placement that would have left two estimable era cells exists and was not adopted, because choosing edges to maximize estimability selects on the outcome. Two caveats attach to every era result. The de-identified study date carries a per-patient random shift, so absolute calendar dates are not comparable across patients; protocol section 17 requires written confirmation that they are, and that confirmation has never been obtained (deviation D17). And era is nearly collinear with follow-up and record availability: on the test split, median follow-up falls from 1,826 days in 2007 to 2011 to 430 days in 2017 to 2021 while the share observed through five years falls from 97.2 to 1.0 percent, and there is one event among the 303 cohort patients dated through 2012, none of them in the test split.

Two view estimands. Two different questions about views are answered by two different designs, and they are not interchangeable. Comparing separately trained arms over the whole test split asks what a deployed frontal-only model would lose on this population, which includes the majority of patients who have no second film. Withholding a view at input from one frozen multi-view network asks what a second film contains, and can only be asked of patients who have one. We report both, label each with its own population, and generalize neither to the other's question.

S8. Clinical comparators and statistical analysis

Comparators (deviations D20, D21, D23 and D24). Two frozen clinical comparators were replayed from stored coefficients: a ridge-penalized Cox model over eleven routinely recorded predictors (age, sex, the radiograph to index interval, knee pain and comorbidity), and a second adding the dataset-inferred contralateral Kellgren Lawrence grade where it is recorded (2,500 training and 359 validation patients). The clinical model's ridge penalty was chosen by cross-validation inside the training split and sits at the lower edge of the pre-specified grid, which was not widened (deviations D23 and D24). Predictors used only data dated on or before the index procedure, and missing values took a training-split median or mode. Two of the clinical model's predictors depart from their pre-specified definitions: the comorbidity flags use all pre-index history where the protocol's predictor table specifies the prior five years (deviation D20); and the pain predictor is the highest knee pain score recorded in the prior 365 days where that table specifies the most recent contralateral or bilateral score in the prior 180 days (deviation D21). A longer comorbidity lookback can only raise prevalence. A maximum rises with the number of pain encounters, so it carries utilisation as well as severity. One censoring curve, from the clinical model, weights every arm, so none is advantaged by its own estimate of follow-up.

Estimand and uncertainty. The pre-specified primary estimand is the difference between the combined and the clinical model in the probability that a patient who has the operation is ranked above one who does not, at days 365, 730 and 1825, weighted for patients lost to follow-up. Harrell's and Uno's concordance, the calibration slope, calibration-in-the-large and the Brier score were also computed. Day 1825 is used at 5 years because administrative

censoring lands on day 1826, where the estimator is undefined. Uncertainty came from a paired patient-level bootstrap of 2,000 replicates sharing one resample across arms.

Intersections. Arms score different populations, because an arm scores a patient only where the views it needs survived the crop pipeline and the grade arm is defined only where a grade was observed. Every contrast is therefore computed on the intersection of the two arms it compares, and an arm's estimate changes between contrasts as that intersection changes: the clinical model scores 0.682 on all 741 patients, 0.680 paired against the fusion model on 740 and 0.675 paired against the frontal-view model on 734. A difference between two arms is never formed by subtracting two marginal levels.

Multiplicity and suppression. The primary contrast is unadjusted; the pre-specified secondary contrasts went to the modality, robustness and views families with Benjamini-Hochberg adjustment within each. The contrasts added at revision form their own families of two and are adjusted inside those alone, so that no published adjusted p value changes. Subgroup and stratum estimates below 50 events are suppressed. A p value at the bootstrap resolution floor of 0.0005 is reported as P < 0.001, in the unadjusted and the adjusted column alike.

Prediction within radiographic severity. Prediction within radiographic severity was assessed three ways. The grade takes nine values on the test split, including averaged half-grades where a patient contributed more than one frontal read, and is missing for 34 of the 741 patients, leaving 707 with 98 events. Bin edges were declared once at 1.5 and 2.5 on the numeric grade, giving three left-closed strata (grades 0 to 1, grade 2, grades 3 to 4), so a tied half-grade joins the higher stratum on the ground that a knee averaging 2.5 had at least one frontal read of grade 3; because that is a real choice, the whole stratification was re-run with the opposite tie rule and with a single coarse cut at 2.5, and both are reported. Suppression here is arithmetically unavoidable rather than a shortfall of effort: this population carries 98 events, two strata each clearing a 50-event floor need at least 100, so no partition of it can leave more than one estimable stratum, and the stratum that clears is necessarily the advanced-disease one. We therefore lead with two analyses that do not depend on that estimator. The first is the observed 5-year Kaplan-Meier incidence by tertile of predicted risk formed inside each stratum, with Greenwood intervals and an exploratory log-rank test across tertiles. The second is a Cox model in which the grade enters as a covariate and the model risk score is added to it. The score is the complementary log-log of the arm's 5-year predicted risk standardized to unit standard deviation, which is a linear-predictor scale and is invariant to the frozen recalibration, since that recalibration is affine on exactly this scale. The grade enters as the raw numeric value in the primary specification, with a categorical and a stratified-baseline specification as sensitivities, and each specification is compared by likelihood ratio on one degree of freedom against the identical model without the score. Proportional hazards was examined with Schoenfeld residual tests on a rank time transform.

Decision-curve analysis (deviation D29). Clinical usefulness was summarised by decision-curve analysis, pre-specified in kind but post hoc in timing: its estimator, thresholds and arms were fixed after the single read (deviation D29), so every threshold-specific finding is exploratory. Net benefit was estimated by Kaplan-Meier within the flagged set rather than by per-patient inverse-probability weighting, because 63.8 percent of test patients are censored before the horizon and the within-set estimator needs only censoring independent of outcome given membership of the flagged set. The threshold grid was built on an integer-percent basis from 1 to 35 percent and anchored to the development 5-year risk, so no test

https://doi.org/10.3390/jimaging12100474

quantity entered its choice; the plotted window is 2 to 30 percent and each curve stops where its flagged set falls below 15 events.

Classifiable denominators. One further denominator governs any statement about predicted positives. Of the 741 test patients, only 268 are classifiable at 5 years, being the 106 with an event by then plus the 162 observed event free beyond the horizon; the remaining 473 are censored earlier and cannot be placed in a classification table at all. On the 734 patients the frontal-view model scores, the corresponding counts are 263 classifiable, 106 cases and 157 controls, with 471 censored earlier. Any operating point, sensitivity, specificity or cell of a classification table describes that classifiable subset and not the cohort.

Reporting. Reporting follows the TRIPOD+AI and CLAIM guidance; completed CLAIM 2024 and TRIPOD+AI checklists accompany this report as supplementary material, and the sample was checked against the Riley criteria. Deviations are listed in the deviation register, provided as accompanying material. This is a development and internal-validation report; no external validation is claimed.

S9. Supplementary tables added at revision

Protocol deviation cross-walk (Supplementary Table S6). Reviewer 2 asked that the technical deviation numbering leave the main text and sit in the supplementary material. It has: the main text and its tables now carry no deviation number. Supplementary Table S6 replaces that numbering with a cross-walk. For each of the sixteen register entries that qualify a main-text claim, it gives the claim that entry qualifies, quoted from the text, the section the claim sits in, what the entry records, and the entry's date and status, so a reader can still resolve any statement in the body to its disclosure. This supplement keeps its own deviation citations in place, because a reader at this level of detail is reading the register beside it.

Comparison with prior work (Supplementary Table S7). Reviewer 1 asked what this study contributes beyond earlier deep-learning work on knee osteoarthritis and arthroplasty prediction. The study claims no algorithmic novelty and never did: DenseNet121, gradient-weighted class activation mapping, integrated gradients and attention pooling are all established methods used as published. Supplementary Table S7 sets this study beside the work the Introduction cites [9-14] on the three things each paper's own title and citation settle, which are the cohort and acquisition setting, the outcome predicted and the imaging input, and it fills that row for this study in full. The table makes no per-study claim about what any of those papers reported on the contrast inside a fixed radiographic grade, on quantitative attribution and leakage control, or on view necessity. Settling those would need each full text, which this report does not hold, and we do not characterise another group's work from its title. The table note carries the contrast at the level the Introduction already asserts: this literature predicts mostly radiographic endpoints, and predicts arthroplasty for the imaged knee in research cohorts with standardized acquisition, quantitative attribution and occlusion experiments are rare in it, and leakage controls on burned-in text and borders are rarer still.

S10. Missing data: mechanism, imputation and sensitivity

Which values are missing. Twelve clinical variables enter a model. Ten of them are complete on every one of the 3,709 landmark-cohort patients. The two that are not are the maximum pre-index knee pain score and the dataset-inferred contralateral Kellgren-Lawrence grade, and each is absent because the underlying record or frontal read does not exist rather than because a value was lost. Counts below are patients with no value before imputation, from `outputs/tables/features_clinical_completeness.csv`.

Variable	Enters	Missing overall, n (%)	Training, n (%)	Validation, n (%)	Test, n (%)	Training-split fill
Age at index	M0, M1	0 (0.00)	0 (0.00)	0 (0.00)	0 (0.00)	66.0, never used
Female sex	M0, M1	0 (0.00)	0 (0.00)	0 (0.00)	0 (0.00)	1, never used
Radiograph-to-index interval	M0, M1	0 (0.00)	0 (0.00)	0 (0.00)	0 (0.00)	98 days, never used
Any pre-index knee pain	M0, M1	0 (0.00)	0 (0.00)	0 (0.00)	0 (0.00)	1, never used
Maximum knee pain score	M0, M1	268 (7.23)	172 (6.62)	36 (9.70)	60 (8.10)	7.0
Obesity	M0, M1	0 (0.00)	0 (0.00)	0 (0.00)	0 (0.00)	0, never used
Diabetes	M0, M1	0 (0.00)	0 (0.00)	0 (0.00)	0 (0.00)	0, never used
Hypertension	M0, M1	0 (0.00)	0 (0.00)	0 (0.00)	0 (0.00)	1, never used
Autoimmune disease	M0, M1	0 (0.00)	0 (0.00)	0 (0.00)	0 (0.00)	0, never used
Nicotine use	M0, M1	0 (0.00)	0 (0.00)	0 (0.00)	0 (0.00)	0, never used
Lower-extremity trauma	M0, M1	0 (0.00)	0 (0.00)	0 (0.00)	0 (0.00)	0, never used
Inferred contralateral Kellgren-Lawrence grade	M1 only	143 (3.86)	97 (3.74)	12 (3.23)	34 (4.59)	never imputed

A fill value is listed for every predictor because the imputer fits one for every column it is given, but on the ten complete predictors it is never applied to a single patient. Only the maximum knee pain score is actually filled anywhere in this study.

How the two incomplete variables were handled. Imputation is a single unconditional constant per column, fitted on the training split alone and then frozen. The fill is the median for a numeric column and the most frequent level for a categorical one, estimated on the 2,597 training rows and on nothing else, written to `derived-data/cohort/clinical_imputation_params.json`, and replayed unchanged on the validation and test splits, so no downstream module ever refits and no held-out patient contributes to a statistic that is then applied to them ('src/features_clinical.py:233', 'config/feasibility.yaml:657'). The fitting function asserts that its frame holds training rows only and fails loudly if a non-training row reaches it. The maximum knee pain score takes a fill of 7.0, from 172 missing training values of 2,597. No multiple imputation, chained-equation model or learned imputer was used; the imputation model is the constant, and it is published.

The grade is never imputed for any model. The inferred contralateral Kellgren-Lawrence grade is an M1 predictor alone. M1 is fitted only on patients whose grade is observed, which is 3,566 of the 3,709 patients, comprising 2,500 training, 359 validation and 707 test patients carrying 351, 52 and 98 events ('src/features_clinical.py:873', 'config/feasibility.yaml' 'm1_requires_klg_eligible_subset: true'). A median of 2.0 exists in the frozen parameter file and is written to an auditing column, and no model reads it. Imputing a radiograph-derived severity grade across the whole cohort would push a filled-in value into the very comparator the image model must beat, and would bias the estimated incremental value of imaging toward zero. Every M1 contrast in this report is therefore computed on the 707 test patients with an observed grade, which is why the paired denominators differ between contrasts.

One pre-specified missingness indicator was dropped, and the reason is identification rather than convenience. Protocol sections 12 and 21 pre-specify an explicit indicator for each incompletely observed predictor. Both indicators are computed and written to the feature table. Neither enters a design matrix. The pain indicator is exactly aliased: the maximum knee pain score is aggregated only over rows carrying a knee-pain flag, so a score exists for precisely the 3,441 of 3,709 patients with a pre-index knee-pain record, and the any-knee-pain indicator plus the pain-missing indicator sum to 1 on every row. A Cox partial likelihood carries no intercept, so that pair spans a direction the likelihood cannot see; the ridge fit split one shared effect into two equal and opposite coefficients, so the reported log hazard ratio was half the identified effect. Dropping the aliased column returns the whole effect to the any-knee-pain term, and moves validation discrimination by less than a thousandth. Deviation D9 records that change as a hazard ratio moving from 1.43 to 1.97 under the ridge penalizer then in force; deviation D24 later changed the penalizer, and the current fit gives 1.91 (95% CI 1.14 to 3.19) for the identified term (`outputs/tables/m0_coefficients.csv`, `outputs/clinical_baseline_report.md` section 2). We report the current value and name the register's superseded one rather than choosing silently between them. That is recorded as deviation D9, and the alias is re-derived from the data on every run, so a cohort in which the two stop coinciding re-admits the indicator automatically. The grade indicator is constant by construction on the M1-eligible subset, since M1 is fitted only where the grade is observed, and a zero-variance column has no hazard ratio.

The mechanism assumption, stated plainly. We assume the two incomplete variables are missing at random conditional on the observed predictors, that is, that the probability a value is absent depends on age, sex, the radiograph-to-index interval, the comorbidity flags and the knee-pain flag, but not on the unobserved value itself once those are known.

Missing completely at random is not plausible here and we do not assume it. A maximum knee pain score is absent exactly when a patient has no pre-index knee-pain record in this health system, and whether a patient has one is a function of how often they attended and where, which is associated with age, comorbidity and the pain that brings a patient to clinic. An inferred grade is absent when no eligible bilateral frontal read exists, which is a function of the views acquired and the annotations released, not of chance. Both are record-availability mechanisms in a health-system archive, and record availability is associated with the observed predictors.

Missing not at random cannot be excluded and we say so. A patient in severe pain who is managed elsewhere leaves no score in this archive, and their score would have been high had it been recorded, which is the direction a constant fill of 7.0 understates. Nothing in these data separates that mechanism from missingness at random, because the values that would settle it are the missing ones. **The assumption is untestable in this dataset.** We state it, we hold the amount of missingness low, and we check what it is worth with a complete-case analysis rather than claiming the assumption has been verified.

Complete-case sensitivity analysis. A complete-case fit of the clinical comparator was pre-specified under protocol section 24 and was run. It restricts the fit to patients whose maximum pain score and inferred grade are both observed, so the row set is complete across every clinical variable in the feature table, and it re-tunes the ridge penalizer over the same pre-specified grid under the same selection criterion as the primary fit. It holds 2,332 training and 325 validation patients carrying 49 validation events, against 2,597 and 371 with 54 events in the primary fit. On the validation split it gives a Harrell concordance of 0.572 and a 5-year censoring-weighted AUROC of 0.658, against 0.601 (95% CI 0.526 to 0.676) and 0.698 (0.611 to

https://doi.org/10.3390/jimaging12100474

0.782) for the primary fit (`outputs/clinical_baseline_report.md`, sections 1 and 8). Both complete-case values sit inside the primary estimate's own interval on a smaller sample, so the comparator's performance is not an artifact of the imputation. The any-knee-pain indicator is constant on the complete-case subset by construction and is dropped there. The analysis was run on the validation split alone and the locked test split was not read for it. The image arms take no clinical input except in the fusion arms, where what they receive is the frozen standardized comparator vector, so the same imputation and the same sensitivity analysis govern them.

S11. ConvNeXt-Tiny hyperparameter sweep (analysis added at revision)

What is being answered. Deviation D28 abandoned the pre-specified ConvNeXt-Tiny encoder after it failed to fit the training data under one configuration. Reviewers 1, 2 and 3 all challenge that decision, and Reviewer 2 puts it exactly: no sufficient technical justification was given for abandoning a modern architecture on the evidence of a single hyperparameter setting. This section specifies the sweep that answers it. The sweep is post hoc and exploratory, and no estimate reported in the main text depends on its outcome.

Why the failure is plausible on a hyperparameter argument rather than an architectural one, and why that has to be tested. ConvNeXt differs from DenseNet in the ways that make optimization brittle. It replaces batch normalization with layer normalization, ReLU with GELU, and dense connectivity with large-kernel depthwise convolutions, so it carries no batch statistics to absorb a mis-scaled learning rate, and its loss surface is far more sensitive to the learning rate and to the schedule than DenseNet121's. Its published ImageNet recipe, with a drop-path rate of 0.1, heavy augmentation and a long warmup, is calibrated for 1.28 million images. This cohort holds 2,968 development patients with 427 events, a rare event on a small sample, and the augmentation here is deliberately capped so that joint-space geometry is not deformed. A recipe tuned for the first regime is not evidence about the second. D28 tested one point in that space and drew a conclusion about the architecture from it.

Staged design. The full factorial over the axes below is 96 cells, being 4 encoder learning rates crossed with 3 layer-wise decay settings, 2 warmup lengths, 2 drop-path rates and 2 input resolutions, which is not a defensible use of compute for a robustness architecture, so the sweep runs in two stages. The grid is frozen in code (`src/sweep_convnext.py`) rather than in a configuration file, so a configuration edit cannot quietly redefine what this section calls the sweep.

Stage 1, screening. Twelve configurations, one seed each, 10 epochs. The axes are the encoder learning rate at {3e-5, 1e-4, 3e-4, 1e-3} crossed with layer-wise learning-rate decay at {none, 0.75, 0.9}, with drop-path fixed at 0.0. Ten epochs suffice because the failure signature D28 records appears by epoch 3 to 5: the training loss sits where it started rather than falling away from it. The sweep also writes a percentage fall from `train_nll_base` on every row of both stages, and that quantity is easy to misread, so it is defined here. `train_nll_base` is the **marginal-hazard null model**: the discrete-time negative log-likelihood obtained by giving every patient the training split's own base-rate interval hazards, each interval's events over its patients at risk, computed from no image at all. It is one constant, identical on every row of both stages, and it is neither a per-cell first-epoch value nor either published arm's training negative log-likelihood. A fall from it is therefore a fall from the null and not a gain on the abandoned run, and it is not the comparator this section judges the sweep on; the comparator is the pair of published anchors fixed below, under "The comparator is fixed before the sweep runs".

https://doi.org/10.3390/jimaging12100474

Stage 2, confirmation, as executed. The sweep carried 1 screening configuration into confirmation, the best-ranked cell of Stage 1, and ran it on the five pre-specified seeds, moving one factor at a time from a reference cell at warmup 0, drop-path 0.0 and 512-pixel input: warmup raised to 5 epochs, drop-path raised to 0.1, and input resolution lowered to 224 pixels, each read against that shared reference and with the other two axes held at their reference levels. That is four cells. **This is narrower than the design deviation D41 registered**, which was the best two configurations crossed with all eight combinations of the three axes, sixteen cells; the run was `--design ofat --top-k 1`, and deviation D48 records the narrowing and what it costs. Two things are given up and neither is recoverable from the artifact: every interaction between the three axes is unmeasured, because each axis is read only at the reference levels of the other two, and the second-ranked screening configuration was dropped at that point and appears in no Stage 2 row, so no five-seed confirmation of it exists. The budget is 17 epochs per cell, not the sweep module's default. The sweep holds no validation split and therefore cannot early stop, so at the module default a swept cell would receive more than twice the descent of the early-stopped arms it is contrasted with and any part of a difference could be budget rather than the axes being swept; the budget is instead matched to the longest the DenseNet121 reference arm itself ran before early stopping, and deviation D46 records the re-budgeting. The 224-pixel cell tests the hypothesis D28 asserted and never tested, that part of the failure is a mismatch between the 512-pixel crop and the resolution the ImageNet pretraining was performed at.

Selection reads no split. Every configuration is ranked on **training** negative log-likelihood, which is the exact quantity D28 used to abandon the architecture. No validation metric and no test metric enters the ranking or the choice of what to carry into Stage 2. The sweep therefore costs no read of any held-out split, the locked test split remains at its single recorded read, and the 54-event validation split is not used for tuning. Training negative log-likelihood is summarized the same way it is summarized everywhere else in this report: the mean over seeds of each seed's minimum across epochs.

The comparator is fixed before the sweep runs. The sweep is judged against the two figures the retrained ladder already produced, both from 'outputs/tables/train_history.csv': 0.607 for the ConvNeXt-Tiny frontal arm and 0.371 for DenseNet121 on identical frontal crops, each the mean over five seeds of that seed's minimum training negative log-likelihood. Each is read against its own arm's mean first-epoch training negative log-likelihood in the same table, 0.611804 for ConvNeXt-Tiny and 0.612705 for DenseNet121. Those two are per-arm quantities recomputed from the training history, and they are **not** 'train_nll_base', which is the single null-model constant defined above and shared by every sweep row; the values sit close together and mean different things, and no statement in this report may put one in place of the other. On those first-epoch anchors the two arms fell 0.76% and 39.4% from where they started. The corresponding minimum validation negative log-likelihoods are 0.618 and 0.581, and they are reported for description only; both are minima on the split the checkpoint is selected on, so neither is an out-of-sample quantity.

What each outcome obliges us to say, declared before the result existed. If no configuration brings ConvNeXt-Tiny materially below 0.607, the abandonment recorded in D28 stands, and the reported ladder is unchanged. If a configuration does bring it down, the abandonment was premature, the register entry is corrected, and the encoder contrast already in this report is reported for what it then is, a tuning failure rather than an architectural finding. The manuscript already reads that contrast as a fitting failure, so the second outcome sharpens an existing statement rather than reversing one. Either way no model is refitted, re-

ensembled or re-recalibrated for the primary analysis, and the primary estimates do not move. Both branches were on the record before the first cell was trained. The sweep has since been run, on the executed design described above: it records that the abandonment was premature, deviation D41 discharges the commitment made here on that finding, and Supplementary Results S10 reports the result.

S12. Second architecture that converges: ResNet-50

What is being answered. Reviewer 1 asks for at least one independent architecture that converges stably, so that the result is not specific to DenseNet121. The reported ladder contains one alternative encoder and it failed, which cannot separate the claim that DenseNet121 works from the claim that this pipeline works. A third architecture settles that.

Design. ResNet-50, initialized from ImageNet weights, on the frontal view, under the identical training contract as the DenseNet121 frontal arm: the same crops, labels and locked splits, the five pre-specified seeds, the 40-epoch budget, early stopping on validation loss with a patience of 8 epochs, and the regularization, discriminative learning rates and gradual unfreezing given in Supplementary Methods S5. `resnet50` is already an admitted architecture name in the trainer (`src/train_model.py:205`), so the arm needs a configuration block and no code change, and it inherits the same training-contract hash assertion as every other arm.

What is read, and what is not. We report the arm's training negative log-likelihood and its validation discrimination, and the validation figures carry an explicit caveat because they are optimistic by construction: early stopping selects the retained checkpoint on the validation split and the same split is then scored, which is why `outputs/tables/val_comparisons.csv` marks every validation AUROC comparison among the image arms `SUPPRESSED -- severe_overfit`. The ResNet-50 arm trains under the identical contract, so its validation figure carries the identical defect. It is reported as a convergence check on a second architecture and never as an estimate of performance, and no validation AUROC from this arm may be quoted without that caveat attached. **The locked test split is not read for this arm.** It is a convergence check on a second family of architectures, not a fourth entry in the performance table, and scoring it on the test split would require a second sealed read, which the protocol does not permit.

S13. Multi-view fusion ablation

What is being answered. Reviewer 1 asks whether masked attention pooling is superior to mean pooling, max pooling or feature concatenation. The pre-specified aggregator is the gated attention pool (protocol section 14) and it remains the default everywhere, so an unspecified aggregator reproduces the trained ladder exactly.

The four aggregators. The trainer exposes four set aggregators over the crops a patient contributed. Attention, mean and max declare the same parameters at the same shapes, so a checkpoint trained under attention loads into any of the three and the swap is an inference-time re-scoring of a frozen network rather than a retrain; mean and max simply never read the attention parameters. Concatenation is different in kind. It keys one fixed block per view rather than concatenating padded slots in an arbitrary stored order, averages duplicate crops of one view inside that view's own block, and leaves an absent view's block at exactly zero, so it widens the risk head's input from the feature dimension to the number of views times the feature dimension.

What runs where, and what has run. Mean and max are an inference-time swap on the frozen five-seed ensemble, which is the same class of frozen-network re-scoring as the view

withholding already reported in Supplementary Table S4, and it falls under the same post hoc declaration. Those two swaps have been run, on both image arms and on both rosters. Concatenation changes the head's input width, cannot load a trained checkpoint, and was retrained; it was fitted and scored on the **validation** split alone, and the locked test split was not read for it. **Both concatenation arms completed on 2026-09-14**; the defect recorded in deviation D47 was fixed before this run, and deviation D43 records the analysis as complete. Supplementary Table S10 Panel C reports both arms without a paired contrast against attention pooling, which cannot be estimated for the reasons given in the table's note.

Caveat one: an inference-time swap does not measure what a mean-pooled model would have achieved. The encoder and the head were trained under attention. Swapping the aggregator at inference measures how much the learned representation depends on attention weighting. A model trained under mean pooling from the start would have learned different features, so a fall in performance under mean pooling is evidence that the trained network uses its attention weights, not evidence that attention is the better architecture. Only the retrained concatenation arm answers the architectural question, and it answers it on the validation split. That validation estimate carries the same defect as every validation estimate in this report: the checkpoint is selected on the split it is then scored on, and `outputs/tables/val_comparisons.csv` marks the attention-pooled all-views arm's own validation AUROC comparison `SUPPRESSED -- severe_overfit` for exactly that reason. Any validation AUROC from that arm therefore carries that caveat, as the attention-pooled arm's own validation AUROC does, and neither is comparable to a test-split figure. We state this in place of the comparison a reader might otherwise assume, rather than putting it in a footnote. The concatenation retrain completed, but it answers the architectural question only on the validation split, where both sides of the comparison carry the same optimism and no paired contrast could be estimated.

Caveat two: a swap cannot change the prediction of a patient with one crop. Pooling runs over the crops a patient contributed, so on a patient with exactly one crop every aggregator returns that crop's own feature vector and all four are the identity. Of the 740 test patients with a prepared crop, 373 contributed more than one crop and 367 contributed exactly one, so a swap can change the predictions of those 373 and of none of the other 367. The paired view-withholding roster used elsewhere in this report, the 315 patients holding both a frontal crop and at least one non-frontal crop, is a subset of those 373. Separately, 419 of the 740 patients (56.6%) contributed a frontal radiograph and no other view, which is the reason the whole-split contrast between the single-view and the multi-view arms is flat whatever the aggregator. The 367 single-crop patients still count in a whole-roster difference between aggregators. A swap moves the multi-crop patients' risks relative to theirs, so it can reorder patients across the two groups. **The ablation reports both rosters, on every row.** Mean and max pooling were re-scored at inference on the frozen ensemble, and the run writes each contrast twice: once on the 373 multi-view patients, which is Supplementary Table S10 Panel B, and once on the whole 740-patient scored roster, which is Panel A. Both come from the same predictions, against the same attention baseline, through the same paired bootstrap at the same replicate count, so a Panel A number and a Panel B number differ by which patients are in them and by nothing else. Panel A's attention rows are the frozen arms' already-published whole-roster estimates. Each figure that is reported is labelled with its own denominator, and we do not present a whole-split difference as the size of the aggregator's effect.

https://doi.org/10.3390/jimaging12100474

S14. Outcome-record audit and index-side verification

Requirement. Protocol section 23(ii) requires two independent reviewers to adjudicate at least 200 sampled outcome records against the CPT chronology, with raw agreement and Cohen kappa reported, disagreements resolved by consensus or a third reviewer, and the review expanded if any critical-error rate exceeds 2 percent. The decision rule below names the single rate that comparison is made on, and the sample it is estimated in. The sample stayed unadjudicated until this revision, which kept deviation D22 open. That review is the one Reviewer 3 asked for, since contralateral arthroplasty within 5 years is the study's outcome and the label is derived from procedure records rather than read by a human.

Sample. The pre-specified 200-record sample was kept and a 60-record test-split extension was added, giving 260 records. The pre-specified sample holds 200 patients, 144 training, 15 validation and 41 test, drawn as 100 patients with an algorithmically derived 5-year event and 100 without, with the index side coded on the record for 120 of them and recovered by rule for 80, and each row carrying its own decodable pre-index and post-index CPT chronology. Running that sample unchanged is what closed D22 as written; re-drawing it after the reviews arrived would abandon a pre-specified draw and would deserve the criticism it would attract. The extension adds 60 further test-split records, so 101 of the 260 come from the locked test split, which is what turns the test-split estimate from a footnote into an interval a reader can use.

Blinding, independence and adjudication. Reviewers adjudicated the outcome from the full CPT chronology with the algorithmically derived label hidden, and the comparison against that label was made afterwards. Two reviewers scored every record independently, without conferring and without comparing sheets, exactly as the section 23(i) image review was run. The two reviewers agreed on every answer, so none needed adjudication by the orthopaedic surgeon. The reference standard, adjudicated or agreed, and the raw inter-reviewer agreement are distinct quantities and are reported separately. `NOT_ASSESSABLE` was a valid answer. A reviewer's `NOT_ASSESSABLE` took that record out of the agreement and kappa denominators for that question, and those records are counted separately; a `NOT_ASSESSABLE` reference answer, adjudicated or agreed, took the record out of the predictive-value and error-rate denominators. It meant the record could not settle the question rather than that the call was difficult.

The decision rule was pre-declared in writing before scoring began. Protocol section 23 sets a 2 percent critical-error threshold, the same value section 23(i) applies to the image audit, and the register binds the consequence. The adjudication must not be used to select, tune or threshold any model. The rate compared against the threshold is the design-weighted one, $[(1 - \text{PPV})\,N+ + (1 - \text{NPV})\,N-] / (N+ + N-)$, with each predictive value estimated inside its own stratum and N+ and N- the cohort counts of algorithm-positive and algorithm-negative patients; `score_outcome_audit` emits it as `cohort_weighted_rate`. **That formula fits more than one sample and more than one set of cohort counts, so the rule names the single pair that binds: the rate estimated in the pre-specified 200-record sample and weighted to the locked cohort, N+ 533 and N- 3,176**, which the scorer labels `prespecified` at `locked_cohort`. Two reasons fix it there. The 60-record extension is drawn from the test split rather than cohort-wide, so the pooled 260 records are a simple random sample of no population and cannot carry a gate; and the consequence declared here reaches all 3,709 patients, which only a locked-cohort weighting licenses. The scorer still computes the rate and the verdict for every other sample and set of counts it can reach, including the pooled 260 and the test-split counts of 106 and 635, and it marks each of those in its own output as reported rather than gating.

https://doi.org/10.3390/jimaging12100474

We report them beside the gate whatever they say, and none of them trips the rule or moves the scorer's exit status, because a rule that gains triggers after it is written is not pre-declared. The naive in-sample rate is not the quantity either and is never compared to the threshold, because a 100 to 100 stratified draw from a cohort that is 14.37 percent events samples events at 18.76 percent and non-events at 3.15 percent, over-sampling events 5.96-fold, so the in-sample rate overstates the cohort label-error rate by about that factor. A design-weighted rate above 2 percent in that pre-specified sample re-opens the cohort lock and rebuilds the labels for every patient in the cohort, which is expensive and is accepted as the price of a threshold that means something. If the gate cannot be computed at all, the scorer records that state rather than a pass, and the audit paragraph in Supplementary Results S13 stays unwritten. Both the threshold and the consequence were fixed before the first record was scored, because a threshold chosen after the result is not a threshold. The decision rule's text was fixed on 4 September 2026; it was signed on 15 September 2026, after the two reviewers had scored (12 and 13 September 2026) and before the scorer was run on 2026-09-15.

Statistics. Every proportion carries a Wilson 95 percent confidence interval, which is the method the section 7 laterality audit reports and the method that behaves correctly near the boundary where a normal approximation does not. We report three things. First, inter-reviewer raw agreement per item before adjudication, with its interval, and Cohen kappa per item. Second, the derived label against the adjudicated reference standard. The 100 to 100 stratified draw fixes what is directly estimable there: the positive predictive value inside the algorithm-positive stratum and the negative predictive value inside the algorithm-negative stratum, each with a Wilson interval. Sensitivity and specificity are not directly estimable from this sample, because its event fraction is 50 percent by design, so they are backed out from the two predictive values at a stated event fraction rather than read off the sample. Supplementary Results S13 quotes them at the test split's event fraction of 106 of 741 and names that fraction in the sentence, so a reader can see which population they describe; like every rate other than the gate, they are reported and are compared to no threshold. Third, among confirmed events, the median absolute difference in days between the adjudicated and the derived event date and the number of events whose day-1826 classification changes. Index-side agreement is reported with a Wilson interval and **separately for the test subset**, which is the quantity Reviewer 3 asks for.

Test-split laterality cannot be verified by image review, and the reason is structural. Reviewer 3 asks that the test cases be verified directly. On the index side, image review cannot do it. Every pre-index film shows two native knees; preprocessing mirrors right knees to a common orientation, so all crops read as a left knee; and the border mask removes any burned-in side marker. The reviewer instructions issued with the image review state the consequence in those terms: "right knees are mirrored to look like left ones and the border mask removes any L/R marker, so a crop is anatomically identical whether the correct or the wrong half was taken. Any answer there would be invented." For that reason 82 of the 400 panels in the section 23(i) review arrived pre-marked not assessable on laterality and were scored on the other five items alone. Those 82 are exactly the test-split panels: the audit workbook shows a full film beside the crop on 318 of the 400 panels and on none of the 82. Test patients have finished crops and nothing else, because the source DICOM archive is no longer held and cannot be regenerated, so that set can never grow. Asking a reviewer to confirm half-selection from a crop asks an undecidable question that would be answered in good faith and would measure nothing.

https://doi.org/10.3390/jimaging12100474

What replaces it, and it does reach the test split. Index side is recoverable from the record rather than from the image. The deterministic rule used a same-date image laterality annotation, a laterality-specific ICD-10 M17 diagnosis and a same-day study description, all dated on or before the index procedure, and a human reading the full CPT chronology can confirm or contradict that rule with no image at all. The outcome audit carried exactly that check: every sampled record displayed its chronology, each reviewer recorded the index side independently of the derived value, and the 60-record extension put 101 test-split patients into the sample. That is the answer available for the test split, it is record-based rather than imaging-based, and Supplementary Results S13 reports it as such. We do not describe half-selection on the test split as visually verified, and no statement in this report may say that laterality was verified on 400 images.

Supplementary Results

This file holds the results detail relocated from the Results when the body was compressed for length. Sections S1 to S9 follow the order of the Results and nothing in them is new; every value in those nine sections appeared in an earlier version of the Results and is reproduced without change. Sections S10 to S13 report analyses added at revision. Every value in them is substituted from the run that produced it. No value in those four sections has been estimated, carried over from another arm, or filled in by hand.

S1. Cohort, crops and missingness

Of 83,011 patients in the source archive, 3,709 entered the landmark cohort (Supplementary Figure S2). Missingness was confined to the maximum pain score and the inferred Kellgren-Lawrence grade, at 7.0% and 3.7% of development patients; Table 1's missingness column spans all 3,709 landmark-cohort patients, where the two are 7.2% and 3.9%.

Preprocessing wrote 6,071 crops for 3,706 patients, of which 1,216 belonged to 740 of the 741 test patients. Nineteen images were excluded by the protocol's masking gate between selection and shard writing, 17 in development and 2 in the test split, and none was excluded for a localization failure. Across the finished set the masked fraction of the crop had a mean of 23.09%, a 90th percentile of 23.64% and a maximum of 44.75% against the fixed border band of 22.75%, and 1,572 of 6,071 crops carried masked pixels beyond that band, from out-of-bounds padding, from accepted marker blobs, or from both. Joint localization used the deterministic centred box on 5,985 of 6,071 crops and the intensity-profile refinement on 86, with a mean crop confidence of 0.9957. Half-selection from a bilateral film was required on 4,069 of the 6,071 crops, and the source dataset's horizontal-flip annotation altered which half was taken on 287 of them, 63 of those in the test split.

Figure 1 discloses that the crop it draws carries a surviving burned-in marker, and Figure 2 discloses the same of its false-negative case. Before masking the marker rate ranged from 15.8% of frontal films to 52.7% of lateral films, and after masking the test-crop rate was 9.2% frontal, 43.0% lateral and 21.6% sunrise; the residual band values reach a maximum of 48 of 255 and touch 0.049% of band pixels.

Counted by patient over the 740 test patients with a prepared crop, 419 (56.6%) contributed a frontal radiograph and nothing else, 317 (42.8%) at least one lateral view and 37 (5.0%) a sunrise view; 316 hold two or more distinct views, 315 of them hold both a frontal and a non-frontal crop, and 321 contribute a non-frontal crop. Counted by image, the test split

holds 858 frontal crops from 734 patients, 321 lateral crops and 37 sunrise crops, a mean of 1.64 crops per patient, and 373 patients hold more than one crop of any kind.

S2. Joint localization within the crop

Because the crop is a square region centred on the collimated field, the tibiofemoral joint is central by construction, and we measured how well that holds rather than assuming it. A model-free matched-filter joint-line detector run over all 858 frontal test crops placed the detected joint line inside the rows of the predefined joint region on 91.0% of images, with a median joint-line row of 284.5 (interquartile range 224 to 334) in a 512-pixel crop. The limb centre fell inside the columns of that region on 98.5% of images, and both landmarks fell inside on 90.1%. Horizontal centring is the looser of the two: the median limb-centre column was 240.2 (interquartile range 202 to 283) against an image centre of 256, and the fixed region covers a mean of 79.5% of the limb's own width. Every regional attribution result is therefore repeated on a per-image anatomically registered region as a sensitivity analysis.

S3. Single-view contrasts, concordance and sensitivity analysis

Both single-view contrasts were added after the locked split had been read and are reported as post-hoc (deviation D35). On their own paired sets the frontal-view model scores 0.837 against 0.675 for the clinical model (n = 734) and 0.846 against 0.732 for the clinical model with the inferred grade (n = 707). The same two contrasts on Harrell concordance give 0.134 (95% CI 0.074 to 0.191; P < 0.001) and 0.062 (0.018 to 0.107; P = 0.007). A sensitivity analysis of the second, run against the discrete-time implementation of the grade model, gave 0.105 (0.051 to 0.161) in IPCW AUROC and 0.055 (0.012 to 0.099) in Harrell concordance.

On identical frontal images the DenseNet121 frontal-view model reached 0.837 against 0.709 (0.643 to 0.775) for the ConvNeXt-Tiny robustness arm, a difference of 0.127 (0.076 to 0.176; P < 0.001). That contrast is exploratory, two encoders having been fitted and the better kept, and it reads as a fitting failure rather than an architectural finding.

S4. Attention weights across all three views

Among the 32 test patients holding all three views, the all-views image model split its attention weight 0.213 frontal, 0.458 lateral and 0.329 sunrise, and the combined model 0.150, 0.492 and 0.358 (Supplementary Table S4, Supplementary Figure S6).

S5. Convergence, early stopping and seed variability

At the retained checkpoint the four DenseNet121 image arms carried a training-to-validation negative log-likelihood gap of 0.023 to 0.052, in the same range as the 0.036 and 0.052 of the two clinical arms and separated from them by less than the third decimal place at either end, and those gaps are lower bounds because training loss is recorded with dropout and augmentation active while validation loss is a clean evaluation pass. Over the eight further epochs each run took before early stopping halted it, validation negative log-likelihood rose 0.157 to 0.360 above its own minimum, against 0.019 to 0.034 for the clinical and ConvNeXt-Tiny arms; the learning curves are given in Supplementary Figure S1. That divergence is what the patience rule exists to catch, and it means the retained checkpoint was selected on the same validation split it is then scored on, so validation metrics are optimistic. The test split took part in neither and cannot have inflated the estimates reported here. Across the five pre-specified seeds the standard deviation of the validation negative log-likelihood

ranged from 0.0007 to 0.0209, and the ensemble scored at or below its seed mean in seven of seven arms, small beside the 0.0646 between-arm spread.

The ConvNeXt-Tiny contrast reads as a fitting failure rather than an architectural finding: ConvNeXt training negative log-likelihood fell 0.001 against 0.238 for its twin.

S6. Attribution by compartment, occlusion detail, and the border band

Grad-CAM's joint fraction carried a standard deviation of 13.1 percentage points and the integrated-gradient fraction 11.6, and on the per-image anatomically registered region the joint fraction is 0.365 against a region averaging 20.7% of the image. Within the tibiofemoral joint region both attribution methods spread their mass across compartments rather than concentrating it: 18.2% and 30.5% respectively in the medial half, 19.6% and 25.3% in the lateral half, and 9.6% and 14.0% in the patellofemoral zone, against area shares of 12.5%, 12.5% and 6.25%. The peripheral background, 52.2% of the image, received 53.8% of Grad-CAM mass and 44.2% of integrated-gradient mass. The enrichment on the per-image anatomically registered region, 1.77-fold, is the mean of the per-image ratios, because the registered region's area varies from crop to crop; dividing the two cohort means instead gives 1.76-fold.

Integrated gradients assign essentially no mass to the border band, a mean of 5.6×10^{-6} (SD 3.7×10^{-5}), and exactly zero wherever the band is exactly zero, since the method multiplies the input by a gradient against a zero baseline. That is an identity rather than a finding, and it is not exactly zero across the cohort: 18 of the 230 integrated-gradient crops carry a non-zero border mass, up to 4.9×10^{-4}, which is 7.8% of them and is the same band contamination reported in the Results, whose frontal-only rate is 6.8% of 858 crops. The 11.9% reported there is the rate across all 1,216 test crops of every view and is not the comparator for a frontal-only attribution sample. Grad-CAM assigns 8.4% (SD 4.7) of its mass to the band, a 0.37-fold depletion, but its native resolution is 16 by 16, so the 31-pixel band is smaller than one cell and that figure is smeared rather than resolved. The occlusion experiment, not either attribution method, is the instrument that bears on the border.

Re-zeroing the already-blank border band left the predicted risk of 677 of 734 patients bit-identical and moved discrimination by -0.0002 (95% CI -0.0009 to 0.0000), which validates the perturbation pipeline. The pre-specified border-band-only control is withdrawn from the paper and no result from it is reported here: preprocessing already zeroes that band, so supplying the band alone hands the network a near-constant input, and any discrimination that follows measures the destruction of the image rather than a property of the model or any burned-in text (deviation D36). Occluding the medial compartment raised the cohort mean recalibrated 5-year risk from 0.148 to 0.171. Every anatomic occlusion interval crosses zero, including every compartment's change in discrimination, so these conditions establish the direction and size of a risk shift and not a ranking of compartments. One anatomic condition in Supplementary Table S3 does exclude zero, a mean-fill variant of the joint-only crop at -0.051 (95% CI -0.092 to -0.010); it is the condition most favourable to the model, its zero-fill counterpart crosses zero, and it licenses no ranking either.

Attribution passes a parameter-randomization check. Under cascading randomization, map similarity to the trained network, measured within imaged pixels, falls from +0.604 for an untouched single seed to at most +0.268, with three of the five randomization scopes at or below zero, while discrimination collapses from 0.837 to between 0.234 and 0.569. The map statistics rest on 60 sampled crops and the discrimination statistics on the arm's full 734-patient roster. The second sanity check the protocol names, re-running attribution after

https://doi.org/10.3390/jimaging12100474

randomizing the outcome labels, requires retraining and was not performed; that scope cut is recorded in deviation D36 and no result is claimed for it.

Figure 2 shows the four test-split patients at inferred Kellgren-Lawrence grade 2, one from each cell of the classification table at an operating point of 0.134 recalibrated predicted 5-year risk. Their recalibrated 5-year risks are 0.037, 0.053, 0.207 and 0.398, a 10.8-fold spread inside a single radiographic grade; on the raw model output before recalibration the same two patients are 4.24-fold apart, so the fold change is a property of the reported scale and not of the ordering. That operating point equalizes predicted positives and observed events among the 263 of 734 patients who are classifiable at 5 years, being 106 with the event by then and 157 observed event free beyond it; the other 471 are censored earlier and cannot be placed in the table, and the same threshold applied to the whole 734 flags 281 patients.

S7. Robustness and ablation strata

Across the two image arms the stratum table holds 136 cells, of which 62 carry an estimate and 74 do not, and the two reasons for a blank cell are not interchangeable. Sixty-eight of them fall below the event floor. The remaining six are undefined rather than imprecise: only 162 of the 741 test patients are observed beyond day 1825, and in each arm three strata clear the event floor while containing no such patient at all, so the horizon-specific estimator has no controls to work with. Every stratum is therefore also reported on Harrell concordance, which is horizon free (Supplementary Figure S5, Supplementary Table S2).

Under the all-crops masking definition, discrimination was 0.857 (95% CI 0.788 to 0.918) in the 471 patients whose crops carry only the fixed border band and 0.786 (0.683 to 0.879) in the 263 patients with additional marker masking, with widely overlapping intervals and no pairing between them. Under that definition more masking is largely a proxy for having a lateral film, because lateral views carry burned-in markers far more often: 212 of the 316 patients with more than one view sit above the masking floor against 51 of the 418 the source dataset's pre-preprocessing view-set label calls frontal-only, both counted on the 734-patient frontal-view roster. Restricted to the frontal crop alone, where that confounding is removed, the more-masked level holds 21 events and is suppressed. There is therefore no unconfounded two-sided comparison of masking in these data, and we do not report one.

Weight-bearing frontal films gave 0.881 (0.832 to 0.924) in 673 patients, but the 61 non-weight-bearing patients carry 5 events and are suppressed, and the weight-bearing level covers 92% of the arm's roster, so it restates the whole-split estimate. Photometric inversion, bilateral half-selection, joint-localization method and crop confidence behave the same way. Crop confidence and localization method are one partition rather than two, because a confidence below 1.0 holds if and only if the intensity-profile localizer was used, on all 6,071 images; that level holds 16 test patients and 2 events, and no partition of it can ever reach 50. The horizontal-flip annotation, which is the single unverifiable step in the pipeline, is set on 63 test images carrying 7 events in total and is reported as a count rather than as a stratum.

Under the two data-driven era schemes the 5-year estimator is undefined in the most recent stratum because no patient in it was observed for 5 years, and under the calendar scheme it rests on three such patients. There is one event among the 303 cohort patients dated through 2012, none of them in the test split. An era stratum on these data measures when the record system began capturing follow-up, not when the film was taken.

Of the 13 pre-specified equity subgroup estimates, which are computed on the primary combined model rather than on the frontal-view model, 7 fell below the 50-event floor and are suppressed. Six cleared it, from 0.804 (0.719 to 0.879) in the 322 patients whose selected

study was not frontal-only to 0.873 (0.819 to 0.920) in the 673 with a weight-bearing frontal film; every interval overlapped every other, so no group difference is established, and the equity question is deferred to a cohort large enough to answer it.

S8. Prediction within Kellgren-Lawrence strata

Under the pre-declared bin edges the 707 patients with a recorded grade divide into 144 with grades 0 to 1 carrying 3 events, 266 with grade 2 carrying 30, and 297 with grades 3 to 4 carrying 65. Within grades 3 to 4 the frontal-view model reached 0.802 (95% CI 0.716 to 0.880) and the combined model 0.841 (0.764 to 0.908); their paired difference was 0.039 (95% CI -0.025 to 0.105; P = 0.23), and that contrast is itself diluted, because 122 of those 297 patients have a frontal film only.

Among grade 2 knees observed 5-year incidence rose across tertiles of predicted risk from 4.5% (1.4 to 13.7) through 17.0% (8.7 to 31.7) to 29.3% (18.9 to 43.7), a 6.5-fold range (log-rank $P = 8.6 \times 10^{-5}$); among grade 3 to 4 knees the middle tertile is 31.5% (21.0 to 45.4).

Adding the frontal-radiograph risk score to a Cox model containing the grade improved fit by a likelihood-ratio chi-square of 65.7 on 1 degree of freedom ($P < 0.001$), and adjustment for the grade attenuated the unadjusted hazard ratio only from 3.09 to 3.01.

Within grades 3 to 4 the frontal-view model's interval is wide enough to be flagged as such by the pre-declared width criterion. In the 144 patients with grades 0 to 1, who carried 3 events between them, observed incidence by tertile was 4.0%, 2.4% and 5.9% (log-rank P = 0.92); that is an uninformative cell, not a demonstrated failure in early disease.

The Cox conclusion was unchanged when the grade entered as three categories or as a stratified baseline hazard, and Schoenfeld residual tests gave P values from 0.41 to 0.98 for every term of every specification. The combined model behaved like the frontal-view model, at a hazard ratio of 2.76 (2.16 to 3.53) per standard deviation with a likelihood-ratio chi-square of 69.6. Because the risk score is the complementary log-log of predicted risk standardized to unit variance, and the frozen recalibration is affine on exactly that scale, none of these results depends on whether the recalibration was applied. All of them are post-hoc analyses of an already-read split, and the grade they are measured against is itself model inferred, so "beyond the grade" here means beyond an automated grade rather than beyond a radiologist's reading (Supplementary Table S5, Figure 4).

S9. Calibration and decision-curve detail

Figure 4, panel A, plots the two image arms after the frozen recalibration, so the panel and the calibration slope describe the same predictions; the two frozen Cox comparators carry no recalibration and are drawn as fitted. Panel A rests on the 707 patients, carrying 98 events, that all four arms score, so the curves share one population.

Across thirds of recalibrated predicted risk from the combined model the observed 5-year incidence was 3.6% (95% CI 1.6 to 7.9), 19.8% (13.7 to 28.2) and 40.6% (32.9 to 49.3), an 11.3-fold gradient (Supplementary Figure S4). The combined model's Brier score at 1825 days, 0.141, was the lowest of any arm, and the frontal-view model's was 0.143.

In decision-curve analysis a threshold is the predicted 5-year risk at or above which a rule flags a patient. At a 20% threshold the frontal-view radiograph model flagged 200 of the 734 patients it scores, whose observed 5-year incidence was 46.5%, capturing 66 events, while the clinical model flagged 354 of 741 at 26.6% to capture 67. Net benefit exceeded treating everyone from 12% and the clinical model from 18%, at every threshold to 30%; lower thresholds also cleared zero, at 1% and at 3% to 5%, in runs that broke before 30%. Both

https://doi.org/10.3390/jimaging12100474

crossings are exploratory readings of a grid fixed after the single read (deviation D29). Net benefit is estimated by Kaplan-Meier within each flagged set, because 63.8% of test patients are censored before the horizon, and it is sensitive to calibration where discrimination is not, so the horizontal axis is a decision-rule parameter rather than a true risk. No risk-triggered intervention has been validated for this outcome, so these curves compare models rather than recommend an action.

S10. ConvNeXt-Tiny hyperparameter sweep

The sweep has been run. Every quantity reported below, and the rule that picks the row it comes from, was fixed in Supplementary Methods S11 before the run, so none of them was chosen once the result was known, and nothing here is estimated, interpolated or carried over from another arm. Two things changed after the registration, and both are registered. Stage 2 ran as a one-factor-at-a-time design on the single best-screening configuration rather than the two-configuration factorial deviation D41 laid out, which is why Supplementary Table S8 Panel B carries four swept cells and not sixteen; deviation D48 records that narrowing. The epoch budget was cut to match the arms the sweep is read against; deviation D46 records that.

Stage 1, screening. Twelve screening configurations, one seed each, 10 epochs. Minimum training negative log-likelihood ranged from 0.476 to 0.613. The count of the 12 falling below the 0.607 that the reported ConvNeXt-Tiny frontal arm reached over five seeds is 4, and the count falling below the 0.371 that DenseNet121 reached on identical frontal crops is 0. One configuration was carried into Stage 2: an encoder learning rate of 3×10^{-5} with layer-wise learning-rate decay of 0.90, at 0.476. The second-ranked cell, an encoder learning rate of 3×10^{-5} with layer-wise learning-rate decay of none at 0.489, was screened and dropped, and appears in no Stage 2 row. The sweep also writes a percentage fall from `train_nll_base` on every row. That baseline is the marginal-hazard null model, a single constant shared by every row of both stages; it is neither a per-cell first-epoch value nor either published arm's training negative log-likelihood, so a fall from it is not a gain on the abandoned run. The two counts above are the comparisons against the published arms.

Stage 2, confirmation. Over five pre-specified seeds and a fixed 17-epoch budget, with no validation pass and so no early stopping, the best configuration reached a mean minimum training negative log-likelihood of 0.274, with per-seed minima from 0.261 to 0.282, under s2_lr3e-05_llrd0.9_wu5_dp0_px512_fr3. That budget equals the longest the DenseNet121 arm itself ran before early stopping, and is well short of the sweep module's default, so a swept cell is not flattered by a longer descent than the run it is compared against (deviation D46). All three one-factor moves are made from one cell and are reported against it: the reference cell is the carried configuration at warmup 0, drop-path 0.0 and 512 pixels, and each swept cell differs from it in exactly one factor. Against that reference, adding 5 warmup epochs changed the training negative log-likelihood by -0.043 and raising drop-path from 0.0 to 0.1 changed it by +0.127. Lowering the input resolution took the same reference cell from 0.317 at 512 pixels, which is the resolution the reported ladder used, to 0.325 at 224 pixels. **The 224-pixel pair is the reference cell, not the best cell**: the best cell is the warmup move, and under a one-factor-at-a-time design it was never run at 224 pixels, so only four stage-2 cells exist in total.

What the sweep establishes. The best ConvNeXt-Tiny configuration found here differs by -0.097 from the 0.371 DenseNet121 reached on the same frontal crops. The sweep therefore shows that the abandonment recorded in deviation D28 was premature. Encoder learning rate carries the result almost alone: every cell at the lowest rate screened fell below the abandoned

run's own value, only one cell at any higher rate did, and nothing at the two highest rates came off the null. The abandoned run used the encoder learning rate in the training contract D28 tuned for DenseNet121, which the ConvNeXt-Tiny robustness arm then inherited unchanged, and the screening cell at that rate reproduces its failure. It had never improved its fit once its encoder started training: the encoder was trainable from epoch 3, and in every one of its five seeds the minimum training negative log-likelihood fell on the last epoch before the backbone thawed and rose thereafter, so the statistic D28 abandoned the architecture on was a head fitted on a still-frozen ImageNet encoder. That describes the training-NLL statistic and not every retained checkpoint: early stopping selects on validation, and one of the arm's five retained checkpoints is post-thaw. DenseNet121 on the same contract reached its minimum long after thawing. Every configuration is ranked on training negative log-likelihood alone, so no held-out split is read and the locked test split remains at its single recorded read. The sweep is post hoc and exploratory, and no estimate in the main text changes because of it.

S11. ResNet-50 arm

The arm has been trained. The run is described in Supplementary Methods S12, and every quantity reported here was fixed by this section's template before it ran.

Over five pre-specified seeds and a 40-epoch budget, the ResNet-50 frontal arm reached a mean minimum training negative log-likelihood of 0.499, with per-seed minima from 0.452 to 0.610, and a mean minimum validation negative log-likelihood of 0.591. One seed is degenerate and sets the top of that range: its retained checkpoint is its own first epoch, it stopped early, and it is what makes this arm's seed-to-seed spread on validation the widest in the ladder. The comparators on identical frontal crops are 0.371 in training and 0.581 in validation for DenseNet121, and 0.607 and 0.618 for ConvNeXt-Tiny, each the mean over five seeds of that seed's own minimum. On the validation split the arm reached a 5-year censoring-weighted AUROC of 0.803 (95% CI 0.726 to 0.876) and a Harrell concordance of 0.766. **Both validation figures are optimistic and neither is an out-of-sample estimate.** Early stopping selects the retained checkpoint on the validation split and the same split is then scored, which is why `outputs/tables/val_comparisons.csv` marks every validation AUROC comparison among the image arms `SUPPRESSED -- severe_overfit`. They are reported as a convergence check on a second architecture and for no other purpose, and neither may be quoted without that caveat. The locked test split was not read for this arm, so no test-split estimate is reported for it and none should be inferred, and no patient-level prediction file exists for it, so no paired comparison against a published arm can be constructed.

The ResNet-50 result supports the reading that the ConvNeXt-Tiny result reflects that architecture's optimization on this cohort rather than a property of the imaging pipeline or of the labels.

S12. Fusion ablation: attention against mean, max and concatenation

Every analysis in this section has now been run. The inference-time swaps were re-run under the wider output schema, which reports each contrast on the whole roster and on the multi-crop patients from the same predictions. Both concatenation arms were retrained and scored on the validation split. The reporting template for all of it was fixed in Supplementary Methods S13 before any of it ran.

Inference-time swap on the frozen ensemble, whole roster. Re-scoring the frozen five-seed all-views image ensemble (M3) under each aggregator, on the 740 test patients with a

prepared crop, gives a 5-year censoring-weighted AUROC of 0.840 (95% CI 0.787 to 0.889) under the pre-specified attention pool, 0.853 under masked mean pooling and 0.853 under masked max pooling, for paired differences against attention of +0.0133 (95% CI +0.0018 to +0.0255) and +0.0129 (+0.0019 to +0.0247). Both intervals exclude zero. The attention figure is the one the reported ladder already carries (`outputs/tables/test_metrics.csv`); the other two come from the ablation re-run, which reports the same contrast on both rosters and writes the denominator on every row.

The subset on which the aggregator can differ at all. On a patient who contributed exactly one crop every aggregator returns that crop's own feature vector, so 367 of the 740 test patients contribute an identical prediction under all four and only the other 373 can receive a different one. Restricted to those 373 patients, attention gave 0.860, mean 0.874 and max 0.873, for paired differences against attention of +0.0145 (95% CI -0.0069 to +0.0370) and +0.0138 (-0.0065 to +0.0357). Both intervals cross zero. The whole-roster AUROC above also ranks single-crop patients against multi-crop patients. A swap leaves the 367 single-crop predictions unchanged but moves the multi-crop patients' risks relative to them, so those cross-group rankings can change. This subset holds no single-crop patient, so it has no such rankings. For the all-views arm, the whole-roster intervals exclude zero and these do not.

Concatenation, retrained, on the validation split. Per-view concatenation widens the head's input and cannot reuse a trained checkpoint, so it was retrained under the identical contract and scored on the validation split alone, for both image arms. On the 371 validation patients carrying 54 events, the all-views arm (M3) reached a 5-year censoring-weighted AUROC of 0.841 (95% CI 0.765 to 0.913) against 0.833 (0.756 to 0.905) for its attention-pooled reference, and the combined arm (M4) reached 0.846 (0.771 to 0.914) against 0.835 (0.756 to 0.908). The 5-seed mean of per-seed minimum validation negative log-likelihood was 0.566 against 0.575 for M3, and 0.578 against 0.564 for M4. **All four validation AUROCs are optimistic and carry that caveat.** The checkpoint is selected on the split it is then scored on, and `outputs/tables/val_comparisons.csv` marks the attention-pooled all-views arm's own validation AUROC comparison `SUPPRESSED -- severe_overfit` for exactly that reason. The same flag suppresses every validation contrast naming the attention-pooled combined arm or either concatenation arm. Supplementary Table S10 Panel C reports no paired contrast, for the reasons given in its note. The locked test split was not read for either concatenation arm.

How to read this. The mean and max results measure how much the frozen network's prediction depends on the attention weights it was trained with. They do not measure what a mean-pooled model would have achieved, because such a model would have learned different features. Only the retrained concatenation arm compares architectures, and it does so on the validation split, where the retained checkpoint was also selected, so its estimate is optimistic in the way every validation estimate in this report is. Attention pooling was not superior to mean pooling, max pooling or concatenation on any roster or split. For the all-views arm on the whole roster (740 patients, 106 events), mean and max pooling were marginally higher, by +0.0133 (95% CI +0.0018 to +0.0255) and +0.0129 (+0.0019 to +0.0247). Either swap moves the multi-crop patients' risks relative to those of the single-crop patients, which do not change. For the combined arm on the whole roster, and for both arms on the 373 multi-crop patients, every interval includes zero (Supplementary Table S10 Panels A and B). On validation, concatenation gave an AUROC of 0.841 for the all-views arm and 0.846 for the combined arm, against 0.833 and 0.835 under attention pooling. Those figures share the optimism described above, and no paired contrast could be estimated, so they are not evidence that concatenation is better. The primary estimates do not depend on the aggregator.

S13. Outcome-record audit

Every value in this section comes from the scorer's output after the adjudication described in Supplementary Methods S14. The paragraph below was fixed as a template before any record was scored, so the reported quantities could not be chosen after the result was known.

Two expert reviewers independently adjudicated 260 sampled outcome records against the full pre-index and post-index CPT chronology, blinded to the algorithmically derived label, with no disagreement requiring adjudication. Raw agreement before adjudication was 100.0% (Cohen kappa 1.00, with no interval because agreement was perfect; for raw agreement, 95% CI 98.5 to 100.0). Against the adjudicated reference standard, pooled across all 260 records, the derived 5-year contralateral arthroplasty label had a positive predictive value of 100.0% (95% CI 97.1 to 100.0) and a negative predictive value of 100.0% (95% CI 97.1 to 100.0), corresponding to a sensitivity of 100.0% and a specificity of 100.0% at the test-split event fraction of 106 of 741. Among confirmed events the adjudicated and derived event dates differed by a median of 0 days, and 0 of 130 events changed 5-year classification. The index operative side agreed with manual adjudication in 260 of 260 sampled patients (100.0%, Wilson 95% CI 98.5 to 100.0), including 101 of 101 test-split patients (100.0%, 95% CI 96.3 to 100.0). The design-weighted label error rate of 0.00%, estimated in the pre-specified 200-record sample and weighted to the locked cohort, did not exceed the pre-specified 2% threshold, so the cohort lock was retained. That rate weights each stratum's error by the locked cohort's counts of algorithm-positive and algorithm-negative patients, 533 and 3,176, rather than by their share of the sample, which over-samples events 5.96-fold by design; it is the one rate the pre-declared rule names. The 60-record test-split extension is reported beside it and is not pooled into the threshold comparison, and the in-sample rate is not compared to the threshold either.

Records that either reviewer returned as not assessable numbered 0 for the event question, 0 for the event date and 0 for the index side. Those records left the inter-reviewer agreement and kappa denominators and are counted here rather than absorbed into an agreement rate, and a record whose reference answer, adjudicated or agreed, was not assessable left every denominator that uses the reference standard. The index-side figure on the test split answers whether the deterministic recovery rule holds on the split the reported estimates rest on; it is a record-based check on the CPT chronology and not an image review, for the reason given in Supplementary Methods S14.

The pre-specified 200 records alone, reported beside the gate and compared with no threshold, gave a positive predictive value of 100.0% (95% CI 96.3 to 100.0) and a negative predictive value of 100.0% (95% CI 96.3 to 100.0). These are the gate's own inputs; at the locked cohort's counts they imply a sensitivity of 100.0% and a specificity of 100.0%, against 100.0% and 100.0% for all 260 records at the same counts. The other five design-weighted rates, reported but not gating, ran from 0.00% to 0.00%, and none exceeded 2%. For the other two questions, raw agreement before adjudication was 100.0% for the event date (kappa 1.00, no interval) and 100.0% for the index side (kappa 1.00, no interval). Event side was not analysed: both reviewers entered the index side in the event-side column, and the contralateral side follows from the index side. Among confirmed events, the absolute difference between adjudicated and derived event dates had an interquartile range of 0 to 0 days and a maximum of 0 days. The two reviewers agreed on every answer, so none needed adjudication by the orthopaedic surgeon.

https://doi.org/10.3390/jimaging12100474

Supplementary Tables

Where a note in the main or the supplementary tables records that an analysis was post hoc, that a rule was validated only outside the locked test split, or that a procedure departed from the registered protocol, the registered deviation identifier for that claim is given in Supplementary Table S6. Protocol deviation cross-walk.

Table S1. Additional discrimination and calibration metrics for each arm.

Model	Uno C (95% CI)	IPCW AUROC 365 d (95% CI)	IPCW AUROC 730 d (95% CI)	Slope 365 / 730 / 1825 d	CITL 365 / 730 / 1825 d	Brier 365 / 730 / 1825 d
M0 clinical, frozen penalized Cox	0.635 (0.586 to 0.682)	0.666 (0.605 to 0.728)	0.666 (0.604 to 0.725)	1.25 / 1.23 / 1.28	0.005 / −0.006 / −0.001	0.090 / 0.107 / 0.158
M0d clinical, discrete-time control	0.642 (0.594 to 0.692)	0.658 (0.596 to 0.722)	0.654 (0.592 to 0.716)	1.30 / 1.50 / 0.94	0.059 / 0.010 / 0.169	0.095 / 0.108 / 0.199
M1 clinical plus inferred KLG, frozen penalized Cox	0.709 (0.655 to 0.760)	0.741 (0.675 to 0.801)	0.751 (0.689 to 0.813)	1.24 / 1.34 / 1.03	0.007 / −0.005 / 0.009	0.082 / 0.096 / 0.150
M1 clinical plus inferred KLG, discrete-time control	0.713 (0.660 to 0.765)	0.741 (0.676 to 0.800)	0.748 (0.687 to 0.810)	1.25 / 1.57 / 1.08	0.030 / 0.002 / 0.120	0.084 / 0.097 / 0.174
M2 radiograph, frontal view	0.763 (0.718 to 0.802)	0.809 (0.758 to 0.856)	0.831 (0.782 to 0.875)	0.79 / 1.09 / 1.01	0.054 / 0.017 / 0.056	0.088 / 0.094 / 0.143
M3 radiograph, all views	0.756 (0.708 to 0.800)	0.808 (0.750 to 0.860)	0.839 (0.791 to 0.884)	0.83 / 1.21 / 0.98	0.026 / −0.005 / 0.090	0.081 / 0.088 / 0.149
M4 combined, frontal view only	0.758 (0.712 to 0.799)	0.810 (0.760 to 0.858)	0.830 (0.782 to 0.876)	0.74 / 1.09 / 0.98	0.062 / 0.031 / 0.082	0.090 / 0.096 / 0.155
M4 combined, all views, primary arm	0.765 (0.720 to 0.810)	0.815 (0.761 to 0.866)	0.846 (0.799 to 0.889)	0.70 / 1.10 / 1.02	0.044 / 0.004 / 0.070	0.084 / 0.089 / 0.141
R1 radiograph, frontal view, ConvNeXt-Tiny	0.677 (0.627 to 0.728)	0.739 (0.675 to 0.797)	0.758 (0.702 to 0.811)	0.75 / 1.19 / 1.03	0.102 / 0.124 / 0.198	0.104 / 0.127 / 0.215

Denominators are the same as in Table 3. CITL is calibration-in-the-large. Slope, calibration-in-the-large and Brier score for the image arms are computed after the frozen horizon-specific recalibration; the two frozen Cox arms carry none. Intervals are 95% percentile intervals from the same paired bootstrap.

Table S2. Discrimination of the frontal-view model within strata of image acquisition and image quality.

Stratum	Level	Scope	Patients / events / 5-year controls	IPCW AUROC at 1825 days (95% CI)	Harrell C (95% CI)
Frontal radiograph technique	Weight bearing	not applicable	673 / 101 / 133	0.881 (0.832 to 0.924)	0.791 (0.749 to 0.829)

Frontal radiograph technique	Non weight bearing	not applicable	61 / 5 / 24	suppressed, < 50 events	suppressed, < 50 events
Imaging views	Frontal only, by view-set label	not applicable	418 / 37 / 94	suppressed, < 50 events	suppressed, < 50 events
Imaging views	Multiple views, by view-set label	not applicable	316 / 69 / 63	0.767 (0.678 to 0.853)	0.716 (0.657 to 0.768)
Index laterality source	Coded	not applicable	369 / 76 / 0	not estimable, no 5-year control	0.728 (0.669 to 0.781)
Index laterality source	Recovered	not applicable	365 / 30 / 157	suppressed, < 50 events	suppressed, < 50 events
Masking	Border-band masking only (no residual-marker masking)	all crops	471 / 55 / 107	0.857 (0.788 to 0.918)	0.802 (0.744 to 0.856)
Masking	Border-band plus residual-marker masking	all crops	263 / 51 / 50	0.786 (0.683 to 0.879)	0.727 (0.660 to 0.788)
Masking	Border-band masking only (no residual-marker masking)	frontal crop only	619 / 85 / 137	0.827 (0.767 to 0.886)	0.787 (0.740 to 0.829)
Masking	Border-band plus residual-marker masking	frontal crop only	115 / 21 / 20	suppressed, < 50 events	suppressed, < 50 events
Crop confidence and localization method	Confidence 1.0, centred box	all crops	718 / 104 / 155	0.836 (0.782 to 0.887)	0.780 (0.739 to 0.818)
Crop confidence and localization method	Confidence below 1.0, intensity profile	all crops	16 / 2 / 2	suppressed, < 50 events	suppressed, < 50 events
Photometric inversion	No inversion	all crops	588 / 90 / 96	0.786 (0.715 to 0.856)	0.763 (0.717 to 0.805)
Photometric inversion	Inversion applied	all crops	146 / 16 / 61	suppressed, < 50 events	suppressed, < 50 events
Bilateral half selection	No half selection	all crops	27 / 8 / 6	suppressed, < 50 events	suppressed, < 50 events
Bilateral half selection	Half selection applied	all crops	707 / 98 / 151	0.846 (0.792 to 0.896)	0.787 (0.744 to 0.824)
Acquisition era, calendar spans	2007 to 2011	not applicable	34 / 0 / 33	suppressed, < 50 events	suppressed, < 50 events
Acquisition era, calendar spans	2012 to 2016	not applicable	217 / 12 / 121	suppressed, < 50 events	suppressed, < 50 events
Acquisition era, calendar spans	2017 to 2021	not applicable	483 / 94 / 3	0.857 (0.619 to 1.000)	0.743 (0.692 to 0.790)
Acquisition era, development tertiles	2015 and earlier	not applicable	167 / 6 / 122	suppressed, < 50 events	suppressed, < 50 events

Acquisition era, development tertiles	2016 to 2018	not applicable	277 / 49 / 35	suppressed, < 50 events	suppressed, < 50 events
Acquisition era, development tertiles	2019 and later	not applicable	290 / 51 / 0	not estimable, no 5-year control	0.738 (0.664 to 0.804)
Acquisition era, development median	2017 and earlier	not applicable	337 / 34 / 157	suppressed, < 50 events	suppressed, < 50 events
Acquisition era, development median	2018 and later	not applicable	397 / 72 / 0	not estimable, no 5-year control	0.732 (0.673 to 0.787)

Strata that could not be constructed at all

Stratum	Reason
Equipment or device model	The source dataset released no equipment tag. Manufacturer model, device serial number and station name are absent from every delivered table.
Manufacturer	No manufacturer tag was released, and the value is not recoverable, because the 34.85 GB source DICOM archive is no longer held and the retained artifacts are 512 by 512 crops with the border band zeroed.
Acquisition site or institution	No institution or department tag was released and no site identifier appears in any delivered table. Site cannot be approximated from de-identified patient surrogates.
Horizontal-flip correction	Described rather than charted. The flag is set on 63 test images, one per patient, all frontal, carrying 7 events in total, and 7 events cannot clear the 50-event floor under any partition.

Every level is a mask over predictions that already existed, so no stratum required a further read of the locked split, and all of them are post-hoc. The marginal estimates for this arm are an IPCW AUROC of 0.837 and a Harrell concordance of 0.779. A blank cell has one of two distinct meanings. “Suppressed” means the level carries fewer than the 50 events the analysis plan requires. “Not estimable” means something stronger: the 5-year IPCW AUROC compares patients with an event by the horizon against patients observed event free beyond it, and only 162 of the 741 test patients reach that day, so a level can clear the event floor and still hold no such control at all. Those cells are undefined, not imprecise, and Harrell concordance is reported throughout because it is horizon free and survives where the horizon-specific estimator does not. Crop confidence and joint-localization method are one partition rather than two, because a confidence below 1.0 holds if and only if the intensity-profile localizer was used, on all 6,071 preprocessed images. Both masking levels record how much masking a patient’s own crops received, so neither is the withdrawn border-band-only leakage control of Supplementary Table S3, which supplied the band alone to the network. The masking family is the only one in which both levels are estimable, and only under the all-crops definition, where “more masking” is largely a proxy for “has a lateral film”: 212 of the 316 patients with more than one view sit above the masking floor against 51 of the 418 the source dataset’s pre-preprocessing view-set label calls frontal-only. Both counts are on the 734-patient frontal-view roster, and that 418 is not the 419 crop-based frontal-only count reported for the 740 test patients with a prepared crop; the two classify different things and neither is substituted for the other. Under the unconfounded frontal-crop-only definition the more-masked level is suppressed, so no unconfounded two-sided comparison of masking exists in these data. Every acquisition-era row carries two further caveats: the de-identified

study date carries a per-patient random shift whose cross-patient comparability has never been confirmed in writing, and era is nearly collinear with follow-up and record availability, since median follow-up on this split falls from 1,826 days in 2007 to 2011 to 430 days in 2017 to 2021 while the share observed through 5 years falls from 97.2% to 1.0%. The one estimated era AUROC rests on 3 controls and is reported with that count rather than read as an acquisition effect. The companion table for the combined model is available with the analysis outputs.

Table S3. Attribution mass, regional occlusion and leakage controls for the frontal-view model.

Panel A. Share of absolute attribution mass by region

Region	Share of image area	Grad-CAM, mean (SD)	Enrichment	Integrated gradients, mean (SD)	Enrichment
Tibiofemoral joint region	25.0%	0.378 (0.131)	1.51-fold	0.558 (0.116)	2.23-fold
Masked border band, 31 px	22.75%	0.084 (0.047)	0.37-fold	5.6×10^{-6} (3.7×10^{-5})	0.00002-fold
Peripheral background	52.2%	0.538 (0.104)	1.03-fold	0.442 (0.116)	0.85-fold
Medial half of the joint region	12.5%	0.182 (0.105)	1.45-fold	0.305 (0.090)	2.44-fold
Lateral half of the joint region	12.5%	0.196 (0.096)	1.57-fold	0.253 (0.108)	2.02-fold
Patellofemoral zone	6.25%	0.096 (0.063)	1.54-fold	0.140 (0.082)	2.24-fold

Panel B. Discrimination and predicted risk after perturbing the image

Condition	IPCW AUROC at 1825 days	Change in AUROC (95% CI)	Mean recalibrated 5-year risk	Change in mean risk
Unperturbed baseline	0.837	reference	0.148	reference
Medial compartment occluded	0.827	−0.010 (−0.039 to 0.019)	0.171	+0.023
Lateral compartment occluded	0.819	−0.017 (−0.038 to 0.002)	0.142	−0.006
Patellofemoral zone occluded	0.826	−0.010 (−0.024 to 0.003)	0.145	−0.003
Whole joint region occluded	0.800	−0.037 (−0.079 to 0.004)	0.138	−0.010
Joint region supplied alone	0.802	−0.034 (−0.072 to 0.004)	0.150	+0.002
Whole joint region occluded, mean fill	0.804	−0.033 (−0.073 to 0.008)	0.144	−0.004
Joint region supplied alone, mean fill	0.786	−0.051 (−0.092 to −0.010)	0.130	−0.017
Residual marker blobs blanked	0.836	−0.00096 (−0.00230 to −0.00008)	0.148	+0.000006
Masked band widened to 62 px	0.833	−0.0038 (−0.0180 to 0.0115)	0.155	+0.008
Masked band widened to 93 px	0.821	−0.0154 (−0.0414 to 0.0102)	0.155	+0.007

Already-blank band re-zeroed (pipeline check)	0.836	−0.0002 (−0.0009 to 0.0000)	0.148	+0.000012

Attribution was computed for the frontal-view model on the locked test split and averaged over the five pre-specified seeds, with respect to the predicted 5-year risk. The two methods rest on different samples and the denominators are not interchangeable: gradient-weighted class activation mapping was computed at the encoder's final normalisation layer on all 858 frontal test crops from 734 patients, and integrated gradients at pixel resolution with 32 midpoint steps and a zero baseline on 230 crops from 200 patients. Enrichment is the mean share divided by the region's share of the image. The joint region is the central half of the 512-pixel crop in each dimension; crops are mirrored so that every knee reads as a left knee under the radiological convention, so the medial aspect is the image-left half of that region. Integrated gradients assign essentially no mass to the border band, and exactly none wherever the band is exactly zero, which follows from the method's zero baseline rather than from any property of the model; 18 of the 230 crops carry a non-zero border mass, up to 0.00049. Grad-CAM's native resolution is 16 by 16, so the 31-pixel band is smaller than one cell and its border share is smeared rather than resolved.

Panel B re-scores the frozen five-seed ensemble on 734 patients carrying 106 events. Occluded pixels are set to zero, which is what the preprocessing pipeline itself writes into the masked band and into out-of-bounds padding; the two mean-fill rows test the sensitivity of the tibiofemoral conditions to that choice. The baseline is recomputed locally in the same process and on the same device as every perturbed condition, so no difference in this panel compares a locally recomputed risk against a published one. Intervals are from a paired patient-level bootstrap of 2,000 replicates. Every anatomic occlusion interval crosses zero except the mean-fill joint-only condition, so these rows establish the direction and size of a shift in predicted risk and do not rank the compartments against one another; the anatomic conditions were not adjusted for multiplicity. The last row is a pipeline check and not a leakage test. Re-zeroing the already-blank band leaves 677 of 734 patients bit-identical, which proves the perturbation path is exact. The pre-specified border-band-only control is not reported here and has been withdrawn from the paper: preprocessing already zeroes that band, so supplying the band alone hands the network a near-constant input, and the discrimination that results measures the destruction of the input rather than any property of the model or any burned-in text. The evidence about text is the residual-marker row and the two widened bands, all of which perturb pixels that carry signal and all of which are upper bounds. These analyses are post-hoc on an already-read split.

Table S4. What each radiographic view contributes.

**Panel A. Attention weight assigned to each view by the frozen aggregators**						
**Comparison**	**Patients**	**Model**	**Frontal**	**Lateral**	**Sunrise**	**Lateral outweighs frontal**
One frontal and one lateral crop	218	M3 all views	0.344 (0.103)	0.656 (0.103)	not applicable	95.4%
One frontal and one lateral crop	218	M4 combined	0.252 (0.120)	0.748 (0.120)	not applicable	96.8%
All three views present	32	M3 all views	0.213 (0.069)	0.458 (0.161)	0.329 (0.148)	90.6%
All three views present	32	M4 combined	0.150 (0.064)	0.492 (0.175)	0.358 (0.167)	93.8%
**Panel B. Discrimination after withholding views at input from the frozen networks**						

Model	Views supplied	IPCW AUROC at 1825 days	Change versus all views (95% CI)	P
M3 all views	frontal, lateral and sunrise	0.824	reference	reference
M3 all views	frontal only	0.746	−0.078 (−0.142 to −0.012)	0.024
M3 all views	frontal and lateral, sunrise withheld	0.827	+0.004 (−0.003 to 0.013)	0.42
M3 all views	lateral and sunrise, frontal withheld	0.843	+0.019 (−0.026 to 0.066)	0.43
M4 combined	frontal, lateral and sunrise	0.807	reference	reference
M4 combined	frontal only	0.744	−0.063 (−0.131 to 0.001)	0.056
M4 combined	frontal and lateral, sunrise withheld	0.807	−0.001 (−0.014 to 0.014)	0.88
M4 combined	lateral and sunrise, frontal withheld	0.814	+0.007 (−0.032 to 0.049)	0.78

Attention weights sum to one over the views a patient actually contributed, so a patient holding only a frontal radiograph contributes a weight of one to it trivially and the marginal share over all patients is inflated by those cases. Panel A therefore reports weights within patients holding one crop of each view. Values are means with standard deviations, and the final column is the percentage of those patients in whom the lateral weight exceeds the frontal weight; in the three-view rows it is the percentage in whom the lateral exceeds the frontal.

Panel B takes one frozen multi-view network, withholds views at input and re-scores. Because one condition requires a frontal crop and another requires a non-frontal crop, every row is restricted to the intersection of all four conditions' rosters, which is the 315 test patients who contributed both a frontal crop and at least one non-frontal crop. That is not the same as the number of patients holding more than one view, which is 316, nor the number contributing at least one non-frontal crop, which is 321, nor the pre-preprocessing count of patients whose selected study was not frontal-only, which is 322 on the 740 patients with an image and 323 on the full 741-patient test split. Every difference in Panel B is computed before rounding, so the M4 all-views reference and the M4 condition with the sunrise view withheld both print as 0.807 while their difference is −0.001. Intervals are from a paired patient-level bootstrap of 2,000 replicates. Panel B answers what a view contains, which is a different question from what a model trained without that view achieves across the whole split; the between-model comparison is in Table 2, and only 43 percent of this population has a second view at all. Only 37 of the 740 test patients with an image have a sunrise film, so the tight interval around zero for the sunrise rows is a statement about that 5 percent. All of this table is post-hoc on an already-read split.

Table S5. Prediction within Kellgren-Lawrence strata.

Panel A. Observed 5-year incidence by tertile of frontal-model predicted risk, formed within each stratum

Stratum	Patients / events	Lowest tertile	Middle tertile	Highest tertile	Log-rank P
All grades	707 / 98	4.3% (2.0 to 9.0)	15.4% (10.1 to 23.3)	44.9% (36.2 to 54.7)	6.9×10^{-23}
Grades 0 to 1	144 / 3	4.0% (0.6 to 25.2)	2.4% (0.3 to 16.1)	5.9% (0.9 to 35.0)	.92
Grade 2	266 / 30	4.5% (1.4 to 13.7)	17.0% (8.7 to 31.7)	29.3% (18.9 to 43.7)	8.6×10^{-5}
Grades 3 to 4	297 / 65	11.3% (5.7 to 21.5)	31.5% (21.0 to 45.4)	52.7% (39.2 to 67.5)	2.1×10^{-8}

Panel B. Discrimination within stratum, under the pre-specified 50-event suppression rule

Stratum	Patients / events	M2 frontal, IPCW AUROC (95% CI)	M4 combined, IPCW AUROC (95% CI)	M4 minus M2 (95% CI)	P
Grades 0 to 1	144 / 3	suppressed, fewer than 50 events	suppressed, fewer than 50 events	not estimated	not estimated
Grade 2	266 / 30	suppressed, fewer than 50 events	suppressed, fewer than 50 events	not estimated	not estimated

https://doi.org/10.3390/jimaging12100474

Grades 3 to 4	297 / 65	0.802 (0.716 to 0.880)	0.841 (0.764 to 0.908)	0.039 (−0.025 to 0.105)	0.23

Panel C. Incremental value of the model risk score over the grade, Cox proportional-hazards models on 707 patients and 98 events

Model risk score	Specification	Hazard ratio per SD (95% CI)	Likelihood-ratio chi-square, 1 df	P
M2 frontal	Grade as a numeric covariate (primary)	3.01 (2.26 to 4.00)	65.7	5.1×10^{-16}
M2 frontal	Grade as three categories	3.03 (2.29 to 4.01)	69.2	8.8×10^{-17}
M2 frontal	Baseline hazard stratified by grade	3.01 (2.27 to 3.97)	68.3	1.4×10^{-16}
M2 frontal	No grade in the model	3.09 (2.44 to 3.91)	102.0	5.4×10^{-24}
M4 combined	Grade as a numeric covariate (primary)	2.76 (2.16 to 3.53)	69.6	7.1×10^{-17}
M4 combined	Grade as three categories	2.81 (2.20 to 3.59)	72.6	1.6×10^{-17}
M4 combined	Baseline hazard stratified by grade	2.81 (2.20 to 3.60)	72.2	2.0×10^{-17}
M4 combined	No grade in the model	2.98 (2.40 to 3.70)	104.4	1.6×10^{-24}

The inferred contralateral Kellgren-Lawrence grade was recorded for 707 of the 741 test patients, who carry 98 events. Strata are defined by bin edges declared once at 1.5 and 2.5 on the numeric grade, left-closed, so an averaged half-grade joins the higher stratum; the whole stratification was repeated with the opposite tie rule and with a single cut at 2.5, and neither changed any conclusion. Panel A forms tertiles of predicted risk inside each stratum, reports the Kaplan-Meier cumulative incidence of contralateral arthroplasty at 5 years with Greenwood 95% intervals, and gives an unadjusted log-rank test across the three tertiles; the "All grades" row is the pooled comparison over the same 707 patients and double-counts the strata beside it, so it is shown for context and never summed with them. The grades 0 to 1 stratum carries 3 events in 144 patients, so its intervals span almost the whole range and no separation is observable there; that is an uninformative cell rather than a demonstrated null.

Panel B honours the pre-specified 50-event floor without modification. Only one stratum can clear it, and that is arithmetic rather than a shortfall of effort: two strata each carrying 50 events require at least 100, and this population has 98, so no partition of it leaves two estimable strata. The one estimable M2 interval is 0.163 wide and is flagged as imprecise by the pre-declared width criterion. The contrast in the last two columns is paired on the same 297 patients; 122 of them (41.1%) contributed a frontal film only, so it is diluted, though less so than the whole-cohort contrast in Table 2.

Panel C enters the model risk score as the complementary log-log of the arm's predicted 5-year risk, standardized to unit standard deviation. That scale is a linear predictor and the frozen recalibration is affine on it, so every estimate in this panel is identical whether or not the recalibration was applied. Each likelihood-ratio test compares the model with the score against the identical model without it, on the same patients. With the grade and the M2 score both in the model, the grade's own hazard ratio is 1.04 per unit (0.81 to 1.34; P = 0.75), and with the M4 score it is 1.17 (0.92 to 1.48; P = 0.21). Schoenfeld residual tests on a rank time transform gave P values from 0.41 to 0.98 for every term of every specification. The categorical specification is reported as a sensitivity on the score term and not as an estimate of the grade's own effect, because its reference stratum carries 3 events and its grade contrasts are correspondingly unstable. Every analysis in this table is post-hoc on an already-read split, and the grade it is measured against is itself model inferred, so a claim of information beyond the grade means beyond an automated grade rather than beyond a radiologist's reading.

Table S6. Protocol deviation cross-walk.

Reviewer 2 asked for the deviation numbering to leave the main text, and it has: no deviation identifier now appears in the Abstract, Introduction, Methods, Results, Discussion, Conclusion or the main tables. This table is what replaces it. Every main-text claim that carried a deviation marker is quoted below beside the register entry that qualifies it and what that entry records. Sixteen register entries qualify a main-text claim and all sixteen appear here. The full register accompanies this report as supplementary material.

Deviation	Main-text claim it qualifies	Where the claim appears	What the deviation records	Register entry
D2	"recovered by a deterministic pre-index rule for 1,828, 49.3% of the cohort"; "that rule was validated against manual adjudication in 200 development patients, where it agreed in 196 (98.0%)"	Methods, Image-quality and laterality verification; Table 1 note	The primary cohort is the side-recovery arm rather than the strict coded-laterality cohort protocol section 6 requires, so the index side is inferred for 49.3% of patients	D2, 2026-07-21, author-approved
D17	"no acquisition tag was released and the study date carries an unconfirmed per-patient shift"	Results, Robustness strata; Supplementary Table S2 note	Protocol section 25 robustness items were not run, including performance by calendar era, because the written confirmation section 17 requires that de-identified dates are comparable across patients was never obtained	D17, 2026-07-24, deferred, corrected 2026-08-10
D20	"Two predictors depart from their pre-specified definitions, and the ridge penalty sits at the unwidened edge of its grid"	Methods, Clinical comparators, missing data and statistical analysis	The comorbidity predictor uses all pre-index history where protocol Table 6 specifies the prior 5 years. A longer lookback can only raise prevalence	D20, 2026-07-24, flagged for author decision
D21	"Two predictors depart from their pre-specified definitions, and the ridge penalty sits at the unwidened edge of its grid"	Methods, Clinical comparators, missing data and statistical analysis	The pain predictor is the highest knee pain score in the prior 365 days where protocol Table 6 specifies the most recent contralateral or bilateral score in the prior 180 days. A maximum rises with the number of pain encounters, so it carries utilization as well as severity	D21, 2026-07-24, flagged for author decision
D22	"the section 23(ii) outcome review was completed at revision, after the split had been read"; "the pre-specified 200 records stood and a 60-record test-split extension took it to 260"	Methods, Image-quality and laterality verification; Results, Manual review and burned-in markers; Table 1 note	Protocol section 23 manual quality assurance depends on two independent human reviewers. The section 7 laterality audit and the section 23(i) image review are complete and reported; the section 23(ii) outcome-record review is also complete and reported, with a design-weighted label error rate of 0.00% that did not exceed the pre-declared 2% threshold. None of the three was complete when the locked split was read	D22, opened 2026-07-24, closed 2026-09-15

D23	"Two predictors depart from their pre-specified definitions, and the ridge penalty sits at the unwidened edge of its grid"	Methods, Clinical comparators, missing data and statistical analysis	The selected ridge penalizer sits at an edge of the pre-specified grid and the grid was not widened, because widening it after seeing the selection criterion is the post hoc selection protocol section 27 warns against	D23, 2026-07-24, resolved by D24
D24	"Two predictors depart from their pre-specified definitions, and the ridge penalty sits at the unwidened edge of its grid"	Methods, Clinical comparators, missing data and statistical analysis	The penalizer selection metric moved from the validation C-index on 54 events to the cross-validated C-index inside the training split on 373 events. The pre-specified grid itself was not widened	D24, 2026-07-26, author decision implemented
D28	"DenseNet121 became the primary encoder and ConvNeXt-Tiny the single robustness architecture"; "We replaced the pre-specified ConvNeXt-Tiny encoder with DenseNet121 after a single configuration failed to fit, and registered that as a trade"	Methods, Network architecture and multi-view aggregation; Discussion; Table 3 note	The backbone changed from the pre-specified ConvNeXt-Tiny to DenseNet121 and the optimization was retuned, after the pre-specified configuration did not fit the training data. The no-architecture-tournament guarantee is forfeited, so every comparison across encoders is exploratory. The entry carries later corrections to figures it first published, and none of those figures is quoted in this paper, and a further dated correction in the light of the D41 sweep	D28, 2026-07-28, author decision, corrected, most recently 2026-09-10
D29	"its estimator, thresholds and arms were fixed after the single read"; "These too are exploratory"	Methods, Clinical comparators, missing data and statistical analysis; Results, Calibration and clinical usefulness	Decision-curve analysis ran at manuscript stage. The estimator, the threshold grid and the arm set were specified after the single permitted read, so every threshold-specific finding is exploratory	D29, 2026-07-30, analyst decision documented
D35	"after that single read, so all are exploratory"; "both single-view contrasts are post hoc"; "Every imaging, leakage, view-ablation, within-grade and robustness result is post-hoc on an already-read test split"	Methods, umbrella statement; Results, The radiograph against the clinical record; Discussion; Supplementary Tables S2, S3, S4 and S5 notes	Every imaging analysis added at revision is computed on the same test split after it was read, by people who had seen its results. The single-read guarantee covers the pre-specified primary estimates and nothing else	D35, 2026-08-10, scope declaration
D36	"The section 22 interpretability package was not run at first submission"; "the label-randomization control requires retraining and was not performed"	Methods, Image interpretability and leakage controls; Supplementary Tables S3 and S4 notes	Protocol section 22 explainability was never run and no register entry covered the gap. The package was executed and reported at revision stage, minus the label-randomization control, which requires retraining on permuted labels and was cut. The entry stays open on that one item	D36, 2026-08-10, executed at revision, open on one item

https://doi.org/10.3390/jimaging12100474

D37	"The sealed-read artifact records no interpreter, device or precision"	Methods, Training and internal validation	The artifact recording the single permitted read carries no interpreter, device, platform or mixed-precision field, so the environment that produced the headline out-of-sample estimates survives only in the training-arm file	D37, 2026-08-10, reproducibility gap
D38	"the published environment file describes the revision workstation"	Methods, Training and internal validation	The environment file published beside the code describes the local workstation used for the revision analyses rather than the cloud graphics processor that trained and scored the reported models	D38, 2026-08-10, reproducibility gap
D39	"running after border zeroing, so the band can end up non-zero"; "a masked band that is not exactly zero on 145 crops (11.9%)"	Methods, Image preprocessing; Results, Manual review and burned-in markers; Supplementary Table S3 Panel B note	Marker masking runs after border masking and refills each accepted blob with the surrounding ring median, so the masked band is not exactly zero on 11.9% of test crops. The measured cost is a change in discrimination of -0.0002 (95% CI -0.0009 to 0.0000)	D39, 2026-08-11, permanent and analytically priced
D40	"The 200-patient section 7 laterality audit and the 400-image two-reviewer review (section 23(i)) are complete"; "Laterality, assessable on the 318 panels carrying a full film, was correct on at least 99%"	Methods, Image-quality and laterality verification; Results, Manual review and burned-in markers	Protocol section 23(i), the 400-image two-reviewer image review, was performed and is reported. The entry's earlier record of a decline is withdrawn in place rather than deleted	D40, 2026-08-12, decline withdrawn 2026-08-13
D41	"before the sweep ran we committed in writing that a sweep which made ConvNeXt-Tiny converge would make the abandonment premature, and that this Discussion would have to say so"; "the robustness arm the protocol asked for was never a fair test of the architecture it names"	Discussion	A staged ConvNeXt-Tiny hyperparameter sweep was specified at revision stage, reported on training negative log-likelihood so that no split is read, and registered before it was run together with the consequence of an unfavourable result: if any configuration made the abandoned encoder converge, the register and the manuscript would both have to record that the abandonment was premature. The sweep ran, a configuration converged, and the entry discharges that commitment on its own terms	D41, 2026-09-04, pre-specified at revision stage, executed 2026-09-08/09, pre-committed consequence discharged 2026-09-10

Every quotation above is taken verbatim from the current main text. Some are clauses rather than whole sentences, and each is the clause the deviation marker sat on; where a sentence was reworded during revision the quotation gives the current wording rather than the wording of the submitted version, and no claim listed here was dropped from the paper. Where one deviation qualifies more than one claim, every location is listed; where several

deviations qualify the same claim, that claim is repeated against each of them. Deviations not cited in the main text are documented in the register alone.

Table S7. Comparison with the prior work the Introduction cites.

Reviewer 1 asked what this study contributes beyond prior deep-learning work on knee osteoarthritis and arthroplasty prediction. This study claims no algorithmic novelty. Panel A sets it beside the studies the Introduction cites, characterized only as the Introduction characterizes them. Panel B states what this study does claim, and the result each claim rests on.

Panel A. Outcome and setting, as this manuscript characterizes each study

Study	Outcome predicted	Characterization given in the Introduction of this manuscript
Kim et al. [9]	Radiographic knee osteoarthritis development	A machine-learning study of knee osteoarthritis prognosis predicting a radiographic endpoint, with the contralateral knee as the target
Lee et al. [10], Tiulpin et al. [11]	Radiographic knee osteoarthritis progression	Machine-learning studies of knee osteoarthritis prognosis predicting a radiographic endpoint, progression rather than arthroplasty
Leung et al. [12], Rajamohan et al. [13]	Total knee arthroplasty	Deep-learning models that predict arthroplasty from radiographs and magnetic resonance imaging with high discrimination, but for the imaged knee, in research cohorts with standardized acquisition
Liu et al. [14], systematic review and meta-analysis	The outcomes of the studies it pools, which it reports as heterogeneous	A meta-analysis pooling C-indices of 0.71 to 0.81 while noting heterogeneous outcomes and almost no external validation
This study	Contralateral arthroplasty within 5 years of a unilateral index arthroplasty	A discrete-time survival model predicting contralateral arthroplasty from the routine preoperative radiograph in a health-system population, with a test split locked before fitting and read once

Panel B. What this study claims, and the result each claim rests on

Claim	The result it rests on
The clinical question	Whether the film already taken of the other knee predicts a second arthroplasty, asked in a health-system population rather than a research cohort
The cohort and the imaging input	A single health-system radiograph archive, routine preoperative frontal, lateral and sunrise films of the contralateral knee, no standardized acquisition protocol, 3,709 patients, of whom 741 form a test split locked before fitting and read once (Table 1)
A contrast inside a fixed radiographic grade	Adding the standardized image risk score to the inferred Kellgren-Lawrence grade in a Cox model gives a hazard ratio of 3.01 per standard deviation (95% CI 2.26 to 4.00) and a likelihood-ratio chi-square of 65.7 on 1 degree of freedom ($P < 0.001$), while the grade's own effect falls to 1.04 (0.81 to 1.34; $P = 0.75$). Observed 5-year incidence rises across risk tertiles inside grade 2 from 4.5% to 29.3% (Supplementary Table S5)
Quantitative attribution, occlusion and leakage controls, reported together	Grad-CAM and integrated gradients reported as attribution mass by predefined region with enrichment against area share; occlusion of the medial compartment, the lateral compartment, the patellofemoral zone and the whole joint region, with a joint-region-alone negative control and both tibiofemoral conditions repeated under a mean fill; cascading parameter randomization; residual-marker blanking; and the masked band widened to 62 and to 93 pixels (Supplementary Table S3)

https://doi.org/10.3390/jimaging12100474

The view-necessity decomposition, separated into two estimands	A between-model frontal-only contrast on the whole test split answers what a deployed single-view model loses on this population (Table 2); withholding views at input from the frozen network, on the 315 test patients contributing a frontal crop and at least one non-frontal crop, answers what a second film contains (Supplementary Table S4)

Panel A characterizes each prior study only as the Introduction of this manuscript already characterizes it, and asserts nothing beyond that. Whether any individual study reported a contrast inside a fixed radiographic grade, quantitative attribution or leakage controls, or a view-necessity analysis cannot be settled from a title or an abstract; those full texts were not read for this table, and no such characterization of another group's work is made here.

The contrast is therefore drawn at the level of the literature, where the Introduction already draws it. Discrimination is reported without reporting what the network reads: attribution maps are common, quantitative attribution and occlusion experiments are rare, and leakage controls on burned-in text and borders rarer still. The imaging requirement is seldom examined, so how many radiographs a deployed model needs is open. No study asks whether the film already taken of the other knee predicts a second arthroplasty in a health-system population, or isolates what the image adds inside a fixed grade. Panel B is what this study puts against that background. The analyses in the last three rows of Panel B are post hoc on an already-read test split, as the notes to Supplementary Tables S3, S4 and S5 record.

Table S8. ConvNeXt-Tiny hyperparameter sweep on the training split.

**Panel A. Screening stage, one seed and 10 epochs per configuration**				
**Configuration**	**Encoder learning rate**	**Layer-wise learning-rate decay**	**Minimum training negative log-likelihood over 10 epochs**	**Epoch at which the minimum occurred**
Config 1	3×10^{-5}	none	0.489268	9
Config 2	3×10^{-5}	0.75	0.508879	9
Config 3	3×10^{-5}	0.90	0.476405	9
Config 4	1×10^{-4}	none	0.609320	2
Config 5	1×10^{-4}	0.75	0.495818	9
Config 6	1×10^{-4}	0.90	0.609320	2
Config 7	3×10^{-4}	none	0.610754	4
Config 8	3×10^{-4}	0.75	0.611139	9
Config 9	3×10^{-4}	0.90	0.611291	9
Config 10	1×10^{-3}	none	0.610400	9
Config 11	1×10^{-3}	0.75	0.612160	9
Config 12	1×10^{-3}	0.90	0.613084	9
**Panel B. Confirmation stage, one factor changed at a time on the best screened configuration, five seeds per cell, and the pre-sweep reference arms**				
**Configuration**	**Warmup epochs**	**Drop path**	**Input resolution, px**	**Training negative log-likelihood, 5-seed mean of per-seed minimum**
Best screened configuration, reference cell	0	0.0	512	0.316717
Reference cell, warmup raised	5	0.0	512	0.273942

https://doi.org/10.3390/jimaging12100474

Reference cell, drop path raised	0	0.1	512	0.443548
Reference cell, input resolution lowered	0	0.0	224	0.325428
Pre-sweep ConvNeXt-Tiny baseline, R1 arm	2	0.0	512	0.607128
Pre-sweep DenseNet121 baseline, M2 arm	2	not applicable	512	0.371118

This sweep was specified in response to peer review and has been run. The sweep is reported on training negative log-likelihood, which is the quantity that was used to abandon the originally pre-specified encoder, so no held-out split is read to produce this table. Panel A screens 12 configurations on one seed for 10 epochs, crossing four encoder learning rates with three layer-wise learning-rate decay settings; drop path is held at 0.0 throughout the screen, and every other training setting is the frozen training contract used by the published arms. Panel B carries the single best-screening configuration forward to five seeds and moves one factor at a time from its reference cell: warmup, then drop path, then input resolution, each against the same reference and with the other two held at their reference levels. That is four cells, not the sixteen a full crossing of the best two configurations would give; deviation D41 registered the crossing and deviation D48 records the narrowing. Each difference is therefore the effect of one factor at the reference levels of the other two, and no interaction is estimated. The 224-pixel cell tests the hypothesis that the original abandonment asserted but never evaluated. The configuration carried forward is recorded as s1_lr3e-05_llrd0.9_wu2_dp0_px512_fr3; the second-best screened cell was dropped at that point and appears in no Panel B row. That configuration name carries the warmup of 2 that the screen ran at, which is the production value in the frozen training contract, and no Panel B row shows it: warmup is itself one of the three swept factors, so it was reset from 2 to the reference level of 0 before the one-factor moves began, and Panel B's warmup axis is {0, 5}. Only the encoder learning rate and the layer-wise decay are carried across from the screen unchanged. The two reference rows are the runs already recorded in the training history and are reported on the same quantity, the mean over five seeds of each seed's minimum training negative log-likelihood, so a swept configuration improves on the abandoned run when its value falls below 0.607128 and matches the primary encoder when it falls to 0.371118. Every swept cell ran a fixed epoch budget with no validation pass and therefore no early stopping, 10 epochs per cell in Panel A and 17 in Panel B. The Panel B budget equals the longest the M2 reference run itself ran before early stopping, and is well short of the sweep module's 40-epoch stage-2 default, so no Panel B cell is helped by a longer descent than the rows it is read against. Panel A ran shorter than both reference rows, at 10 epochs against 17 and 18, so the screen is if anything conservative (deviation D46). Neither published arm was ever given a drop-path rate: the frozen training contract has no drop-path key, and the trainer passes no such argument when none is set, so each reference row ran at whatever its own encoder does with stochastic depth left alone. The two cells differ because the architectures do. ConvNeXt-Tiny has a stochastic-depth parameter that defaults to 0.0; DenseNet121 has none at all, so its cell reads "not applicable" rather than a zero that would assert a setting the architecture does not have. The specification was written after the locked split had been read, so what the sweep returns is exploratory.

Table S9. A second encoder that converges: ResNet-50 against the published frontal-view arms.

Arm	Encoder	Training negative log-likelihood, 5-seed mean of per-seed minimum	Validation negative log-likelihood, 5-seed mean of per-seed minimum	Validation patients / events	Validation IPCW AUROC at 1825 days (95% CI)	Validation Harrell C (95% CI)
R2 radiograph, frontal view, new architecture arm	ResNet-50	0.499498	0.591330	370 / 54	0.803 (0.726 to 0.876)	0.766 (0.698 to 0.831)
M2 radiograph, frontal view, primary arm	DenseNet121	0.371118	0.581264	370 / 54	0.828 (0.751 to 0.898)	0.777 (0.715 to 0.836)
R1 radiograph, frontal view, robustness arm	ConvNeXt-Tiny	0.607128	0.617506	370 / 54	0.700 (0.607 to 0.791)	0.698 (0.627 to 0.764)

The ResNet-50 arm was specified in response to peer review and has been trained. It trained on the same 70% training split, with the same five pre-specified seeds, the same augmentation cap and the same frozen training contract as every published arm, and its encoder was already in the trainer's list of known architectures, so the arm needed a configuration entry and no change to the model code. Training and validation negative log-likelihood are each the mean over five seeds of that seed's own minimum, which is the quantity the two reference rows carry. That mean is not uniform across the five seeds, and the spread travels with it (deviation D42): the per-seed training minima run from 0.451590 to 0.610035, and the top of that range is one degenerate seed, 20250721, whose retained checkpoint is its own epoch 0 and which early-stopped after 9 epochs. The other four are tightly grouped, none of them above 0.485692. That seed also gives this arm the widest seed-to-seed validation spread in the ladder, a standard deviation of 0.024247 over the five per-seed minimum validation negative log-likelihoods. Discrimination is reported on the 10% validation split, which holds 370 patients with a frontal crop and 54 events; that denominator is a property of the frozen split and the prepared crops, so it is the same for all three rows. The locked test split was not read for this table, so no value here is comparable to a test-split estimate in Table 3, and no patient-level prediction file exists for the ResNet-50 arm, so no paired comparison against the DenseNet121 row can be constructed from these numbers. Intervals are 95% percentile intervals from a patient-level bootstrap of 2,000 replicates. The specification was written after the locked split had been read, so what the arm returns is exploratory.

The two reference rows are re-scored from the frozen per-patient prediction files rather than transcribed from the published validation table, because that table is stale for the DenseNet121 arm; the ConvNeXt-Tiny row reproduces identically from either source, which is what identifies the one that moved. Deviation D49 records the discrepancy and that the frozen artifacts are authoritative.

The two validation discrimination figures in the reference rows carry a caveat, and it applies to the ResNet-50 row on the same terms. The analysis pipeline's own validation comparison table suppresses every validation contrast involving an image arm, because each arm's checkpoint is chosen by early stopping on the same validation split it is then scored on, so its validation metrics are optimistic; the DenseNet121 arm is flagged there for a validation negative log-likelihood ending 0.157 above its own five-seed minimum against a ceiling of 0.1. That figure is recomputed from the frozen training history, for the same reason the reference rows above are re-scored rather than transcribed; the published convergence table

prints 0.172, which is a render of the superseded artifact state and reproduces only from the archived training history (deviation D49). The flag is unchanged either way, because both values exceed the 0.1 ceiling. These two figures are therefore printed as a like-for-like reference for the new arm and not as performance estimates, they are not comparable to anything in Table 3, and the ResNet-50 arm was checkpointed the same way and carries the same optimism. Only the test-split estimates in Table 3 are free of it.

Table S10. Fusion ablation: attention pooling against mean, max and feature concatenation

Model	Aggregator	IPCW AUROC at 1825 days	Change versus attention pooling (95% CI)	P
**Panel A. Aggregator replaced at inference on the frozen networks, whole test roster**				
M3 all views	Masked attention pooling, as trained	0.840 (0.787 to 0.889)	reference	reference
M3 all views	Masked mean pooling	0.853	+0.013 (0.002 to 0.026)	0.018
M3 all views	Masked max pooling	0.853	+0.013 (0.002 to 0.025)	0.015
M4 combined	Masked attention pooling, as trained	0.844 (0.791 to 0.894)	reference	reference
M4 combined	Masked mean pooling	0.854	+0.009 (−0.006 to 0.025)	0.23
M4 combined	Masked max pooling	0.854	+0.009 (−0.005 to 0.024)	0.22
**Panel B. The same replacement restricted to the patients who contributed two or more crops**				
Model	Aggregator	IPCW AUROC at 1825 days	Change versus attention pooling (95% CI)	P
M3 all views	Masked attention pooling, as trained	0.860	reference	reference
M3 all views	Masked mean pooling	0.874	+0.014 (−0.007 to 0.037)	0.21
M3 all views	Masked max pooling	0.873	+0.014 (−0.007 to 0.036)	0.20
M4 combined	Masked attention pooling, as trained	0.849	reference	reference
M4 combined	Masked mean pooling	0.853	+0.004 (−0.020 to 0.030)	0.78
M4 combined	Masked max pooling	0.853	+0.004 (−0.019 to 0.028)	0.80
**Panel C. Feature concatenation, which changes the head input dimension and must be retrained, validation split only**				
Model	Aggregator	Validation IPCW AUROC at 1825 days (95% CI)	Validation negative log-likelihood, 5-seed mean of per-seed minimum	
M3 all views	Masked attention pooling, as trained	0.833 (0.756 to 0.905)	0.575165	
M3 all views	Feature concatenation over views, retrained	0.841 (0.765 to 0.913)	0.566187	
M4 combined	Masked attention pooling, as trained	0.835 (0.756 to 0.908)	0.564381	
M4 combined	Feature concatenation over views, retrained	0.846 (0.771 to 0.914)	0.578321	

Every panel of this table was specified in response to peer review, and every panel has been run. Panels A and B come from one re-scoring of the frozen ensembles, reported on two denominators. Panel C comes from a retrain scored on the validation split. In each panel the

attention-pooled rows are the frozen arms' own estimates and are the comparator for the rows below them.

The AUROC column of Panels A and B carries no interval because the ablation bootstraps the paired difference between two aggregators and not each aggregator's marginal AUROC, so an interval exists for the change column and not for the level. The two attention reference cells in Panel A are the exception: they are published Table 3 estimates and carry their own published interval.

Panels A and B replace the view aggregator at inference on the frozen five-seed ensembles; the state dictionary is unchanged and the attention pooling parameters go unused. The encoders and the risk head were trained under masked attention pooling, so these rows measure how much the learned representation depends on attention weighting and not what a mean-pooled or max-pooled model would have achieved had it been trained that way.

The denominators are the thing a reader is most likely to get wrong, so they are stated exactly. Pooling runs over crops, not views. Every pooling function is the identity for a patient who contributed exactly one crop, and 367 of the 740 test patients with a prepared crop contributed exactly one, 49.6% of them, carrying 35 of the 106 events. Their predictions do not change under any aggregator, but they stay in Panel A, where a swap can reorder them against multi-crop patients whose risks it moves. Panel A rests on all 740 patients carrying 106 events, which is the roster both arms score in Table 3. Panel B repeats the comparison on the 373 patients who contributed two or more crops, carrying 71 events, which is the population in which the aggregator can differ at all. That is not the 315-patient roster of Supplementary Table S4 Panel B: view withholding needs a frontal crop and a non-frontal crop, whereas pooling is already non-trivial for a patient holding two frontal crops and no lateral film, so the 315 are a subset of these 373 and the two analyses answer different questions. The ablation code counts this denominator from each arm's own scored crops and writes it on every output row, so the published figure is measured at run time rather than transcribed from a cohort table.

Feature concatenation changes the input dimension of the risk head, so it cannot be swapped at inference and the two concatenation arms in Panel C were retrained from scratch. Both arms are reported on the 371 validation patients carrying 54 events, the roster the all-views arms score on that split, and the locked test split was not read for them, so no value in Panel C is comparable to one in Panels A and B. The two attention-pooled reference values in Panel C are validation estimates of arms whose checkpoints were chosen by early stopping on that same validation split, which the analysis pipeline's own validation comparison table suppresses for exactly that reason; they are optimistic, are printed only so the retrained concatenation arms have a like-for-like comparator, and are not performance estimates.

Panel C has no change column. A paired contrast against attention pooling was specified with the rest of this table but cannot be estimated, for two independent reasons. Each concatenation arm carries the same arm name as its attention-pooled comparator, and the evaluation keys each scored arm by that name, so one run cannot hold both. The analysis pipeline also suppresses every validation contrast that names an image arm flagged for overfitting, as the note to Supplementary Table S9 describes. All four arms in this panel are flagged, so the contrast would be suppressed whatever the concatenation arms scored. The concatenation values carry the same optimism as the attention-pooled references, because their checkpoints were also chosen by early stopping on the validation split. None of them is comparable to a value in Table 3.

https://doi.org/10.3390/jimaging12100474

Intervals are 95% percentile intervals from a paired patient-level bootstrap of 2,000 replicates and P values come from the same resample. Panels A and B re-score predictions on an already-read split, so the whole table is reported as exploratory.

Supplementary Figures

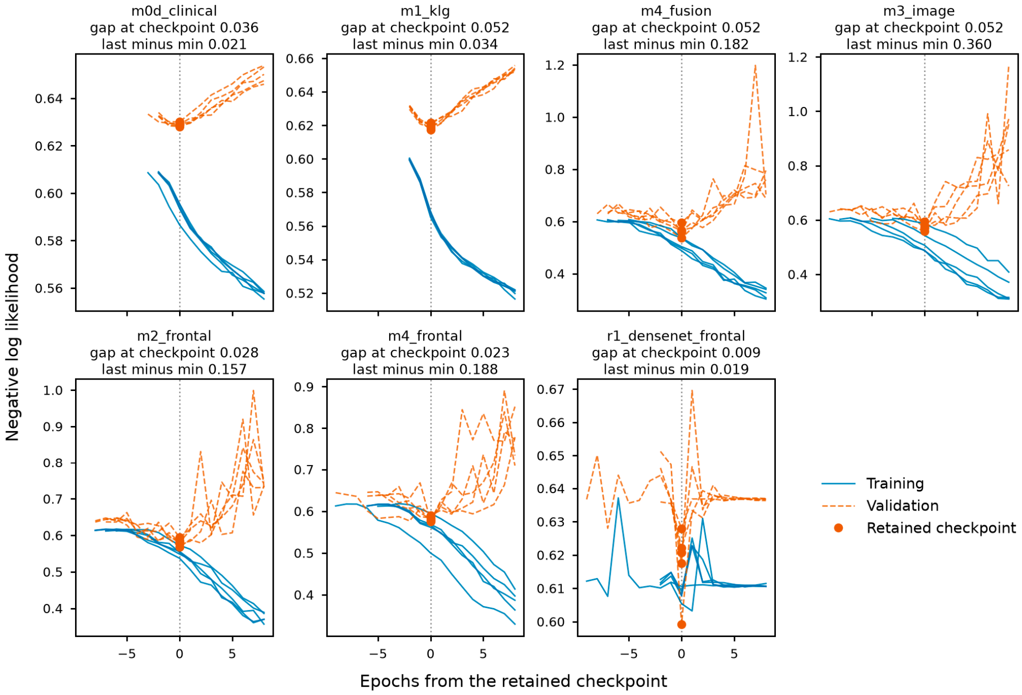

Figure S1. Training and validation loss for every arm and every seed. Learning curves for all seven arms and all five pre-specified seeds. The horizontal axis is epochs from the RETAINED checkpoint, so zero is the epoch whose weights were kept, and the filled marker on each pair of curves is that epoch. Training ran a further 8 epochs past it in every one of the 35 series, because that is the early-stopping patience. The number under each panel is the validation-minus-training negative log likelihood at the retained checkpoint, beside the quantity reported as the overfitting gap in the convergence table, which is a different measurement: that one is the validation loss at the LAST epoch minus its own minimum, so it measures how far training ran after the model that was kept and not how far the kept model was from its training data. The training loss is a running average taken in training mode with dropout and augmentation active while the weights update, and the validation loss is a clean evaluation pass, so every gap shown here is a LOWER BOUND on the train-validation gap rather than a measurement of it.

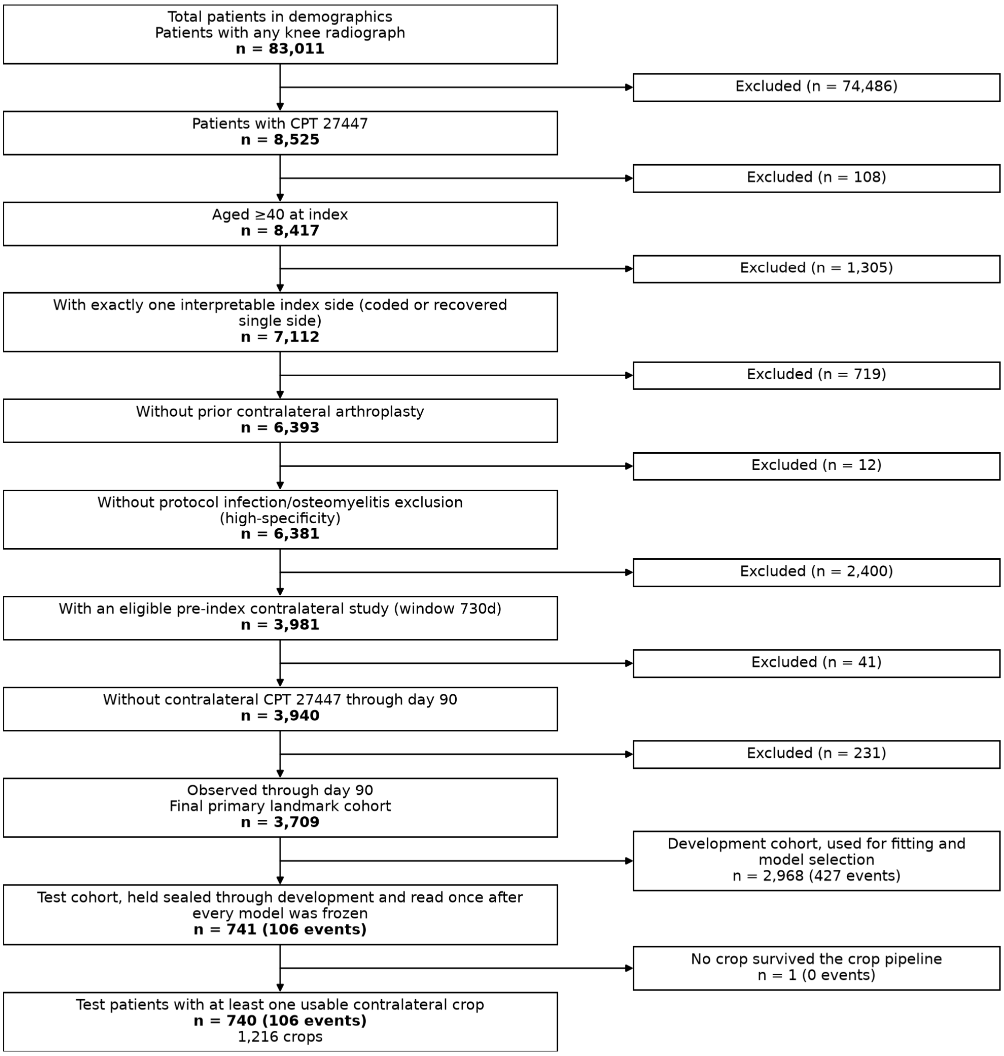

Figure S2. Cohort assembly from the source registry to the test patients with a usable contralateral radiograph. Patient flow for the primary landmark cohort. Boxes in the left column give the number of patients retained after each eligibility criterion, and the branches to the right give the number removed at that step. Counts down to the 3,709-patient landmark cohort are taken from the cohort assembly ledger; the final two steps are recomputed at figure time from the locked patient-level split assignment and the record of which radiographs were successfully prepared for the model. The 741 patients assigned to the test split, carrying 106 events, were set aside unread and contributed to no model fitting and no hyperparameter choice; they were read once, after every model was frozen, and the evaluation reported in this manuscript is that single read. One test patient had no usable image of the contralateral knee; that patient was event free, so all 106 test events were retained.

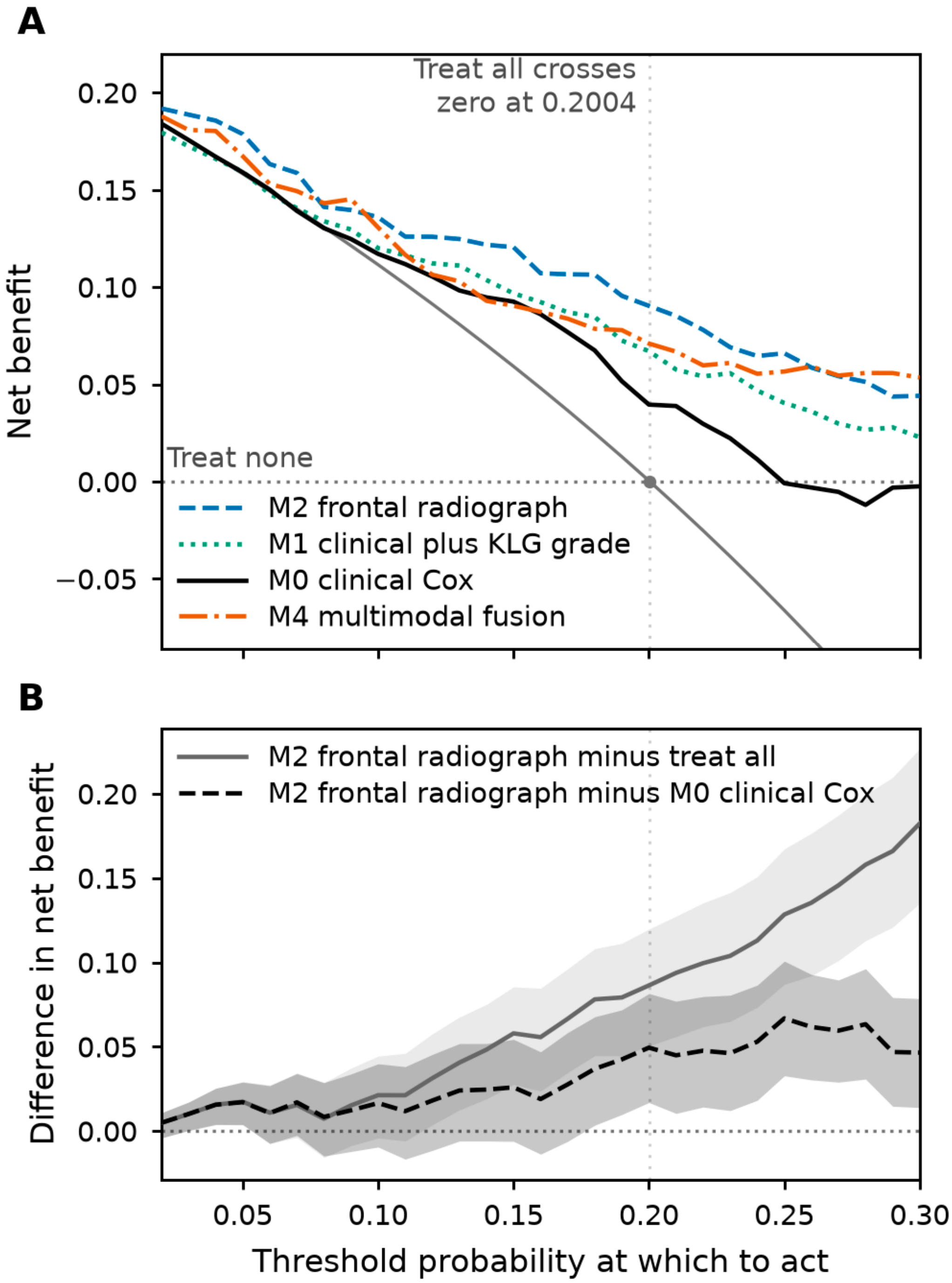

Figure S3. Decision-curve analysis of net benefit against threshold probability in the test split. Decision-curve analysis in the test split. Panel A gives net benefit, in net true positives per patient screened at 5 years, against threshold probability, for the M0 clinical Cox, M1 clinical plus KLG grade, M2 frontal radiograph and M4 multimodal fusion arms, with treat all and treat none in grey; the marker on treat all is its zero crossing, at the 5-year cumulative incidence of 0.2004. At each threshold the rule flags every patient whose predicted 5-year risk reaches it. Panel B gives the paired differences for the M2 frontal radiograph arm, against treat all and against M0 clinical Cox, with pointwise 2,000-replicate percentile bootstrap 95 percent intervals, unadjusted across thresholds, and a zero reference line. Panel A carries no intervals: marginal intervals over the same patients overlap where the paired difference between

them does not. Thresholds 0.02 to 0.30 are drawn of the 0.01 to 0.35 the underlying table reports, and each curve stops where its flagged set falls below the 15-event floor. The arms score different populations: 741 patients and 106 events for M0 clinical Cox, 734 and 106 for M2 frontal radiograph, and 707 and 98 for the set every arm scores; Table 3 gives them per arm. Panel A draws M0 clinical Cox's treat all while Panel B's difference is taken against M2 frontal radiograph's own, so the two do not line up exactly. M2 frontal radiograph under-predicts 5-year risk by 5.6 percentage points in the large, and net benefit is sensitive to calibration where discrimination is not, so the horizontal axis is a decision-rule parameter rather than a true risk. The estimator, the threshold grid, the plotted window and the sensitivity curve beside this one are set out in the deviation register. These are out-of-sample estimates: the test split was read once, after every model and every hyperparameter choice had been frozen.

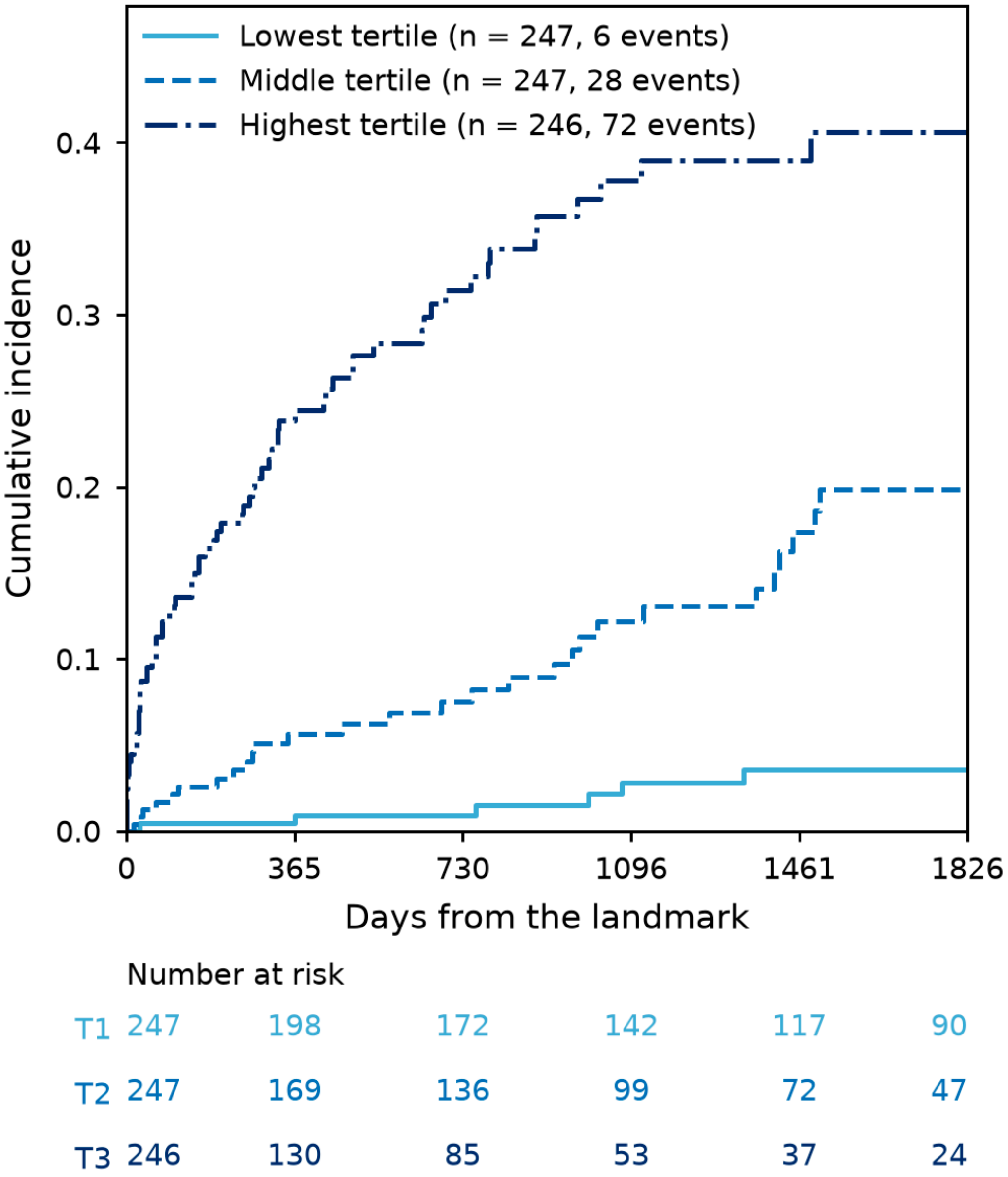

Figure S4. Cumulative incidence of contralateral knee arthroplasty by predicted 5-year risk tertile. Cumulative incidence of contralateral knee arthroplasty by predicted risk tertile. Test patients were split into tertiles of predicted 5-year risk from the multimodal fusion model, and each curve is the Kaplan-

Meier cumulative incidence, one minus the survival function, from the landmark through day 1826. The number of patients remaining at risk in each tertile is given beneath the horizontal axis. Death is not ascertainable in this data source, so mortality acts as an unmeasured competing event; these are therefore cause-agnostic cumulative incidences rather than cause-specific ones, and where competing mortality is appreciable they will overstate what a competing-risk estimator would give. The three curves rest on 740 patients and 106 events in total, so the separation between tertiles should be read as exploratory rather than as a precise estimate of absolute risk.

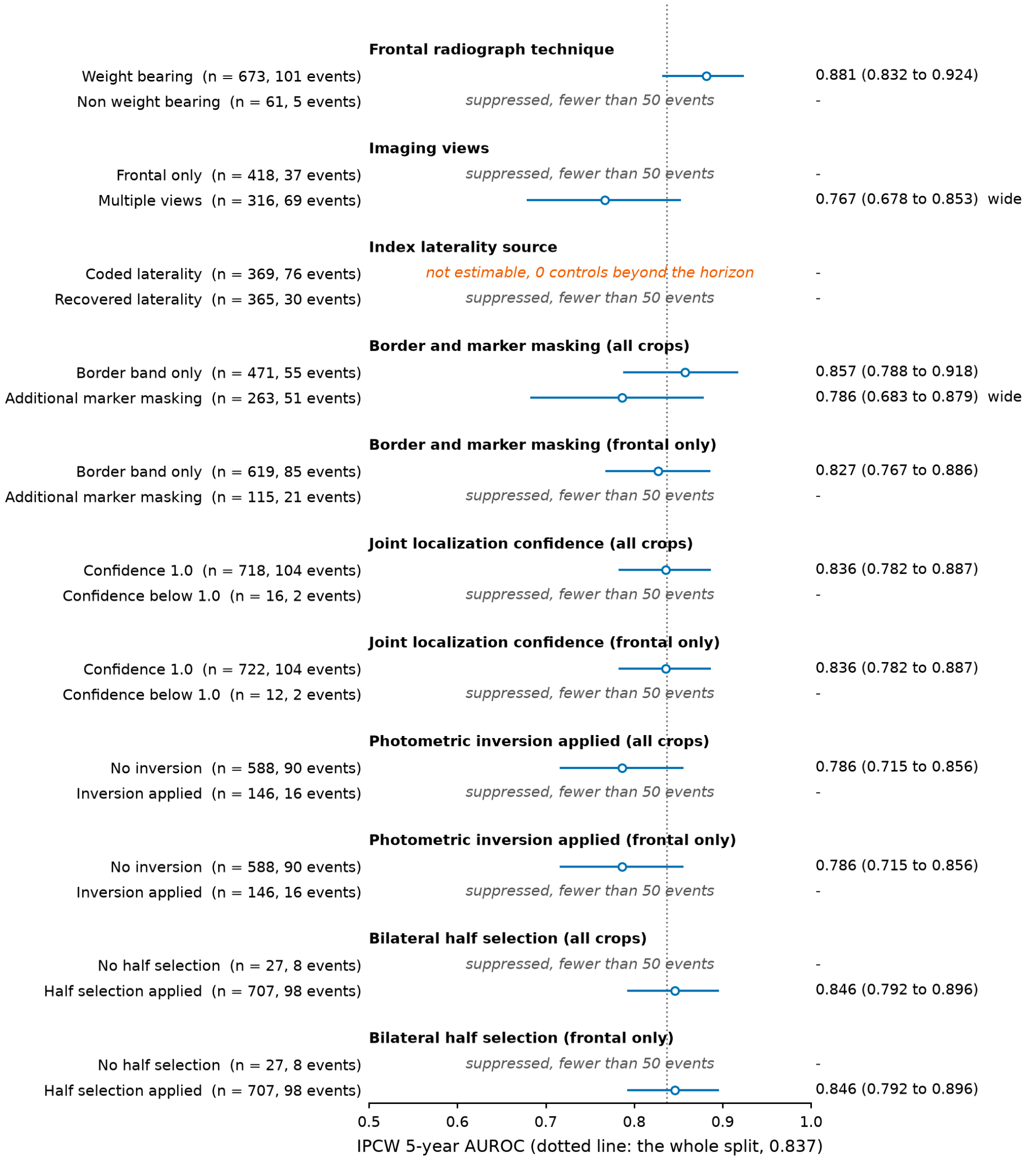

Figure S5. Discrimination within strata of image acquisition and image quality. Imaging robustness of the frontal-radiograph model on the test split. Each row is one level of one stratum. A level is drawn as an estimate with its 2,000-replicate percentile bootstrap 95 percent interval only where it clears the pre-specified 50-event floor and the estimator is defined; the other two states are marked rather than left blank. 'Suppressed' means the level carries fewer than the 50 events the analysis plan requires. 'Not estimable' means something different and stronger: the 5-year IPCW area under the curve compares patients with an event by the horizon against patients observed event free beyond it, and only 162 of

741 patients in this split reach that day, so several levels clear the event floor and still hold no such control at all. Those cells are undefined, not imprecise. The image-quality strata are reported twice, over ALL of a patient's crops and over the frontal crop alone; the two are different denominators and, for masking, different conclusions, so they are labelled and never averaged. Joint-localization method is not drawn separately: a crop confidence below one holds if and only if the intensity-profile localizer was used, so the two are one partition and not two findings. Only one stratum family has two estimable levels, so most rows compare one stratum against the whole split rather than one stratum against another. Acquisition era is omitted from this figure: the de-identified study date carries a per-patient random shift whose cross-patient comparability has never been confirmed in writing (deviation D17), and era is almost perfectly confounded with follow-up length in this cohort. Equipment, manufacturer and acquisition site were never released with the data and cannot be evaluated at all.

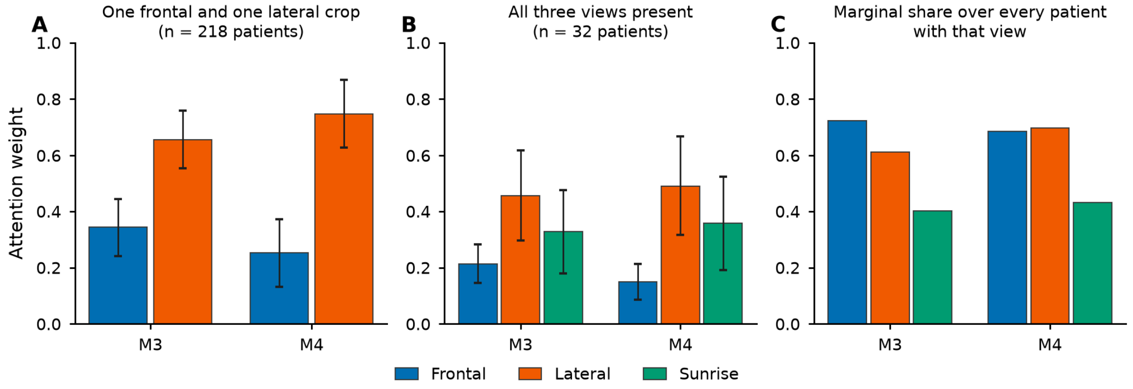

Figure S6. Attention weight assigned to each radiographic view by the multi-view models. Per-view attention weights read out of the frozen aggregators on the test split. Weights sum to one over the views a patient actually contributed, so a patient holding only a frontal radiograph contributes a weight of one to it trivially; the comparison that means anything is therefore WITHIN patients holding one crop of each view. Panel A gives the mean weight in the 218 patients holding exactly one frontal and one lateral crop, with standard deviations; the lateral view outweighs the frontal in 95.4 percent of them under the multi-view image model and 96.8 percent under the fusion model. Panel B gives the three-view mean in the 32 patients holding all three views. Panel C gives the marginal share over every patient with the view, which is the number the trivial single-view weights inflate and is shown so the two can be told apart. The aggregator is not ignoring the additional views; where a lateral radiograph exists it takes the majority of the weight. The frontal-only result in the main text is a statement about a population in which most patients have no second view, not a statement that the second view is uninformative.
