# Supplementary material for: Deep Learning Analysis of Routine Preoperative Knee Radiographs for Five-Year Prediction of Contralateral Arthroplasty
Source: J Imaging. 2026 Sep 30;12(10):474. doi: 10.3390/jimaging12100474 (PMC13705656; doi:10.3390/jimaging12100474)
Supplement: Supplementary file 1 [file jimaging-12-00474-s001.zip › TRIPOD_AI_completed_v6.pdf]

Completed TRIPOD+AI Checklist

Manuscript: Single-View Deep Learning Analysis of Routine Knee Radiographs for Five-Year Prediction of Contralateral Arthroplasty (v6 resubmission to Journal of Imaging)

TRIPOD+AI version 11 January 2024 | Development and internal validation study

Completion convention (rewritten 2026-08-12, counts re-derived from this file's own status cells on 2026-08-13): 39 Yes, 7 No, 6 NA, over 52 rows. No status moved on 2026-08-13. "Yes" means every component of the item is addressed somewhere in the submission. "No" means at least one component is not addressed or was not performed; the entry names which component and what would make it Yes. "NA" means the item's precondition does not hold for this study design. The submission is two documents and locators name which one: Manuscript is [c] v6 MRKR_contralateral_TKA.docx and Supplementary Materials is [supp] v6 supplementary-material.docx. Within the supplement, Supplementary Methods S1 to S8 carry the same eight headings as the Methods and Supplementary Results S1 to S9 follow the order of the Results; where a Supplementary Methods paragraph is named, the name given is that paragraph's bold lead-in. Locators are section based and never page numbers, because page numbers depend on the typeset proof and a wrong page number is worse than none. Two rows moved when every row was re-audited against the shortened body, and one of those two has since been closed by a restored sentence. Item 19 was NA and is now No: nothing in either document states that there was no patient or public involvement, and this item, like item 18d, is satisfied either by details of involvement or by an explicit statement of none, so the previous entry asserted a sentence the manuscript does not contain. Item 27b was moved to No when the length cut removed the sentence that carried the user role, and is Yes again: the Discussion's fourth paragraph now ends by stating that the intended use is informational, that a clinician with musculoskeletal imaging expertise selects the input radiograph, and that this clinician weighs the predicted risk against their own read, which answers both components of the item. Items 5b, 7, 18c, 18d, 18f and 22 remain No. Item 7's evidence changed again on 2026-08-13 and its status still did not: the protocol section-23 two-reviewer image review was performed and now reports raw agreement, Cohen kappa and a critical-error rate inside its threshold (deviation D40), which closes the first of the two components this row was No on. The second is untouched. The item asks for pre-processing and quality checking including whether this was similar across relevant sociodemographic groups, and crop quality has still never been compared across sociodemographic groups: the review's sample was stratified on split, view, contralateral side and bilateral or unilateral acquisition, not on any demographic variable, and only summary statistics were returned, so no such comparison can be drawn from it. CLAIM item 18 does move to Yes on this review, because that item asks only for measures of rater variability; the two checklists differ here because the two items ask for different things. The other five are unchanged. The seven rows that changed status in the previous pass (3c, 5b, 7, 18c, 18d, 18f and 22) are unchanged here. The manuscript now carries only Tables 1 to 3 and Figures 1 to 4; the three tables numbered 4, 5 and 6 in the previous version are Supplementary Tables S3, S4 and S5 respectively and are cited under those names, so no locator in this file names a Table above 3. Item 23a previously cited the old table numbers 4, 5 and 6 and has been corrected; the claim carried forward from the manuscript-rebuild handoff, that this checklist cited only Tables 1 and 3 and needed no table renumbering, was wrong. The green and amber shading of the Status column now follows the status in every row; six rows (3c, 7, 18c, 18f, 22 and 27b) carried shading left over from the status they held in the v5 checklist, and all six were brought into line at this pass. The Methods subsection headings are unchanged; all eight Results subsection headings changed in the cut and are named as the manuscript now heads them.

Section / topic	Item	D / E	Checklist item	Status	Reported in (document and section)	Evidence in the submission
TITLE	**1**	D;E	Identify the study as developing or evaluating the performance of a multivariable prediction model, the target population, and the outcome to be predicted.	**Yes**	Manuscript, Title page, title	Title identifies single-view deep-learning prediction of contralateral TKA from routine knee radiographs, the target population and the 5-year outcome.
ABSTRACT	**2**	D;E	See TRIPOD+AI for Abstracts checklist.	**Yes**	Manuscript, Abstract	Structured abstract reports background, cohort, model arms, evaluation method, results including the attribution and view findings, and conclusions. The leakage controls are not in the abstract; they are in Manuscript, Results, Where the model looks, and what it is not reading, in Manuscript, Discussion, second paragraph, and in Supplementary Materials, Supplementary Table S3 Panel B.
INTRODUCTION - Background	**3a**	D;E	Explain the healthcare context, including whether diagnostic or prognostic, and the rationale for developing or evaluating the prediction model, including references to existing models.	**Yes**	Manuscript, Introduction, first to third paragraphs	Prognostic context, clinical need, prior predictors and prior machine-learning models are described, and the revision states the three imaging questions the study addresses in the fourth paragraph.

Section / topic	Item	D / E	Checklist item	Status	Reported in (document and section)	Evidence in the submission
INTRODUCTION - Background	**3b**	D;E	Describe the target population and intended purpose of the prediction model in the context of the care pathway, including its intended users.	**Yes**	Manuscript, Introduction, first and fourth paragraphs; Manuscript, Discussion, fourth paragraph	Target population is adults undergoing unilateral TKA; intended use is individualized counselling and surgical planning by the treating team, with the Discussion stating that the findings support stratification rather than quantification and that clinical use must wait for external validation.
INTRODUCTION - Background	**3c**	D;E	Describe any known health inequalities between sociodemographic groups.	**Yes**	Manuscript, Discussion, fifth paragraph; Supplementary Materials, Supplementary Methods S1, Splitting; Supplementary Materials, Supplementary Results S7	Yes. The Discussion states that access to and utilisation of knee arthroplasty differ by race and by sex, citing a study of racial disparities in pain and total knee arthroplasty across knee osteoarthritis phenotypes and the New England Journal of Medicine analysis of sex differences in the rate of hip and knee arthroplasty use, and says in the same sentence that this study's subgroup analyses were underpowered to test performance across those groups, so the report cannot speak to whether the model would perform equitably. Partitioning was stratified by sex and major race group (Supplementary Methods S1, Splitting), the 13 pre-specified equity subgroup estimates and the 7 suppressed below the event floor are in Supplementary Results S7, and adequately powered fairness analysis is named as a required next step. Recorded as Not Applicable in v5, which was wrong: the item applies to any prediction model in this care pathway. Two qualifications travel with the Yes and are stated rather than left to inference. The description is one sentence and it sits in the Discussion's limitations rather than in the Introduction's background, which is where TRIPOD+AI places the item; and no analysis of how those inequalities bear on this model's behaviour is claimed beyond the underpowered-subgroup statement, because none was possible at these subgroup event counts (see item 14).

Section / topic	Item	D / E	Checklist item	Status	Reported in (document and section)	Evidence in the submission
INTRODUCTION - Objectives	**4**	D;E	Specify the study objectives, including whether the study describes development or validation of a prediction model, or both.	**Yes**	Manuscript, Introduction, fourth and fifth paragraphs; Manuscript, Methods, Image interpretability and leakage controls, first paragraph; Supplementary Materials, Supplementary Methods S6, Status (deviations D35 and D36)	Objectives state model development and internal validation, comparator benchmarking, and assessment of additional views and clinical data. The revision states that the single-view contrasts, the Kellgren-Lawrence-stratified analyses, the interpretability package and the expanded robustness strata are post hoc and exploratory (deviations D35, D36).
METHODS - Data	**5a**	D;E	Describe the sources of data separately for the development and evaluation datasets, the rationale for using these data, and representativeness of the data.	**Yes**	Manuscript, Title page, Data Availability Statement; Manuscript, Methods, Radiographic dataset and image selection; Supplementary Materials, Supplementary Methods S1, Eligibility, Image selection and Splitting	The de-identified MRKR single-institution radiograph archive and the locked development and test partitions are described, with the public access route and license. After the length cut the body states the source, the eligibility rule and the locked stratified partition, and the Supplementary Methods carry the full eligibility criteria, the view preference order and the stratification variables.

Section / topic	Item	D / E	Checklist item	Status	Reported in (document and section)	Evidence in the submission
METHODS - Data	**5b**	D;E	Specify the dates of collected participant data, including start and end of participant accrual and, if applicable, end of follow-up.	**No**	Not stated. Nearest text: Manuscript, Methods, Radiographic dataset and image selection, first paragraph (follow-up definitions); Manuscript, Results, Robustness strata (the unconfirmed per-patient date shift, deviation D17); Manuscript, Table 1 (observed follow-up from landmark); Supplementary Materials, Supplementary Methods S7, Acquisition era (deviation D17); Supplementary Materials, Supplementary Table S2 note	No. Accrual start and end dates are not stated. The only dates available (StudyDate_anon, spanning study years 2007 to 2021) carry a PER-PATIENT RANDOM SHIFT, so absolute calendar dates are not comparable across patients and no true accrual window can be reported; only within-patient intervals are valid. Protocol section 17 requires written confirmation that de-identified dates are comparable across patients and it has never been obtained (deviation D17). Follow-up definitions, the day-90 landmark, the 5-year horizon and median follow-up are reported. Previously recorded as Not Applicable; the item is applicable and the honest answer is that the dates cannot be stated. It becomes Yes when the Methods state the shifted study-year span, the follow-up end and the shift caveat explicitly.
METHODS - Participants	**6a**	D;E	Specify key elements of the study setting, including the number and location of centres.	**Yes**	Manuscript, Methods, Radiographic dataset and image selection, first paragraph; Supplementary Materials, Supplementary Table S2, Strata that could not be constructed at all	The source is identified as the Emory Knee Radiograph dataset from a single institution; no site or institution identifier was released, so no centre-level description is possible, and the supplement states that absence and its consequence.

Section / topic	Item	D / E	Checklist item	Status	Reported in (document and section)	Evidence in the submission
METHODS - Participants	**6b**	D;E	Describe the eligibility criteria for study participants.	Yes	Manuscript, Methods, Radiographic dataset and image selection, first paragraph; Supplementary Materials, Supplementary Methods S1, Eligibility and Image selection; Supplementary Materials, Supplementary Figure S2	Eligibility and exclusions are described in one sentence in the body, in full in Supplementary Methods S1, including the infection and osteomyelitis exclusion, and displayed in the cohort flow figure.
METHODS - Participants	**6c**	D;E	Give details of any treatments received and how they were handled during model development or evaluation, if relevant.	Yes	Manuscript, Methods, Radiographic dataset and image selection, first paragraph	All participants underwent an index unilateral TKA; the index procedure defines the day-90 landmark and the prediction setting.

Section / topic	Item	D / E	Checklist item	Status	Reported in (document and section)	Evidence in the submission
METHODS - Data preparation	7	D;E	Describe data pre-processing and quality checking, including whether this was similar across relevant sociodemographic groups.	No	Manuscript, Methods, Image preprocessing; Manuscript, Methods, Image-quality and laterality verification, second paragraph; Manuscript, Results, Manual review and burned-in markers; Supplementary Materials, Supplementary Methods S2, Section 23 manual image review (deviation D40), The result, reported as ranges and bounds across the six items, Section 7 laterality audit (deviation D22) and Burned-in markers, four automated rates and one human read; Supplementary Materials, Supplementary Methods S3 (all five paragraphs); Supplementary Materials, Supplementary Results S1	No, in two components. Clinical imputation, radiograph cropping, half-selection, mirroring, resampling, border-band and burned-in-marker masking, augmentation, crop admissibility and the automated quality gates are described in full across the body and Supplementary Methods S3 and S8, and the revision discloses that the masked band is not exactly zero on 145 of 1,216 test crops (11.9%), a protocol section-13 contract violation whose direct measurement is a change in discrimination of -0.0002 (95% CI -0.0009 to 0.0000) when the residue is re-zeroed (deviation D39). This row was No in two components. The first is now met and the second is not, so the status is unchanged and the ground narrows. First, the protocol section-23 manual quality review by two independent reviewers was PERFORMED on 2026-08-13 (deviation D40, which withdraws the decline it registered on 2026-08-12). Two reviewers with musculoskeletal imaging experience scored the 400-image sample independently: raw agreement 95% to 97.9% and Cohen kappa 0.81 to 0.89 across the six items, and a critical-error rate of 0.5% to 1.0% against the pre-specified 2% threshold, which it clears. A completed human quality check therefore now accompanies the preprocessing, and CLAIM item 18 moves to Yes on it. Second, and this is why this row does not move, preprocessing quality has still never been compared across sociodemographic groups, so the item's 'similar across relevant sociodemographic groups' clause remains unaddressed. The review does not supply it: its sample was stratified on split, view, contralateral side and bilateral or unilateral acquisition, not on any demographic variable, and the per-image adjudications are held on a secure institutional system and were returned as summary statistics, so no demographic breakdown can be derived from them. One route to Yes remains open and the row states it: compare crop quality across the recorded demographic groups. The protocol section 7 laterality audit WAS also completed, at 98.0% agreement with the index-side recovery rule on 196 of 200 development patients (Wilson 95% CI 95.0 to 99.2, deviation D22), but it audits index-side recovery rather than preprocessing quality and does not bear on this item.

Section / topic	Item	D / E	Checklist item	Status	Reported in (document and section)	Evidence in the submission
METHODS - Outcome	**8a**	D;E	Clearly define the predicted outcome and time horizon, including how and when assessed, the rationale for choosing it, and whether assessment was consistent across sociodemographic groups.	Yes	Manuscript, Methods, Radiographic dataset and image selection, first paragraph; Supplementary Materials, Supplementary Methods S1, Eligibility	Outcome is contralateral TKA within 5 years after a day-90 landmark, ascertained algorithmically from linked CPT 27447 records and censored at day 1826. The same record-based algorithm was applied identically to every patient and every partition, so outcome assessment is consistent across sociodemographic groups by construction.
METHODS - Outcome	**8b**	D;E	If outcome assessment requires subjective interpretation, describe the qualifications and demographic characteristics of outcome assessors.	NA	NA (see Supplementary Materials, Supplementary Methods S2, Section 23 manual image review (deviation D40), for the unadjudicated section 23(ii) outcome-record audit)	The outcome is electronically ascertained from procedure coding and does not require subjective assessor interpretation, so the item's precondition does not hold. The protocol section-23(ii) review of 200 outcome records, which would validate that ascertainment, is drawn and unadjudicated (deviation D22) and is reported under item 26 as a limitation rather than here. It is the one manual review of the three that remains outstanding; the section-23(i) image review was performed and is reported (deviation D40) and the section 7 laterality audit is complete.
METHODS - Outcome	**8c**	D;E	Report any actions to blind assessment of the outcome to be predicted.	NA	NA	Outcome ascertainment is record based and entirely separate from model fitting; subjective blinded adjudication was not applicable.

Section / topic	Item	D / E	Checklist item	Status	Reported in (document and section)	Evidence in the submission
METHODS - Predictors	9a	D	Describe the choice of initial predictors and any pre-selection before model building.	Yes	Manuscript, Introduction, fifth paragraph; Manuscript, Methods, Radiographic dataset and image selection; Manuscript, Methods, Clinical comparators and statistical analysis, first paragraph; Supplementary Materials, Supplementary Methods S4, The nine arms; Supplementary Materials, Supplementary Methods S8, Comparators (deviations D20, D21, D23 and D24)	Prior literature and routinely recorded clinical variables motivate the predictor set; the image views and the model arms were pre-specified in the protocol's model ladder, which Supplementary Methods S4 sets out arm by arm.

Section / topic	Item	D / E	Checklist item	Status	Reported in (document and section)	Evidence in the submission
METHODS - Predictors	**9b**	D;E	Clearly define all predictors, including how and when measured, and any actions to blind predictor assessment from outcome or other predictors.	**Yes**	Manuscript, Methods, Radiographic dataset and image selection, first paragraph (the 1 to 730 day pre-index window); Manuscript, Methods, Image-quality and laterality verification, first paragraph (the model-inferred annotations and the uncheckable horizontal-flip flag); Manuscript, Methods, Image preprocessing; Manuscript, Methods, Clinical comparators and statistical analysis, first paragraph (deviations D20, D21, D23 and D24); Manuscript, Table 1; Supplementary Materials, Supplementary Methods S2, Dataset-supplied image annotations; Supplementary Materials, Supplementary Methods S8, Comparators	Predictor timing, lookback definitions, the 1 to 730 day pre-index image window, the clinical features, the Kellgren-Lawrence grade and the image preprocessing are described. The revision states which predictors are model-derived rather than measured: the contralateral Kellgren-Lawrence grade is dataset-inferred by an automated model, and the per-image laterality, view position and horizontal-flip labels are MRKR model outputs, with the horizontal-flip flag driving the bilateral half-select on 287 films, 63 of them in the test set. None of these has been validated by human review (deviation D22). Two departures from the pre-specified predictor definitions remain (deviations D20 comorbidity lookback and D21 pain aggregation), are flagged in the body and are set out in full in Supplementary Methods S8.

Section / topic	Item	D / E	Checklist item	Status	Reported in (document and section)	Evidence in the submission
METHODS - Predictors	**9c**	D;E	If predictor measurement requires subjective interpretation, describe the qualifications and demographic characteristics of predictor assessors.	**NA**	NA	Predictor measurement requires no subjective human interpretation and no study-specific assessor panel was used, so the item's precondition does not hold. The predictors that would ordinarily be subjective (Kellgren-Lawrence grade, view position, laterality) are automated model outputs; their accuracy is unvalidated and is reported under items 9b and 26, and their absence of a rater-variability estimate is reported under CLAIM item 18.
METHODS - Sample size	**10**	D;E	Explain how study size was arrived at and justify that it was sufficient. Include details of any sample-size calculation.	**Yes**	Manuscript, Methods, Clinical comparators and statistical analysis, final paragraph; Manuscript, Results, Prediction within Kellgren-Lawrence strata; Supplementary Materials, Supplementary Methods S8, Prediction within radiographic severity	The eligible cohort determined the study size; participant and event counts are reported and the sample was checked against Riley criteria. The revision also states the arithmetic limit the same sample imposes on stratified analysis: the 98 KLG-eligible events cannot support two strata each clearing the pre-specified 50-event floor, which would need at least 100, so at most one within-grade stratum is estimable under any partition.

Section / topic	Item	D / E	Checklist item	Status	Reported in (document and section)	Evidence in the submission
METHODS - Missing data	**11**	D;E	Describe how missing data were handled and provide reasons for omitting any data.	Yes	Manuscript, Table 1 and its note (the missingness column, before imputation); Manuscript, Results, Prediction within Kellgren-Lawrence strata (707 patients with a recorded grade); Supplementary Materials, Supplementary Methods S8, Comparators (training-split median or mode imputation) and Prediction within radiographic severity (the grade missing for 34 of 741); Supplementary Materials, Supplementary Results S1	Training-split median and mode imputation is described; Kellgren-Lawrence and crop-related exclusions and missingness percentages are reported, including the 34 of 741 test patients with no recorded contralateral grade. After the length cut the imputation rule itself sits in Supplementary Methods S8 rather than in the body, which retains only the phrase before imputation in the Table 1 note.

Section / topic	Item	D / E	Checklist item	Status	Reported in (document and section)	Evidence in the submission
METHODS - Analytical methods	**12a**	D	Describe how data were used for model development and evaluation, including partitioning and sample-size considerations.	Yes	Manuscript, Methods, Radiographic dataset and image selection, second paragraph; Manuscript, Methods, Imaging robustness and ablation analyses (deviation D35); Supplementary Materials, Supplementary Methods S1, Splitting; Supplementary Materials, Supplementary Methods S7, Strata (deviation D35)	A locked 70% training, 10% validation and 20% test partition is described, including the single scored read of the test split and the fact that every revision-stage analysis is a further reading of predictions that already existed on it (deviation D35). The stratification variables, 5-year event status, sex and major race group, are named in Supplementary Methods S1.
METHODS - Analytical methods	**12b**	D	Describe how predictors were handled in analyses, including functional form, rescaling, transformation, or standardisation.	Yes	Manuscript, Methods, Image preprocessing; Manuscript, Methods, Clinical comparators and statistical analysis, first and third paragraphs; Supplementary Materials, Supplementary Methods S3; Supplementary Materials, Supplementary Methods S8, Comparators and Prediction within radiographic severity	Clinical imputation and image cropping, mirroring, resampling, intensity handling and augmentation are specified; the Cox incremental analysis uses the complementary log-log of the predicted 5-year risk standardised to unit SD, which is invariant to the frozen recalibration because that recalibration is affine on exactly that scale.

Section / topic	Item	D / E	Checklist item	Status	Reported in (document and section)	Evidence in the submission
METHODS - Analytical methods	**12c**	D	Specify model type and rationale, all model-building steps, hyperparameter tuning, and internal validation.	Yes	Manuscript, Methods, Network architecture and multi-view aggregation; Manuscript, Methods, Training and internal validation; Supplementary Materials, Supplementary Methods S4, Architecture, The nine arms and Encoder substitution (deviation D28); Supplementary Materials, Supplementary Methods S5, Optimization (deviation D26), Recalibration, Convergence and Environment (deviations D37 and D38)	Penalized Cox comparators, DenseNet121 image models, optimization, the five seeds, early stopping, the validation-fitted recalibration and locked testing are described; the parameter values moved to Supplementary Methods S5 when the body was cut. The ConvNeXt-Tiny robustness arm is keyed r1_densenet_frontal for historical reasons and the architecture label in train_arms.json is authoritative.
METHODS - Analytical methods	**12d**	D;E	Describe if and how heterogeneity in model parameters and performance was handled and quantified across clusters.	NA	NA (see Supplementary Materials, Supplementary Table S2, Strata that could not be constructed at all, and Manuscript, Results, Robustness strata)	The study uses a single-institution dataset with no released site, department or equipment identifier, so no cluster variable exists and no cluster-level heterogeneity analysis is possible. The unavailability and its cause are reported (outputs/tables/v6_robustness_metadata_availability.csv).

Section / topic	Item	D / E	Checklist item	Status	Reported in (document and section)	Evidence in the submission
METHODS - Analytical methods	**12e**	D;E	Specify all measures and plots used, and their rationale, to evaluate performance and compare models.	**Yes**	Manuscript, Methods, Clinical comparators and statistical analysis; Manuscript, Methods, Image interpretability and leakage controls; Manuscript, Methods, Imaging robustness and ablation analyses; Supplementary Materials, Supplementary Methods S6, S7 and S8	Censoring-weighted time-dependent (IPCW) AUROC, Harrell and Uno concordance, calibration, Brier score, paired bootstrap intervals, risk-stratum curves and decision curves are specified. The revision adds Cox hazard ratios per SD with likelihood-ratio tests against a model containing the Kellgren-Lawrence grade, Kaplan-Meier incidence by risk tertile within grade with log-rank tests, region-wise attribution mass with area-normalised enrichment, occlusion deltas in AUROC and in mean predicted risk, view-withholding contrasts and per-view attention weights, each with its rationale and its denominator. The plots are Figures 3 and 4 in the manuscript and Supplementary Figures S1, S3, S4, S5 and S6.
METHODS - Analytical methods	**12f**	E	Describe any model updating, such as recalibration, arising from model evaluation.	**Yes**	Manuscript, Methods, Training and internal validation; Manuscript, Results, Calibration and clinical usefulness; Manuscript, Figure 4 caption; Supplementary Materials, Supplementary Methods S5, Recalibration (the day-1825 coefficients); Supplementary Materials, Supplementary Results S9	Horizon-specific recalibration was fitted on the validation split, frozen before the test read, and its results are reported; text and figure captions state consistently whether displayed predictions are before or after it, and the fitted coefficients are in Supplementary Methods S5.

Section / topic	Item	D / E	Checklist item	Status	Reported in (document and section)	Evidence in the submission
METHODS - Analytical methods	**12g**	E	For model evaluation, describe how model predictions were calculated.	Yes	Manuscript, Methods, Network architecture and multi-view aggregation, first paragraph; Manuscript, Methods, Training and internal validation; Supplementary Materials, Supplementary Methods S4, Architecture; Supplementary Materials, Supplementary Methods S5, Optimization (deviation D26) and Recalibration	Ten six-month conditional hazards are combined into cumulative 5-year risk; ensembles average predicted hazards across the five seeds and convert to risk once, before the frozen recalibration is applied.
METHODS - Class imbalance	**13**	D;E	If class-imbalance methods were used, state why and how, and describe subsequent recalibration.	NA	NA (see Manuscript, Methods, Clinical comparators and statistical analysis, second paragraph, and Supplementary Materials, Supplementary Methods S8, Multiplicity and suppression, for the censoring-weighted estimators and the 50-event floor used instead of resampling)	No dedicated over-sampling, under-sampling or class-weighting procedure was used. The event fraction (106 of 741 test patients) is handled by censoring-weighted estimators and by the pre-specified 50-event suppression floor rather than by resampling, and this is stated.

Section / topic	Item	D / E	Checklist item	Status	Reported in (document and section)	Evidence in the submission
METHODS - Fairness	**14**	D;E	Describe approaches used to address model fairness and their rationale.	**Yes**	Manuscript, Methods, Radiographic dataset and image selection, second paragraph (stratified partition); Manuscript, Methods, Clinical comparators and statistical analysis, second paragraph (the 50-event suppression rule); Manuscript, Discussion, fifth paragraph; Supplementary Materials, Supplementary Methods S1, Splitting; Supplementary Materials, Supplementary Results S7	Partitioning was stratified by sex and major race group, which Supplementary Methods S1 names; pre-specified subgroup estimates use the event-count suppression rule; fairness limitations are discussed. The revision states the limits plainly: no fairness metric such as equalized odds or subgroup calibration was computed, of the 13 pre-specified equity subgroup estimates 7 fall below the 50-event floor and are suppressed while the 6 that clear it run from 0.804 to 0.873 with every interval overlapping every other (Supplementary Results S7), and adequately powered fairness analysis is named as a required next step.

Section / topic	Item	D / E	Checklist item	Status	Reported in (document and section)	Evidence in the submission
METHODS - Model output	15	D	Specify the prediction-model output and provide details and rationale for classification and thresholds.	Yes	Manuscript, Methods, Network architecture and multi-view aggregation, first paragraph; Manuscript, Methods, Clinical comparators and statistical analysis, third paragraph (the classifiable subset and deviation D29); Manuscript, Figure 2 caption (the 0.134 operating point); Manuscript, Results, Prediction within Kellgren-Lawrence strata and Calibration and clinical usefulness; Supplementary Materials, Supplementary Methods S8, Decision-curve analysis (deviation D29) and Classifiable denominators; Supplementary Materials, Supplementary Results S6 and S9	Models output ten interval hazards and a cumulative 5-year risk; risk tertiles and exploratory decision thresholds are described. The revision adds one further operating point, the risk quantile at which predicted positives equal observed cases, which is 0.134 on the published risk, used only to classify the true-positive / false-positive / true-negative / false-negative example cases; it was chosen after the single test read and is exploratory, and it is defined against the 263 of 734 patients classifiable at 5 years rather than against the cohort.

Section / topic	Item	D / E	Checklist item	Status	Reported in (document and section)	Evidence in the submission
METHODS - Training versus evaluation	**16**	D;E	Identify differences between development and evaluation data in setting, eligibility criteria, outcome, and predictors.	**Yes**	Manuscript, Methods, Radiographic dataset and image selection, second paragraph; Manuscript, Table 1	Development and test data come from the same source and criteria; locked split sizes and characteristics are compared in Table 1.
METHODS - Ethical approval	**17**	D;E	Name the approving institutional review board or ethics committee and describe informed consent or waiver.	**Yes**	Manuscript, Title page, Ethical Approval & Informed Consent Statement, Institutional Review Board Statement and Informed Consent Statement	Emory University IRB STUDY00002023 and the waiver of informed consent are reported for the source dataset, with the secondary-analysis determination under 45 CFR 46.102(e) explained.
OPEN SCIENCE - Funding	**18a**	D;E	Give the funding source and the role of funders.	**Yes**	Manuscript, Title page, Funding statement	The manuscript states that the research received no external funding.
OPEN SCIENCE - Conflicts of interest	**18b**	D;E	Declare conflicts of interest and financial disclosures for all authors.	**Yes**	Manuscript, Title page, Declaration of Conflicts of Interest	The authors declare no conflicts of interest.

Section / topic	Item	D / E	Checklist item	Status	Reported in (document and section)	Evidence in the submission
OPEN SCIENCE - Protocol	**18c**	D;E	Indicate where the study protocol can be accessed or state that a protocol was not prepared.	**No**	Not stated. Nearest text: Manuscript, Title page, Code Availability Statement; Manuscript, Methods, Clinical comparators and statistical analysis, final paragraph (deviations are registered); Supplementary Materials, Supplementary Methods S8, Reporting (the deviation register is provided as accompanying material)	No. The submission identifies pre-specified procedures and provides the deviation register (D1 to D40) as accompanying material, but it does not state where the full written study protocol can be accessed. A protocol was prepared before the analysis; it is excluded from the public repository by the project's data-hygiene ignore rules and is not deposited anywhere public. It becomes Yes when the manuscript states the protocol's access route, for example deposit alongside the code archive or availability from the corresponding author.
OPEN SCIENCE - Registration	**18d**	D;E	Provide study registration information or state that the study was not registered.	**No**	Not stated anywhere in the manuscript or the supplementary material. It would belong on the Manuscript, Title page beside the other open-science statements	No. The study was not registered in any public registry, and neither document says so. Previously recorded as Not Applicable, which was wrong: this item is always applicable because it is satisfied either by registration details OR by an explicit statement of non-registration. It becomes Yes when one sentence stating that the study was not registered is added.

Section / topic	Item	D / E	Checklist item	Status	Reported in (document and section)	Evidence in the submission
OPEN SCIENCE - Data sharing	**18e**	D;E	Provide details of study-data availability.	**Yes**	Manuscript, Title page, Data Availability Statement	MRKR access, licensing, documentation, restrictions and the availability of aggregate and derived outputs are described, with patient-level derived data withheld and available on reasonable request. The withholding is now an explicit, documented policy in the public repository rather than an accident of a local ignore file. At commit b568820, pushed 2026-08-11, the repository's committed .gitignore names four per-patient tables (interp_attribution_per_image.csv, interp_attribution_registered.csv, interp_attribution_sample.csv and interp_panel_manifest.csv) and the whole outputs/interpretability/ directory, and states in the file why each is excluded; the aggregate interp_ tables (summary, regions, occlusion, view_ablation, attention_*, sanity_checks, risk_provenance) carry no identifiers and are committed. This corrects the previous entry, which said that the public repository contained no rule of this kind. Those files are patient-level rather than aggregate, carrying one row per sampled patient with the de-identified identifier, predicted risk, event status and follow-up time, so they fall under the same withholding policy the Data Availability Statement describes and are available on reasonable request. The one thing still outstanding is archival: no new Zenodo version has been minted over the pushed commit, so the aggregate v6 outputs are reachable through GitHub but not through the DOI the manuscript cites; see item 18f.
OPEN SCIENCE - Code sharing	**18f**	D;E	Provide details of analytical-code availability.	**No**	Manuscript, Title page, Code Availability Statement	No. A public GitHub repository, MIT License and archived Zenodo DOI are cited. The GitHub half is now met: at commit b568820, pushed 2026-08-11, the repository holds src/interpretability.py (the attribution, occlusion, negative-control, sanity-check and attention package), src/v6_analyses.py (the single-view contrasts and the Kellgren-Lawrence-stratified analyses) and src/manuscript_figures.py, together with the aggregate v6 output tables. The archival half is not. The Statement also cites doi 10.5281/zenodo.21712883, which archives the pre-revision commit and contains none of those modules, and no new archived version has been minted, so a reader who follows the persistent identifier the manuscript gives still reaches code that does not produce the reported analyses. It becomes Yes when a new archived version is minted over commit b568820 or later and the manuscript cites that new DOI rather than the one covering the v5 code.

Section / topic	Item	D / E	Checklist item	Status	Reported in (document and section)	Evidence in the submission
PATIENT & PUBLIC INVOLVEMENT	**19**	D;E	Provide details of patient and public involvement or state no involvement.	No	Not stated anywhere in the manuscript or the supplementary material. It would belong on the Manuscript, Title page beside the other open-science statements	No, and this row moved from NA at this pass. This was a retrospective secondary analysis of de-identified data with no participant contact or intervention, so no patient or public involvement was possible; but the item is satisfied either by details of involvement OR by an explicit statement that there was none, exactly as item 18d is satisfied either by registration details or by a statement of non-registration. Neither document contains any such statement: the strings 'patient and public' and 'public involvement' occur nowhere in either file, and they did not occur in the v5 manuscript either, so this is a standing omission rather than a casualty of the length cut. The previous entry recorded NA and asserted that the absence of involvement 'is stated', which the documents do not support. It becomes Yes when one sentence stating that there was no patient or public involvement in the design, conduct or reporting of this study is added beside the other open-science statements.
RESULTS - Participants	**20a**	D;E	Describe participant flow, numbers with and without the outcome, and follow-up time. A diagram may be helpful.	Yes	Manuscript, Results, Cohort and image data; Supplementary Materials, Supplementary Results S1; Supplementary Materials, Supplementary Figure S2; Manuscript, Table 1 (observed follow-up)	Results report cohort and event counts, 3,709 landmark-cohort patients giving 2,968 development patients with 427 events and 741 test patients with 106, and the flow figure gives detailed flow through eligibility and crop processing, including the one test patient with no usable contralateral image, who was event free. Follow-up time is in Table 1.
RESULTS - Participants	**20b**	D;E	Report overall and data-source characteristics, including dates, predictors, demographics, treatments, sample size, events, follow-up, missing data, and demographic differences.	Yes	Manuscript, Table 1 and its note; Manuscript, Results, Cohort and image data; Supplementary Materials, Supplementary Results S1	Table 1 reports development and test demographics, predictors, follow-up and missingness; event counts are reported in Results and in the performance tables, and the source-archive denominator of 83,011 patients is in Supplementary Results S1. Calendar accrual dates are not reportable; see item 5b.
RESULTS - Participants	**20c**	E	For model evaluation, compare the distribution of important predictors and outcome with the development data.	Yes	Manuscript, Table 1	Table 1 directly compares the development and locked test cohorts.

Section / topic	Item	D / E	Checklist item	Status	Reported in (document and section)	Evidence in the submission
RESULTS - Model development	21	D;E	Specify participant and outcome-event counts in each analysis.	Yes	Manuscript, Tables 1, 2 and 3; Manuscript, Results, all eight subsections; Supplementary Materials, Supplementary Tables S1 to S5; Supplementary Materials, Supplementary Results S1 to S9; Supplementary Materials, Supplementary Methods S1, The two frontal-only counts	Counts are given for development, validation, test, each model arm, each common calibration sample and each decision-curve analysis. The revision reports the denominator of every new estimate explicitly, including the 734-patient occlusion roster, the 858 Grad-CAM crops, the 230 integrated-gradient crops, the 60-crop sanity sample against its 734-patient discrimination denominator, and the 315-patient paired view-ablation roster, which is the set contributing both a frontal and at least one non-frontal radiograph and is distinct from the 316 patients holding two or more distinct views, the 321 contributing any non-frontal crop and the 322 whose selected study was not frontal-only. Supplementary Methods S1, The two frontal-only counts, and the Supplementary Table S4 Panel B note set all five denominators side by side.
RESULTS - Model specification	22	D	Provide details of the full prediction model to allow predictions in new individuals and third-party evaluation, including access or reuse restrictions.	No	Manuscript, Title page, Code Availability Statement (the weight restriction is not stated there); Manuscript, Methods, Network architecture and multi-view aggregation; Manuscript, Methods, Training and internal validation; Supplementary Materials, Supplementary Methods S4 and S5	No. The Methods and Supplementary Methods S4 and S5 specify the architecture, the interval-hazard head and the risk calculation, and the analysis code is linked and licensed, but the trained model weights are not shared and the manuscript does not state that restriction. Without the weights a third party can reproduce the method but cannot make predictions in new individuals or evaluate the reported model. The record that fixes the training and scoring environment (train_arms.json) is also in a git-ignored directory and is not in the public release (deviation D38). The repository push of 2026-08-11 does not bear on this row: it added analysis code, not weights. It becomes Yes when the manuscript states the weight-availability restriction and the request route explicitly, which the item permits.

Section / topic	Item	D / E	Checklist item	Status	Reported in (document and section)	Evidence in the submission
RESULTS - Model performance	**23a**	D;E	Report performance estimates with confidence intervals, including key subgroups, and consider plots.	Yes	Manuscript, Tables 2 and 3; Supplementary Materials, Supplementary Tables S1, S2, S3, S4 and S5; Manuscript, Results, all eight subsections; Supplementary Materials, Supplementary Results S3, S4, S6, S7, S8 and S9	Discrimination, calibration, Brier score, paired contrasts, decision curves, risk strata and eligible subgroup estimates are reported with intervals. The revision adds the post hoc single-view contrasts (M2 versus M0 0.162, 95% CI 0.088 to 0.233; M2 versus M1 0.114, 0.060 to 0.170), the Kellgren-Lawrence-stratified Cox and tertile results, the imaging robustness strata and the interpretability deltas, each with intervals, event counts and the suppression rule honoured. Of the 136 stratum cells across the two image arms, 74 carry no estimate, 68 suppressed by the 50-event floor and 6 undefined for want of controls beyond the horizon, and each is labelled with which of the two it is (Supplementary Results S7, Supplementary Table S2 note). In Supplementary Table S5 Panel B four of the six within-grade discrimination cells are suppressed below the same floor, which the panel note explains is arithmetic rather than a shortfall of effort.
RESULTS - Model performance	**23b**	D;E	If examined, report heterogeneity in model performance across clusters.	NA	NA (see Supplementary Materials, Supplementary Table S2, Strata that could not be constructed at all)	Cluster-level performance heterogeneity was not examined and cannot be: this is a single-institution analysis and no site, department or equipment identifier was released.
RESULTS - Model updating	**24**	E	Report results from model updating, including the updated model and subsequent performance.	Yes	Manuscript, Results, Calibration and clinical usefulness; Manuscript, Table 3; Manuscript, Figure 4; Manuscript, Discussion, fifth paragraph (the tolerance near 0.006); Supplementary Materials, Supplementary Table S1; Supplementary Materials, Supplementary Results S9	Validation-based recalibration and the subsequent test-split calibration slope, calibration-in-the-large, Brier score and calibration plot are reported. The calibration slope is the least stable published statistic and carries a tolerance near 0.006 because the published estimates came from a cloud graphics processor under float16 autocast and the sealed-read artifact records no interpreter (deviation D37), so it is quoted to two decimals.

Section / topic	Item	D / E	Checklist item	Status	Reported in (document and section)	Evidence in the submission
DISCUSSION - Interpretation	**25**	D;E	Give an overall interpretation of the main results, including fairness, in the context of objectives and previous studies.	Yes	Manuscript, Discussion, first to fourth paragraphs; Manuscript, Results, What additional views contribute; Supplementary Materials, Supplementary Table S4 Panels A and B; Supplementary Materials, Supplementary Results S4	The Discussion interprets discrimination, the value of a single frontal view, clinical context, prior literature and fairness limitations. The revision constrains the view claim to what the two estimands support: a frontal-only model matched the multi-view and multimodal arms across the whole cohort (0.837 versus 0.840 and 0.844) because 419 of the 740 test patients with a prepared crop, 56.6%, contributed a frontal radiograph and nothing else, while withholding the lateral and sunrise views from the frozen all-views network cost 0.078 AUROC (95% CI 0.012 to 0.142) on the 315 patients who contributed both a frontal and a non-frontal crop, and the attention pool gave the lateral view the majority of its weight whenever one existed, 0.656 in the multi-view model and 0.748 in the fusion model. Only the sunrise view added nothing detectable, on the 37 of 740 patients who had one. The claim that additional views add nothing measurable is not supported by these data and is not made.
DISCUSSION - Limitations	**26**	D;E	Discuss limitations and their effects on bias, uncertainty, and generalizability.	Yes	Manuscript, Discussion, fifth paragraph, which is now the whole limitations paragraph; Manuscript, Discussion, second and third paragraphs; Manuscript, Results, Manual review and burned-in markers and Robustness strata; Supplementary Materials, Supplementary Methods S2, S6 and S7	Single-institution design, absence of external validation, missing competing mortality, the outstanding outcome-record review, laterality inference, exploratory comparisons and underpowered subgroups are discussed. The revision adds the post hoc status of every new analysis (D35), the completed section-23 two-reviewer image review, which reports raw agreement of 95% to 97.9% and Cohen kappa of 0.81 to 0.89 across six items, a critical-error rate of 0.5% to 1.0% inside its 2% threshold, and a laterality figure resting on the 318 panels that carry a full film rather than on all 400 (D40), the completed section 7 laterality audit at 98.0% agreement on 200 development patients that validates the recovered index side used for 49.3% of the cohort indirectly rather than directly on the reported split, and the unadjudicated section-23(ii) outcome-record review, now the one manual review still outstanding (D22), the dataset-inferred Kellgren-Lawrence grade, the absence of equipment, manufacturer and site metadata, the uninterpretable acquisition-era stratum (D17), the non-zero border band on 11.9% of test crops (D39), the degenerate border-only negative control, the unrun label-randomisation check (D36) and the environment-provenance gaps (D37, D38). The three separate limitations paragraphs of the previous version are now one paragraph.

Section / topic	Item	D / E	Checklist item	Status	Reported in (document and section)	Evidence in the submission
DISCUSSION - Usability	**27a**	D	Describe how poor-quality or unavailable input data should be assessed and handled during implementation.	Yes	Manuscript, Methods, Image preprocessing; Manuscript, Methods, Imaging robustness and ablation analyses; Manuscript, Results, Robustness strata; Manuscript, Results, Where the model looks, and what it is not reading; Supplementary Materials, Supplementary Methods S3 and S7; Supplementary Materials, Supplementary Methods S8, Comparators (the imputation rule); Supplementary Materials, Supplementary Results S7; Supplementary Materials, Supplementary Table S2 and Supplementary Figure S5	Crop admissibility, preprocessing, imputation, missing views, the incomplete quality review and the need to complete it are described. The revision adds measured behaviour under degraded input: discrimination by crop confidence and localization method, by photometric inversion, by bilateral half-selection and by the amount of border and marker masking, and the occlusion results that show how prediction moves when regions of the image are removed. In every one of those families except masking only one level clears the 50-event floor, so these are comparisons of one stratum against the whole split rather than between strata.

Section / topic	Item	D / E	Checklist item	Status	Reported in (document and section)	Evidence in the submission
DISCUSSION - Usability	**27b**	D	Specify whether users must interact in handling inputs or using the model and the expertise required.	Yes	Manuscript, Discussion, fourth paragraph, final sentence, which reads The intended use is informational: a clinician with musculoskeletal imaging expertise selects the input radiograph and weighs the predicted risk against their own read; Manuscript, Discussion, fourth paragraph, which opens These findings support stratification, not quantification; Manuscript, Discussion, fifth paragraph, final sentence; Manuscript, Conclusion	Yes, and this row moved back from No at this pass. Both components of the item are now addressed in one sentence, the final sentence of the Discussion's fourth paragraph. Whether users must interact: the intended use is informational and a clinician selects the input radiograph, so a person handles the input and reads the output rather than the model running on its own. The expertise required: musculoskeletal imaging expertise, which is the qualification protocol section 23 also requires of the reviewers who score these images, and that clinician weighs the predicted risk against their own read of the radiograph. The constraint on how the output may be used is stated in the same paragraph, namely that the model orders risk better than it quantifies it and that counselling on the timing or staging of a second procedure must rest on relative risk and observed incidence within a predicted-risk band rather than on the predicted probability. The fifth paragraph and the Conclusion require external and temporally separated validation, the completed protocol reviews and evidence that acting on predicted risk changes outcomes before clinical use, which forecloses any autonomous use. Item 27a separately describes how poor-quality and unavailable input data are assessed and handled; this row is about the user, not the data.
DISCUSSION - Usability	**27c**	D;E	Discuss next steps for research, particularly applicability and generalizability.	Yes	Manuscript, Discussion, fifth paragraph, final sentence; Manuscript, Conclusion	Next steps are prioritized: external and temporally separated validation, completion of the protocol-specified manual reviews, competing-risk analysis, adequately powered fairness analysis, evidence that acting on predicted risk changes outcomes, and prospective evaluation before deployment. The longer next-steps list of the previous version, which also named the label-randomisation sanity check, an acquisition-era analysis on dates whose cross-patient comparability has been confirmed, and reader-referenced evaluation against a radiologist-assigned Kellgren-Lawrence grade, did not survive the length cut as a list of next steps; the underlying gaps are still stated in Manuscript, Discussion, fifth paragraph, and in Supplementary Materials, Supplementary Methods S6, Randomization checks, and S7, Acquisition era (deviation D17).

Notes

- D = items relevant to prediction-model development; E = items relevant to model evaluation; D;E = applicable to both.
- The study is classified as prediction-model development with internal validation. No external validation is claimed.
- Checklist source: Collins GS, Moons KGM, Dhiman P, et al. TRIPOD+AI statement. BMJ. 2024;385:e078378. doi:10.1136/bmj-2023-078378.
